# C–H-Functionalization logic guides the synthesis of a carbacyclopamine analog

**DOI:** 10.3762/bjoc.10.161

**Published:** 2014-07-09

**Authors:** Sebastian Rabe, Johann Moschner, Marina Bantzi, Philipp Heretsch, Athanassios Giannis

**Affiliations:** 1 Universität Leipzig, Institut für Organische Chemie, Johannisallee 29, D-04103 Leipzig, Germany; 2 Rice University, Department of Chemistry, BioScience Research Collaborative, 6100 Main St., Houston, Texas 77005, United States

**Keywords:** cyclopamine, C–H-functionalization, hedgehog signaling pathway, natural products, steroidal alkaloids

## Abstract

The chemical synthesis of carbacyclopamine analog **2**, a cyclopamine analog with an all-carbon E-ring, is reported. The use of C–H-functionalization logic and further metal-catalyzed transformations allows for a concise entry to this new class of acid-stable cyclopamine analogs.

## Introduction

**Figure 1:**
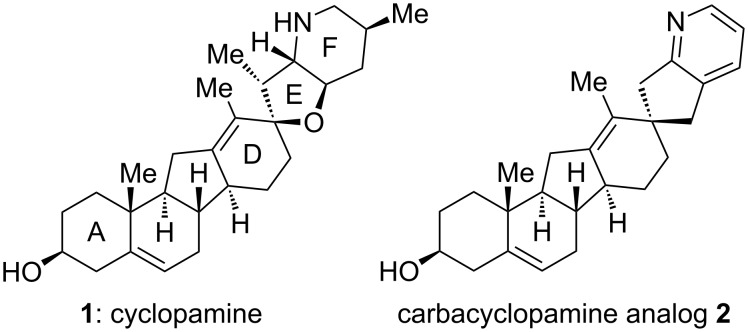
Structures of cyclopamine (**1**) and carbacyclopamine analog **2**.

The isolation of cyclopamine (**1**, Figure 1) nearly 50 years ago followed by the determination of its biological target and pharmacological profile has drawn considerable interest from research groups in biology, chemistry and medicine [1–4]. As the first identified hedgehog signaling inhibitor, cyclopamine exerts its anticancer activity through a novel mechanism of action, which manifests itself in its remarkable activity against several types of human cancer, including medulloblastoma, basal cell carcinoma, and rhabdomyosarcoma [5–10]. The impressive biological activity combined with a unique molecular architecture and the synthetic challenge it poses already let to the first synthesis of cyclopamine by this laboratory employing C–H-functionalization logic [11]. In addition, the rational design and chemical synthesis of several analogs and derivatives by this [12–13] and other groups [14–17] have been disclosed. Here, we report the synthesis of a carbacyclopamine analog (**2**, Figure 1), an analog of the natural product with an all-carbon E-ring and a pyridine F-ring.

Cyclopamine inhibits the 7-transmembrane protein smoothened from converting into its active form [18–19]. Without active smoothened, protein kinases like PKA, GSK3β, and CK1ε phosphorylate the transcription factors Gli2 and Gli3, thereby creating binding sites for the adapter protein β-TrCP [20]. The Gli family of transcription factors acts as an effector of the hedgehog signaling pathway and thus, is associated with a wide array of physiological effects, including cell fate determination, proliferation and patterning. The so-formed Gli/β-TrCP complex becomes subject to ubiquitinylation, mediated by the Cul1-based E3 ligase. Eventually, this results in partial proteosomal degradation to form Gli3-R [21], or in the case of Gli2 [22–23], in complete degradation. In addition, the Gli3-R factor acts as a transcription inhibitor of hedgehog-response genes [10].

As part of our continuing work on acid-stable cyclopamine analogs [12–13] we have now focused on the role of the allylic ether oxygen in the acid-mediated E-ring cleavage and decomposition of cyclopamine [24]. We envisioned that its replacement by a methylene group would create an acid-stable cyclopamine analog that still exhibits similar inhibitory activity on hedgehog-signaling. For the sake of brevity of the overall synthetic sequence we defined carbacyclopamine analog **2** (see Figure 1) as our primary target.

## Results and Discussion

**Scheme 1:**
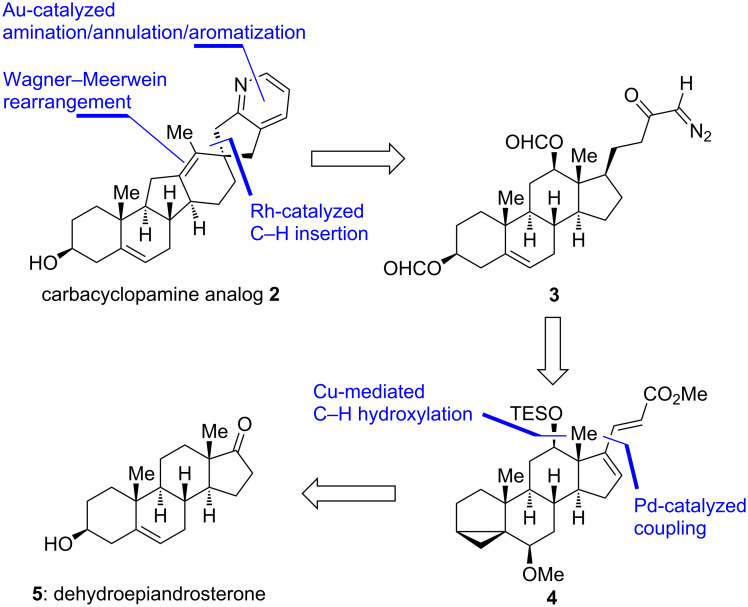
Retrosynthetic analysis of carbacyclopamine analog **2**.

**Scheme 2:**
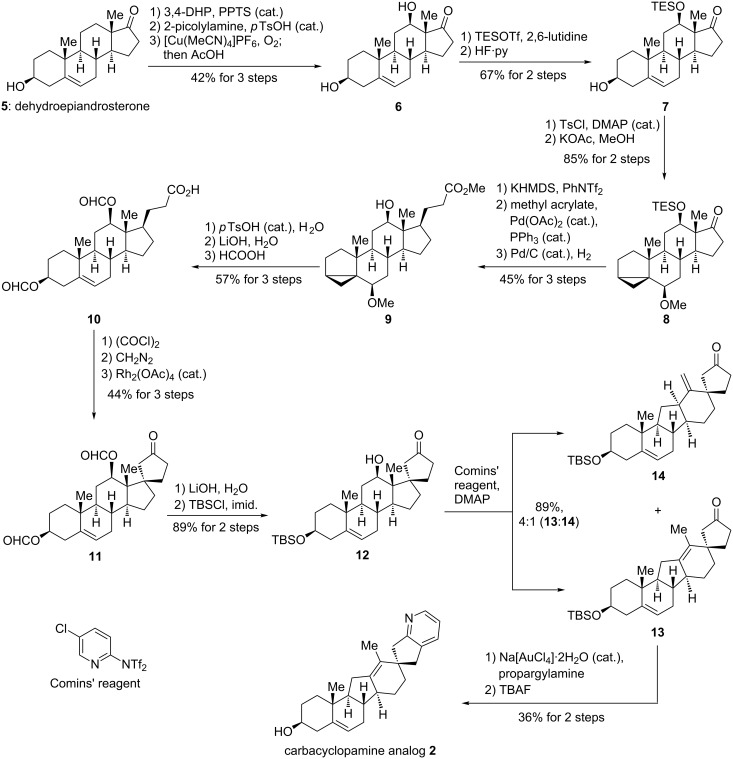
Synthesis of carbacyclopamine analog **2**.

A retrosynthetic analysis identified diazo compound **3** as a key intermediate in the synthesis of **2** (see Scheme 1). We envisioned a rhodium-catalyzed C–H-insertion into the C17–H bond to occur with a high degree of selectivity (both regio- and stereoselectivity) to form the all-carbon E-ring (for its structure see **11**, Scheme 2). Furthermore, a Wagner–Meerwein rearrangement was thought to establish the C-*nor*-D-*homo* steroid system, and a gold-catalyzed amination/annulation/aromatization sequence was planned to install the pyridine F-ring. Diazo compound **3** originates from diene **4** through standard transformations, the latter being accessible from commercially available and inexpensive dehydroepiandrosterone (**5**) by the means of a copper-mediated C–H hydroxylation in the 12-position and a palladium-catalyzed coupling of methyl acrylate to an activated enol ether in the 17-position.

In synthetic direction (Scheme 2), dehydroepiandrosterone (**5**) was protected as its tetrahydropyranyl ether (3,4-dihydro-2*H*-pyran, cat. pyridinium *para*-toluenesulfonate, CH_2_Cl_2_, 25 °C, quantiative, inconsequential mixture of diastereoisomers), and the C17 carbonyl group was transformed into its 2-picolylimine (2-picolylamine, cat. *para*-toluenesulfonic acid, toluene, 111 °C, 92% yield). Subjecting the latter to modified Schönecker conditions ([Cu(MeCN)_4_]PF_6_, O_2_, acetone, 25 °C) [25–27], gave, after acidic work-up (AcOH/MeOH, 1:1, 90 °C), diol **6** in 46% yield as the only isolable product. Protection of the newly installed 12β-hydroxy group as a triethylsilyl ether via the bis-protected intermediate (not shown, triethylsilyl trifluoromethanesulfonate, 2,6-lutidine, CH_2_Cl_2_, 0 °C; then HF·pyridine, THF, 0 °C, 67% yield for the two steps) [28] gave hydroxy ketone **7**.

The remaining homoallylic alcohol was then masked as an *i*-steroid [29–30] (*para*-toluenesulfonyl chloride, cat. 4-dimethylaminopyridine, pyridine, 25 °C; then KOAc, MeOH, 64 °C, 85% yield for the two steps) to give **8**. Generation of an enol triflate from the carbonyl group in **8** (potassium hexamethyldisilazide, phenyl bis-triflimide, THF, −20 → −10 °C, 85% yield) set stage for a Heck-reaction with methyl acrylate (cat. Pd(OAc)_2_, cat. PPh_3_, Et_3_N, DMF, 70 °C, 64% yield). The hydrogenation of the so-obtained diene **4** (for its structure see Scheme 1) proceeded smoothly (H_2_, cat. Pd/C, MeOH, 25 °C, 82% yield) under concomitant removal of the triethylsilyl ether [31] in 12-position to give methyl ester **9** as a single diastereoisomer. At this point, extensive experimentation suggested a change of protecting groups to enable the pending C–H insertion reaction at C17.

Therefore, the *i*-steroid was reverted to the homoallylic alcohol (cat. *para*-toluenesulfonic acid, 1,4-dioxane/H_2_O, 10:1, 65 °C), the methyl ester was hydrolyzed under basic conditions (LiOH, THF/H_2_O, 1:1, 68% yield for the two steps), and the alcohol moieties were protected as formyl esters (formic acid, 50 °C, 85% yield) to give key intermediate **10**.

Employing the formyl protecting groups [32], diazoketone **3** (for its structure see Scheme 1) was readily obtained from acid **10** via the corresponding acid chloride (oxalyl chloride, CH_2_Cl_2_, 25 °C; then diazomethane, THF, 25 °C, 85% yield for the two steps) [33]. The anticipated C–H insertion then proceeded uneventfully under oxygen-free conditions (cat. Rh_2_(OAc)_4_, CH_2_Cl_2_, 41 °C) [34–35] to give cyclopentanone **11** in 52% yield as a single isomer. Removal of the formyl ester protecting groups (LiOH, THF/H_2_O, 1:1, 30 °C, 94% yield) and selective protection of the 3-hydroxy group as a *tert*-butyldimethylsilyl ether (*tert*-butyldimethylsilyl chloride, imidazole, DMF, 25 °C, 95% yield) furnished alcohol **12**.

Treatment of the latter under our previously reported conditions [36] (Comins’ reagent, 4-dimethylaminopyridine, toluene, 111 °C) gave an inseparable mixture of the rearranged olefin products, with the desired *endo*-product **13** as the major constituent (89% combined yield, *endo*-product **13**:*exo*-product **14**, 4:1). Isolation of desired product **13** by chromatography was then achieved after selective hydrogenation of only the exocyclic olefin in **14** (H_2_, cat. Rh/C, EtOAc, 25 °C, structure of hydrogenation product not shown) employing the mixture of the isomers **13** and **14** from the previous step.

Finally, a gold-catalyzed amination/annulation/aromatization sequence (propargylamine, cat. Na[AuCl_4_]·2H_2_O, EtOH, 100 °C) [37–38] furnished, after removal of the *tert*-butyldimethylsilyl ether (tetrabutylammonium fluoride, THF, 25 °C), carbacyclopamine analog **2** in 36% overall yield for the two steps [39].

Carbacyclopamine analog **2** was then tested for its stability towards acid. Therefore, **2** was treated with a mixture of aqueous HCl/THF (pH of approx. 0.3) for 1 h and ^1^H NMR spectra were recorded before and afterwards. While at this pH natural cyclopamine (**1**) was shown to decompose rapidly [12], carbacyclopamine analog **2** showed no signs of decomposition. This result was in full agreement with our initial considerations.

To determine the ability of carbacyclopamine analog **2** to inhibit Gli1-dependent luciferase expression, we employed Shh-LIGHTII cells, a clonal mouse fibroblast cell line which stably incorporates a Gli-dependent firefly luciferase reporter and a constitutive *Renilla* luciferase reporter [18]. The analog was tested in a concentration range from 0.01 μM to 100 μM but showed no activity (data not shown).

## Conclusion

We have reported the synthesis of a new, acid stable cyclopamine analog. The implementation of C–H-functionalization logic in the synthetic planning and further metal-catalyzed transformations allows for a fast access to carbocyclopamine analog **2**. At the same time, these investigations strongly suggest the role the ether oxygen plays in the acid-catalyzed decomposition of cyclopamine. This study may find further application in the rational design of new hedgehog inhibitors based on lead structure **2**. Future work will focus on the synthesis of carbacyclopamine analogs with a piperidine F-ring and their biological investigation.

## Supporting Information

File 1 Experimental procedures, characterization data, and copies of ^1^H and ^13^C NMR spectra for new compounds.
