# Supplementary material for: C–H-Functionalization logic guides the synthesis of a carbacyclopamine analog
Source: Beilstein J Org Chem. 2014 Jul 9;10:1564–9. doi: 10.3762/bjoc.10.161 (PMC4142839; doi:10.3762/bjoc.10.161)
Supplement: File 1 — Experimental procedures, characterization data, and copies of 1H and 13C NMR spectra for new compounds. [file Beilstein_J_Org_Chem-10-1564-s001.pdf]

Supporting Information

for

C–H-Functionalization logic guides the synthesis of a carbacyclopamine analog

Sebastian Rabe[1], Johann Moschner[1], Marina Bantzi[1], Philipp Heretsch*,[2] and Athanassios Giannis*,[1]

Address: [1]Universität Leipzig, Institut für Organische Chemie, Johannisallee 29, D-04103 Leipzig, Germany and [2]Rice University, Department of Chemistry, BioScience Research Collaborative, 6100 Main St., Houston, Texas 77005, United States

*Corresponding author

Email: Philipp Heretsch - heretsch@rice.edu; Anthanassios Giannis - giannis@uni-leipzig.de

Experimental procedures, characterization data, and copies of ^{1}H and ^{13}C NMR spectra for new compounds

Table of Contents

1. GENERAL INFORMATION S2

2. SYNTHETIC ROUTE S4

3. EXPERIMENTAL PROCEDURES........ S5

3.1 3β-*O*-TETRAHYDROPYRANYLANDROST-5-ENE-17-ONE (15): S5

3.2 17-(*N*-2-PYRIDYLMETHYL)IMINO-3β-*O*-TETRAHYDROPYRANYL-ANDROST-5-ENE (16): S5

3.3 (–)-3β,12β-DIHYDROXYANDROST-5-ENE-17-ONE (6):........ S6

3.4 (–)-3β,12β-BIS(TRIETHYLSILYLOXY)-ANDROST-5-ENE-17-ONE (17) :........ S6

3.5 (–)-3β-HYDROXY-12β-(TRIETHYLSILYLOXY)ANDROST-5-ENE-17-ONE (7):........ S7

3.6 (–)-3β-TOSYLOXY-12β-(TRIETHYLSILYLOXY)ANDROST-5-ENE-17-ONE (18): S8

3.7 (+)-3β,5-CYCLO-5β-6β-METHOXY-12β-TRIETHYLSILYLOXY-ANDROSTANE-17-ONE (8):........ S8

3.8 (+)-3β,5-CYCLO-5β-6β-METHOXY-12β-TRIETHYLSILYLOXY-17-TRIFLUOROMETHANESULFONYLOXYANDROST-16-ENE (19):........ S9

3.9 (–)-METHYL-3β,5-CYCLO-5β-6β-METHOXY-12β-TRIETHYLSILYLOXYPREGNA-16,20-DIENE-21-CARBOXYLATE (4): . S9

3.10 (+)-METHYL-3β,5-CYCLO-5β-12β-HYDROXY-6β-METHOXYPREGNAN-21-CARBOXYLATE (9): S10

3.11 (–)-METHYL-3β,12β-DIHYDROXYPREGN-5-ENE-21-CARBOXYLATE (20): S11

3.12 (–)-3β,12β-DIHYDROXYPREGN-5-ENE-21-CARBOXYLIC ACID (21): S11

3.13 (–)-3β,12β-DIFORMYLOXYPREGN-5-ENE-21-CARBOXYLIC ACID (10): S12

3.14 (–)-3β-ACETOXYPREGN-5-ENE-21,12β-CARBOLACTONE (**25**): S12

3.15 (–)-21-*NOR*-24-DIAZO-3β,12β-DIFORMYLOXY-CHOL-5-ENE-23-ONE (3): S13

3.16 (–)-21,26,27-TRI-NOR-24-DIAZO-3β,12β-DIFORMYLOXYCHOLEST-5-ENE-23-ONE (24) S13

3.17 (–)-(17*R*)-SPIRO[3β,12β-DIFORMYLOXY-ANDROST-5-ENE-17,3'-CYCLOPENTA-1'-ONE] (11): S14

3.18 (–)-(17*R*)-SPIRO[3β,12β-DIHYDROXY-ANDROST-5-ENE-17,3'-CYCLOPENTA-1'-ONE] (22): S15

3.19 (–)-(17*R*)-SPIRO[3β-*TERT*-BUTYLDIMETHYLSILYLOXY-12β-HYDROXYANDROST-5-ENE-17,3'-CYCLOPENTA-1'-ONE] (12): S15

3.20 (+)-(17*R*)-SPIRO[14(13→12)ABEO-3β-*TERT*-BUTYLDIMETHYLSILYLOXY-ANDROSTA-5,12-DIEN-17,3'-CYCLOPENTA-1'-ONE] (13): S16

3.21 (–)-(17*R*)-SPIRO[14(13→12)ABEO-3β-*TERT*-BUTYLDIMETHYLSILYLOXY-ANDROSTA-5,12-DIEN-17,6'-PYRINDAN] (23): S17

3.22 CARBACYCLOPAMINE ANALOG 2: S17

4. ACID STABILITY S18

5. BIOCHEMISTRY S18

5.1 LUCIFERASE REPORTER ASSAY S19

6. NMR SPECTRA S21

7. REFERENCES S44

1. General Information

General procedures. All reactions were run under an atmosphere of argon unless otherwise indicated. Room temperature refers to 22 °C, ambient pressure to 1013 hPa.

Reagents and anhydrous solvents were transferred via oven-dried syringes and cannulae. Flasks were flame-dried under vacuum and cooled under a constant stream of argon.

Tetrahydrofuran was distilled from potassium under argon, dichloromethane from SICAPENT (phosphorus pentoxide on solid support with indicator), ethanol from magnesium ethoxide and triethylamine from calcium hydride. Methanol, toluene, acetone, dimethylformamide,

acetonitrile and pyridine were purchased from Acros or Sigma-Aldrich (anhydrous over molecular sieves).

All other chemicals were purchased from ABCR, Acros, Sigma-Aldrich, Alfa Aesar, Fluorochem, Merck and TCI Europe at the highest commercially available purity and used as such.

Reactions were monitored by thin-layer chromatography using Merck silica gel 60 F_{254} TLC aluminium sheets and visualized with ceric ammonium molybdate, potassium permanganate or vanillin staining solution. Chromatographic purification was performed as flash chromatography on Merck silica gel 40–63 µm, 60 Å, using a forced flow of eluent (method of Still). Concentration under reduced pressure was performed by rotary evaporation at 40 °C at the appropriate pressure.

Yields refer to chromatographically purified and spectroscopically pure compounds.

NMR spectra were recorded on a Bruker Avance 700 (operating at 700 MHz for ^{1}H and 176 MHz for ^{13}C), Varian Mercury plus 400 (operating at 400 MHz for ^{1}H and 100 MHz for ^{13}C) and a Varian Mercury plus 300 (operating at 300 MHz for ^{1}H and 75 MHz for ^{13}C acquisitions). Chemical shifts δ are reported in ppm with the solvent resonance as the internal standard (d_1-chloroform: 7.26 (^{1}H-NMR), 77.16 (^{13}C-NMR); d_6-benzene: 7.16 (^{1}H-NMR), 128.06 (^{13}C-NMR); d_4-methanol: 3.31 (^{1}H-NMR), 49.00 (^{13}C-NMR). Coupling constants *J* are reported in Hertz (Hz). Multiplicities are classified by the following abbreviations: s = singlet, d = doublet, t = triplet, q = quartet, p = quintet, and combinations thereof, or m = multiplet, or br = broad signal.

Where 2D-spectra were recorded and allowed complete assignment of all hydrogen and carbon-atoms of a compound, spectral data include this assignment using common steroid numbering. Where this is not the case, all hydrogen signals below 2 ppm are omitted and only methyl groups and isolated signals in this range are listed. All spectra can be found as copies at the end of the experimental section.

High resolution mass spectra were obtained on a Bruker Daltonics ESI-FT-ICR-MS APEX II.

IR spectra were obtained on an ATI/MATTSON Genesis FT-IR and JASCO FT/IR-4100typeA as thin film (in CCl_4) or KBr disk. Absorbance frequencies are reported in reciprocal centimeters (cm^{-1}).

Melting points were measured on a Boetius-micro hot stage and are uncorrected.

Optical rotations were obtained on a Schmidt+Haensch Polartronic MHZ-8 at the sodium-D line (589 nm) using a 50 mm path-length cell and solvent and concentration as indicated.

2. Synthetic Route

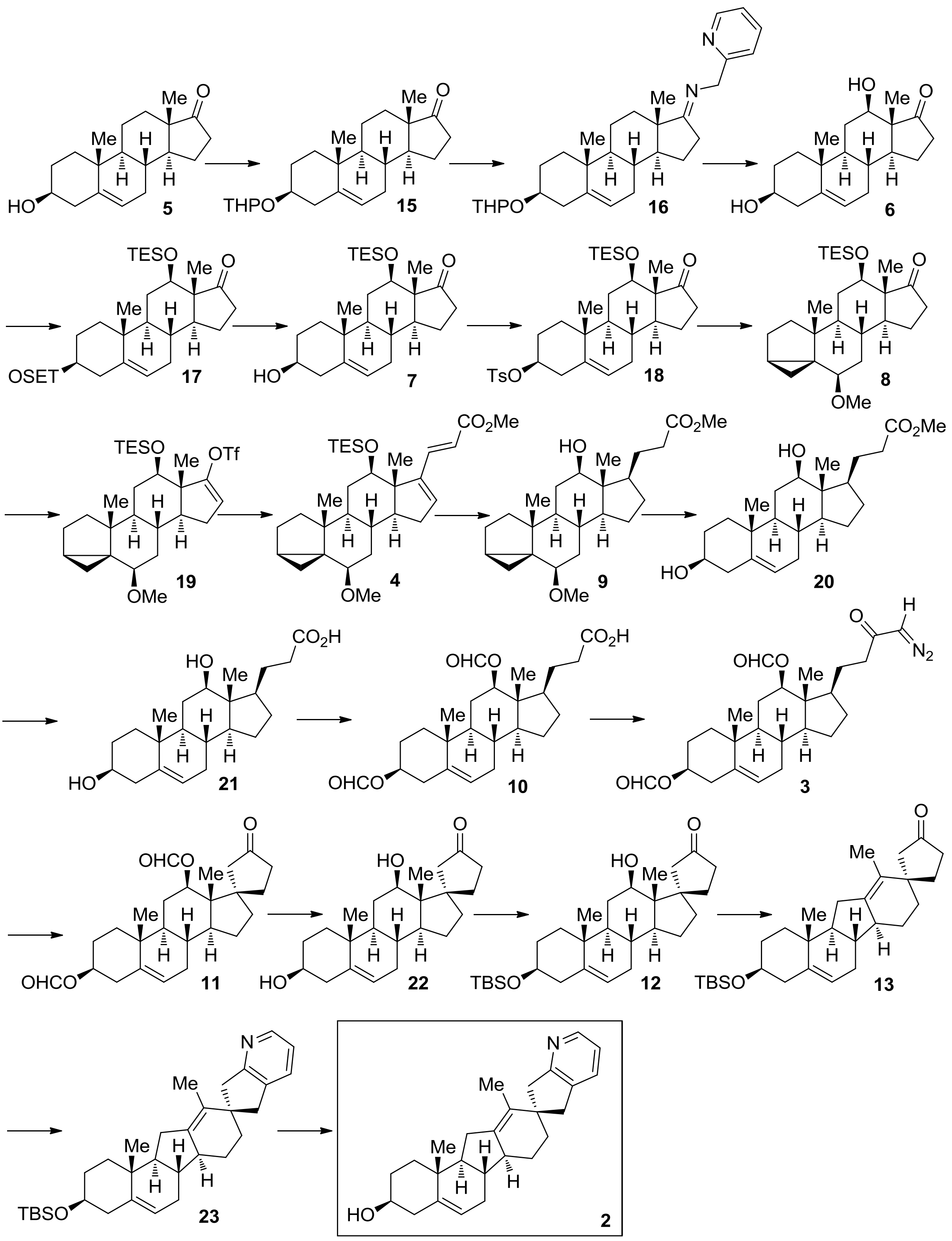

3. Experimental Procedures

3.1 3β-*O*-Tetrahydropyranylandrost-5-ene-17-one (15):

To a solution of dehydroepiandrosterone (**5**) (80.0 g, 277.0 mmol, 1.0 equiv.) in CH_2Cl_2 (1.1 L) at room temperature was sequentially added 3,4-dihydro-2*H*-pyran (55.0 mL, 604.0 mmol, 2.7 equiv.) and pyridinium *para*-toluenesulfonate (3.48 g, 13.9 mmol, 0.05 equiv.). After stirring for 2.5 h the mixture was quenched with water (400 mL). The phases were separated, the aqueous layer was extracted with CH_2Cl_2 (2 x 300 mL) and the combined organic phases were dried ($MgSO_4$). All volatiles were removed under reduced pressure to give crude title compound (84.5 g, 227.0 mmol, quant.; mixture of diastereoisomers) as a colorless solid which was used in the next step without further purification.

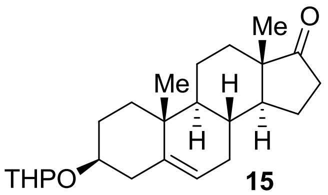

15

15: mp.: 166-169 °C; R_f: 0.33 (*n*-hexane/EtOAc, 5:1); IR (KBr): ν_{max} 2940, 1731, 1112, 1057, 1028, 975 cm^{-1}; ^{1}H-NMR (400 MHz, $CDCl_3$) δ 5.37 (m, 1 H), 4.71 (m, 1 H), 3.90 (m, 1 H), 3.52 (m, 1 H), 3.49 (m, 1 H), 2.45 (m, 1 H), 2.37 (m, 1 H), 1.03 (s, 3 H), 0.87 (s, 3 H); ^{13}C-NMR (100 MHz, $CDCl_3$) δ 221.1, 170.6, 140.1, 122.0, 73.9, 51.8, 50.3, 47.7, 38.2, 37.1, 36.9, 35.9, 31.6, 31.5, 30.9, 27.8, 22.0, 21.6, 20.5, 19.5, 13.7; HRMS (m/z): $[M+Na]^+$ calculated for $C_{24}H_{36}O_3Na$: 395.25567, found: 395.25532; calculated for $C_{48}H_{72}O_6Na$: 767.52211, found: 767.52123.

3.2 17-(*N*-2-Pyridylmethyl)imino-3β-*O*-tetrahydropyranyl-androst-5-ene (16):

To a solution of **15** (26.3 g, 70.7 mmol, 1.0 equiv.) in toluene (600 mL) was sequentially added *para*-toluenesulfonic acid monohydrate (336 mg, 1.80 mmol, 0.025 equiv.) and 2-picolylamine (38.2 g, 353.5 mmol, 5.0 equiv.). The mixture was heated to 110 °C for 3.5 h with a Dean-Stark trap and then allowed to cool to room temperature. The reaction mixture was diluted with EtOAc (950 mL) and washed sequentially with saturated aqueous $NaHCO_3$-solution (2 x 500 mL) and saturated brine (500 mL). The combined organic phases were dried (Na_2SO_4), the crude was concentrated under reduced pressure and the residue was recrystallized from a mixture of boiling EtOAc:Et_2O (2:1, 620 mL) to give pure title compound (30.1 g, 65.1 mmol, 92%, mixture of diastereoisomers) as colorless needles.

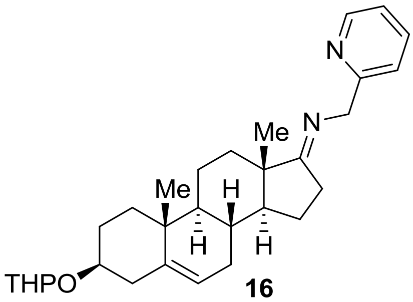

16

16: mp.: 158-160 °C; IR (KBr): ν_{max} 2944, 2849, 1672, 1591, 1438, 1136, 1074, 1031, 765 cm^{-1}; ^{1}H-NMR (300 MHz, $CDCl_3$) δ 8.52 (m, 1 H), 7.65 (m, 1 H), 7.42 (m, 1 H), 7.12 (m, 1 H) 5.37 (m, 1 H), 4.72 (m, 1 H), 4.63 (d, *J* = 16.5 Hz, 1 H), 4.55 (d, *J* = 16.5 Hz, 1 H), 3.91 (m, 1

H), 3.49 (m, 2 H), 2.44 (m, 2 H), 2.34 (m, 1 H), 1.04 (s, 1 H), 0.91 (s, 1 H); ^{13}C-NMR (75 MHz, $CDCl_3$) δ 186.0, 160.6, 149.0, 141.5, 141.3, 121.6, 121.1, 97.0, 76.0, 63.0, 58.1, 53.4, 50.7, 45.7, 40.4, 38.9, 37.5, 37.3, 37.1, 34.2, 31.7, 31.4, 29.8, 28.1, 25.6, 23.4, 20.8, 20.1, 19.5, 16.3; HRMS (m/z): $[M+H]^+$ calculated for $C_{30}H_{43}N_2O_2$: 463.33191, found: 463.33132; calculated for $C_{60}H_{84}N_4O_4Na$: 947.63848, found: 947.63743.

<u>3.3 (–)-3β,12β-Dihydroxyandrost-5-ene-17-one (**6**):</u>

To a suspension of imine **16** (30.8 g, 66.7 mmol, 1.0 equiv.) in acetone (600 mL) was added copper(I) tetra(acetonitrilo) hexafluorophosphate (29.8 g, 80.1 mmol, 1.2 equiv.) in one portion. The brownish suspension was stirred for 1 h after which dry oxygen was bubbled through the suspension for 30 min and the resulting dark-green solution was stirred under an atmosphere of oxygen for 24 h. The solvent was removed under reduced pressure, the resulting green solid was taken up in EtOAc/Et_2O/NH_3 (aq, 25%) (2.0 L, 1:2:1), the layers were separated and the organic layer was washed with aqueous ammonia (25%, 2 x 300 mL) and saturated brine (150 mL), dried (Na_2SO_4) and the solvents were removed under reduced pressure. The residue was redissolved in MeOH/AcOH (600 mL, 1:1) and heated to 90°C for 6 h. The solvents were removed under reduced pressure and the residue was partitioned between EtOAc (1.3 L) and saturated brine (200 mL). The organic phase was dried ($MgSO_4$) and concentrated under reduced pressure. The crude product was purified by silica gel chromatography (SiO_2, *n*-hexane/EtOAc, 1:2, *v*/*v*) to give pure title compound (9.36 g, 30.7 mmol, 46%) as a yellowish solid.

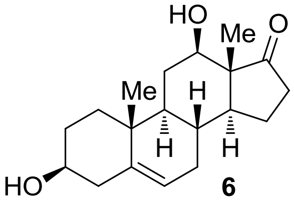

6

6: mp.: 190-194 °C; R_f: 0.38 (*n*-hexane/EtOAc, 1:2); $[\alpha]_D^{22}$: –22.3 (deg cm^3 g^{-1} dm^{-1}, c = 0.99, $CHCl_3$); IR (KBr): ν_{max} 3429, 2931, 1730, 1466, 1437, 1383, 1047, 846 cm^{-1}; ^{1}H-NMR (400 MHz, MeOH-d_4) δ 5.39 (m, 1 H), 3.74 (dd, *J* = 11.2, 4.8 Hz, 1 H), 3.40 (m, 1 H), 2.44 (m, 1 H), 1.06 (s, 3 H), 0.94 (s, 3 H); ^{13}C-NMR (100 MHz, MeOH-d_4) δ 223.0, 142.3, 121.9, 73.1, 72.2, 52.8, 51.0, 50.6, 42.9, 38.4, 37.8, 36.5, 32.2, 31.7, 31.4, 30.4, 22.4, 19.8, 8.4; HRMS (m/z): $[M+Na]^+$ calculated for $C_{19}H_{28}O_3Na$: 327.19307, found: 327.19359; $[2M+Na]^+$ calculated for $C_{38}H_{56}O_6Na$: 631.39691, found: 631.39730.

<u>3.4 (–)-3β,12β-bis(Triethylsilyloxy)-androst-5-ene-17-one (**17**) :</u>

To a solution of diol **6** (6.00 g, 19.7 mmol, 1.0 equiv.) and 2,6-lutidine (9.2 mL, 78.8 mmol, 4.0 equiv.) in CH_2Cl_2 (800 mL) at 0 °C was added dropwise triethylsilyl trifluoromethanesulfonate (9.8 mL, 43.4 mmol, 2.2 equiv.) over a period of 45 min. After 3 h at the same temperature another portion of triethylsilyl trifluoromethanesulfonate (2.3 mL, 10.0

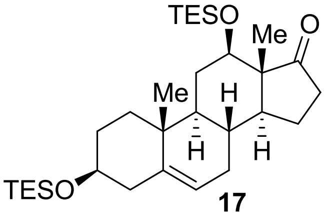

17

mmol, 0.5 equiv.) was added and the mixture was stirred for an additional 1 h. The reaction was then quenched with saturated aqueous $NaHCO_3$-solution (200 mL), the phases were separated and the aqueous phase was extracted with CH_2Cl_2 (2 x 200 mL). The combined organic phases were dried ($MgSO_4$), all volatiles were removed under reduced pressure and the crude product was purified by column chromatography (SiO_2, *n*-hexane/EtOAc; 20:1, *v*/*v*) to give pure title compound (8.30 g, 15.6 mmol, 79%) as a colorless oil.

17: R_f: 0.63 (*n*-hexane/EtOAc, 10:1); $[\alpha]_D^{22}$: –17.3 (deg cm^3 g^{-1} dm^{-1}, c = 1.05, $CHCl_3$); IR (KBr): ν_{max} 2953, 2875,1744, 1459, 1093, 1012, 825, 741 cm^{-1}; ^1H-NMR (300 MHz, $CDCl_3$) δ 5.34 (m, 1 H), 3.72 (dd, *J* = 10.8, 4.9 Hz, 1 H), 3.47 (m, 1 H), 2.41 (m, 1 H), 1.02 (s, 3 H), 0.96 (s, 3 H); ^{13}C-NMR (75 MHz, $CDCl_3$) δ 218.2, 141.8, 120.6, 72.3, 72.2, 52.0, 50.4, 49.4, 42.9, 37.5, 36.8, 35.8, 32.2, 31.7, 30.6, 30.5, 21.2, 19.5, 8.4, 7.2, 7.0, 6.9, 6.6, 5.7, 5.4, 5.0; HRMS (m/z): $[M+Na]^+$ calculated for $C_{31}H_{56}O_3Si_2Na$: 555.36602, found: 555.36555; $[2M+Na]^+$ calculated for $C_{62}H_{112}O_6Si_4Na$: 1087.74282, found: 1087.74281.

3.5 (–)-3β-Hydroxy-12β-(triethylsilyloxy)androst-5-ene-17-one (**7**):

To a solution of HF•pyridine complex (70% HF, 30% pyridine, 3.6 mL) in pyridine (22 mL) and THF (110 mL) at 0 °C was added dropwise a solution of *bis*-silylether **17** (8.62 g; 16.2 mmol, 1.0 equiv.) in THF (20 mL). After 2 h at the same temperature the reaction mixture was diluted with Et_2O (400 mL) and neutralized with saturated $NaHCO_3$-solution (200 mL). The phases were separated and the aqueous layer was extracted with Et_2O (3 x 300 mL). The combined organic layers were dried ($MgSO_4$), all volatiles were removed under reduced pressure and the crude product was purified by column chromatography (SiO_2, *n*-hexane/EtOAc; 3:1, *v*/*v*) to give pure title compound (5.64 g, 13.8 mmol, 85%) as a colorless foam.

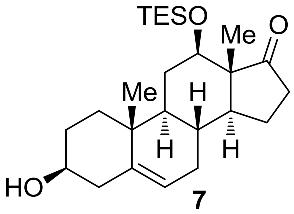

7

7: R_f: 0.30 (*n*-hexane/EtOAc, 3:1); $[\alpha]_D^{22}$: –40.9 (deg cm^3 g^{-1} dm^{-1}, c = 0.63, $CHCl_3$); IR (KBr): ν_{max} 3435, 2955, 2875, 1742, 1458, 1240, 1102, 1054, 825, 742 cm^{-1}; ^1H-NMR (400 MHz, $CDCl_3$) δ 5.36 (m, 1 H), 3.72 (dd, *J* = 10.8, 4.9 Hz, 1 H), 3.51 (m, 1 H), 2.41 (m, 1 H), 2.31 (m, 1 H), 2.23 (m, 1 H), 1.02 (s, 3 H), 0.96 (s, 3 H); ^{13}C-NMR (100 MHz, $CDCl_3$) δ 218.3, 141.0, 121.0, 72.1, 71.7, 51.9, 50.4, 49.2, 42.3, 37.3, 36.7, 35.8, 31.7, 31.7, 30.6, 30.5, 21.2, 19.5, 8.4, 7.1, 5.3; HRMS (m/z): $[M+Na]^+$ calculated for $C_{25}H_{42}O_3SiNa$: 441.27954, found: 441.27933; $[2M+Na]^+$ calculated for $C_{50}H_{84}O_6Si_2Na$: 859.56986, found: 859.56940.

3.6 (–)-3β-Tosyloxy-12β-(triethylsilyloxy)androst-5-ene-17-one (**18**):

To a solution of the alcohol **7** (5.70 g, 13.6 mmol, 1.0 equiv.) in pyridine (50 mL) at 0 °C was added *para*-toluenesulfonyl chloride (6.40 g, 34.0 mmol, 2.5 equiv.) and the reaction mixture

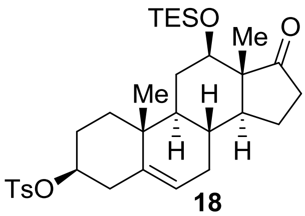

18

was stirred for 16 h at room temperature. The mixture was then cooled to 0 °C and quenched with water (25 mL). The reaction mixture was extracted with EtOAc (3 x 60 mL), the combined organic layers were washed with saturated brine (50 mL) and dried ($MgSO_4$). All volatiles were removed under reduced pressure and the crude product was purified by column chromatography (SiO_2, *n*-hexane/EtOAc; 10:1, *v*/*v*) to give pure title compound (7.32 g, 12.8 mmol, 94%) as a colorless foam.

18: R_f: 0.20 (*n*-hexane/EtOAc, 10:1); $[\alpha]_D^{22}$: –29.3 (deg cm^3 g^{-1} dm^{-1}, c = 0.90, $CHCl_3$); IR (KBr): ν_{max} 2953, 1741, 1364, 1176, 1095, 940, 865, 739 cm^{-1}; ^{1}H-NMR (400 MHz, $CDCl_3$) δ 7.79 (d, *J* = 8.2 Hz, 2 H), 7.33 (d, *J* = 8.2 Hz, 2 H), 5.34 (m, 1 H), 4.32 (m, 1 H), 3.70 (dd, *J* = 10.8, 4.8 Hz, 1 H), 2.44 (s, 3 H), 2.36 (m, 3 H), 0,95 (s, 3 H), 0.91 (s, 3 H); ^{13}C-NMR (100 MHz, $CDCl_3$) δ 218.0, 144.6, 139.1, 134.8, 129.9, 127.8, 122.9, 82.0, 72.0, 51.9, 50.2, 49.0, 38.9, 36.9, 36.5, 35.7, 31.6, 30.5, 30.4, 28.6, 21.8, 21.1, 19.2, 8.4, 7.1, 5.3; HRMS (m/z): $[M+Na]^+$ calculated for $C_{32}H_{48}O_5SSiNa$: 595.28839, found: 595.28787; $[2M+Na]^+$ calculated for $C_{64}H_{96}O_{10}S_2Si_2Na$: 1167.58765, found: 1167.58798.

3.7 (+)-3β,5-Cyclo-5β-6β-methoxy-12*β*-triethylsilyloxy-androstane-17-one (**8**):

A flask containing potassium acetate (8.50 g, 86.8 mmol, 7.0 equiv.) was dried under vacuum (0.7 mbar) at 120 °C for 24 h. It was allowed to cool to room temperature and a

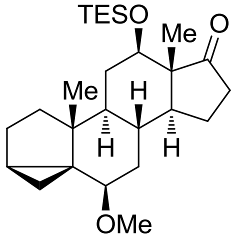

8

solution of tosylate **18** (7.10 g, 12.4 mmol, 1.0 equiv.) in MeOH (230 mL) was added. The reaction mixture was heated to 64 °C for 1.5 h. The mixture was allowed to cool to room temperature, water (100 mL) was added and the mixture was extracted with EtOAc (3 x 500 mL). The combined organic layers were washed with saturated brine (100 mL), dried ($MgSO_4$) and concentrated under reduced pressure The so-obtained crude product was purified by column chromatography (SiO_2, *n*-hexane/EtOAc; 20:1, *v*/*v*) to give pure title compound (7.83 g, 11.2 mmol, 90%) as a colorless foam.

8: R_f: 0.28 (*n*-hexane/EtOAc, 10:1), $[\alpha]_D^{22}$: +54.4 (deg cm^3 g^{-1} dm^{-1}, c = 1.60, $CHCl_3$); IR (KBr): ν_{max} 2953, 2874,1743, 1457, 1092, 1016, 853, 741 cm^{-1}; ^{1}H-NMR (400 MHz, $CDCl_3$) δ 3.77 (dd, *J* = 11.2, 4.8 Hz, 1 H), 3.34 (s, 3 H) 2.81 (m, 1 H) 2.41 (m, 1 H), 1.03 (s, 3 H), 0.96 (s, 3 H), 0.48 (dd, *J* = 7.9, 5.3 Hz, 1 H); ^{13}C-NMR (75 MHz, $CDCl_3$) δ 218.3, 82.0, 72.6, 56.8, 52.5, 50.1, 46.6, 43.5, 35.8, 35.0, 33.9, 33.5, 33.1, 29.4, 25.0, 21.3, 21.0, 19.3, 13.3, 8.7, 7.1, 5.3; HRMS (m/z): $[M+Na]^+$ calculated for $C_{26}H_{44}O_3SiNa$: 455.29519, found: 455.29541; $[2M+Na]^+$ calculated for $C_{52}H_{88}O_6Si_2Na$: 887.60116, found: 887.60202.

3.8 (+)-3β,5-Cyclo-5β-6β-methoxy-12β-triethylsilyloxy-17-trifluoromethane-sulfonyl oxyandrost-16-ene (**19**):

To a solution of *i*-steroid **8** (10.30 g, 23.9 mmol, 1.0 equiv.) in THF (250 mL) at –20 °C was added potassium hexamethyldisilazide (0.5 M in toluene, 134.0 mL, 67.0 mmol, 2.8 equiv.) and the reaction mixture was stirred for 1 h. The cooling bath was removed and the mixture was stirred for 15 min while warming to room temperature. Afterwards, the reaction mixture was cooled to –10 °C and a solution of *N*-phenyl-*bis*-(trifluoromethane sulfonimide) (14.50 g, 40.6 mmol, 1.7 equiv.) in THF (50 mL) was added dropwise. The reaction mixture was stirred for 20 min at –10 °C at which time it was partitioned between water (50 mL) and EtOAc (250 mL). The aqueous layer was separated and extracted with EtOAc (3 x 300 mL), the combined organic layers were washed with saturated brine (250 mL), dried ($MgSO_4$) and all volatiles were removed under reduced pressure. The so-obtained crude product was purified by column chromatography (SiO_2, *n*-hexane/EtOAc; 30:1, *v*/*v*) to give pure title compound (11.50 g, 20.3 mmol, 85%) as a colorless solid.

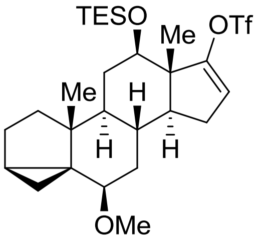

19

19: mp.: 125-128 °C; R_f: 0.48 (*n*-hexane/EtOAc, 10:1); $[\alpha]_D^{22}$: +21.7 (deg cm^3 g^{-1} dm^{-1}, c = 0.90, $CHCl_3$); IR (KBr): ν_{max} 2956, 1626, 1423, 1212, 1144, 1090, 894, 816, 733 cm^{-1}; ^{1}H-NMR (400 MHz, C_6D_6) δ 5.34 (m, 1 H), 3.85 (m, 1 H), 3.13 (s, 3 H) 2.49 (m, 1 H) 1.88 (m, 1 H), 0.52 (m, 1 H), 0.33 (dd, J = 8.0, 5.2 Hz, 1 H); ^{13}C-NMR (75 MHz, C_6D_6) δ 159.6, 115.0, 82.0, 75.1, 56.7, 51.8, 50.8, 47.4, 43.8, 35.4, 34.1, 33.5, 32.9, 27.9, 27.8, 25.1, 21.3, 19.3, 13.4, 11.3, 7.3, 6.0; ^{19}F-NMR (376 MHz, C_6D_6) δ –73.8; HRMS (m/z): $[M+Na]^+$ calculated for $C_{27}H_{43}F_3O_5SSiNa$: 587.24448, found: 587.24411.

3.9 (–)-Methyl 3β,5-cyclo-5β-6β-methoxy-12*β*-triethylsilyloxypregna-16,20-diene-21-carboxylate (**4**):

To a stirred solution of enoltriflate **19** (3.70 g, 6.56 mmol, 1.0 equiv.) in DMF (75 mL) at room temperature was added triethylamine (2.8 mL, 19.7 mmol, 3.0 equiv.), methyl acrylate (1.50 mL, 16.4 mmol, 2.5 equiv.), triphenylphosphine (172 mg, 0.66 mmol, 0.1 equiv.) and palladium(II) acetate (74 mg, 0.33 mmol, 0.05 equiv.). The reaction mixture was heated to 70 °C for 75 min and then allowed to cool to room temperature. It was partitioned between water (25 mL) and EtOAc (150 mL) and the aqueous layer was extracted with EtOAc (100 mL). The combined organic layers were washed with saturated brine,

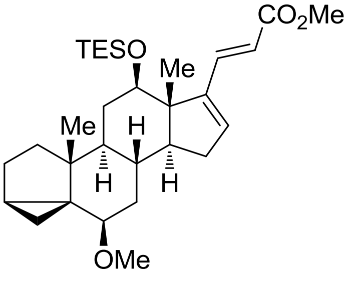

4

dried ($MgSO_4$) and all volatiles were removed under reduced pressure. The so-obtained crude product was purified by column chromatography (SiO_2, *n*-hexane/EtOAc; 20:1, *v*/*v*) to give pure title compound (2.10 g, 4.20 mmol, 64%) as a colorless oil.

4: R_f: 0.25 (*n*-hexane/EtOAc, 10:1); $[\alpha]_D^{22}$: –29.8 (deg cm^3 g^{-1} dm^{-1}, c = 0.93, $CHCl_3$); IR (CCl_4): ν_{max} 2953, 2875, 1651, 1084, 1016, 788, 743 cm^{-1}; ^{1}H-NMR (300 MHz, $CDCl_3$) δ 7.51 (d, *J* = 16.0 Hz, 1 H), 6.18 (d, *J* = 16.0 Hz, 1 H), 6.10 (m, 1 H), 3.73 (s, 3 H), 3.72 (m, 1 H), 3.34 (s, 3 H) 2.79 (m, 1 H), 2.21 (ddd, *J* = 16.6, 7.0, 3.2 Hz, 1 H), 1.05 (s, 3 H), 0.95 (s, 3 H), 0.48 (dd, J = 8.0, 5.3 Hz, 1 H); ^{13}C-NMR (75 MHz, $CDCl_3$) δ 167.7, 151.8, 141.3, 132.6, 118.0, 82.3, 76.6, 56.8, 54.2, 52.2, 51.4, 47.5, 43.7, 35.3, 35.0, 33.3, 32.9, 28.2, 25.0, 21.4, 19.2, 13.2, 12.4, 7.1, 5.6; HRMS (m/z): $[M+Na]^+$ calculated for $C_{30}H_{48}O_4SiNa$: 523.32141, found: 523.32145; $[2M+Na]^+$ calculated for $C_{60}H_{96}O_8Si_2Na$: 1023.65359, found: 1023.65675.

3.10 (+)-Methyl 3β,5-cyclo-5β-12β-hydroxy-6β-methoxypregnan-21-carboxylate (**9**):

To a stirred solution of diene **4** (2.09 g, 4.17 mmol, 1.0 equiv.) in MeOH (100 mL) was added palladium on charcoal (10%, 340 mg, 0.32 mmol, 0.08 equiv.). The reaction mixture was flushed five times with H_2 and stirred under an H_2-atmosphere for 2 h. The suspension was filtered through Celite and concentrated under reduced pressure. The so-obtained crude product was purified by column chromatography (SiO_2, *n*-hexane/EtOAc; 5:1, *v*/*v*) to give pure title compound (1.34 g, 3.42 mmol, 82%) as a colorless oil.

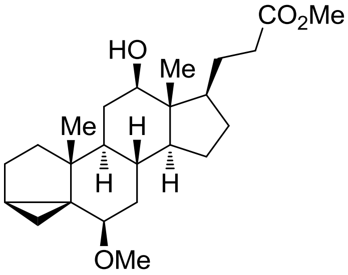

9

9: R_f: 0.63 (*n*-hexane/EtOAc, 2:1); $[\alpha]_D^{22}$: +50.4 (deg cm^3 g^{-1} dm^{-1}, c = 1.00, $CHCl_3$); IR (CCl_4): ν_{max} 3470, 2950, 2870, 1737, 1083, 787, 713 cm^{-1}; ^{1}H-NMR (300 MHz, $CDCl_3$) δ 3.73 (s, 3 H, -$COOCH_3$), 3.41 (dd, *J* = 11.2, 4.5 Hz, 1 H, H-12), 3.31 (s, 3 H, -OCH_3), 2.76 (m, 1 H, H-6), 2.33 (m, 1 H, H-21), 2.30 (m, 1 H, H-21), 1.91 (m, 1 H, H-7), 1.86 (m, 1 H, H-16), 1.71 (m, 1 H, H-2), 1.69 (m, 1 H, H-8), 1.65 (m, 1 H, H-15), 1.57 (m, 1 H, H-11), 1.52 (m, 1 H, H-2), 1.50 (m, 1 H, H-1), 1.43 (m, 2 H, H-20), 1.43 (m, 1 H, H-17), 1.41 (m, 1 H, H-11), 1.28 (m, 1 H, H-15), 1.22 (m, 1 H, H-16), 1.03 (s, 3 H, H-19), 1.02 (m, 1 H, H-7), 0.94 (m, 1 H, H-14), 0.87 (m, 1 H, H-3), 0.86 (m, 1 H, H-1), 0.71 (s, 3 H, H-18), 0.65 (m, 1 H, H-4), 0.44 (dd, J = 8.0, 5.2 Hz, 1 H, H-4); ^{13}C-NMR (100 MHz, $CDCl_3$) δ 175.1 (C-21), 82.4 (C-6), 80.2 (C-12), 56.7 (-OCH_3), 54.4 (C-14), 51.7 (-$COOCH_3$), 50.6 (C-17), 47.4 (C-13), 47.1 (C-9), 43.5 (C-10), 35.3 (C-5), 35.0 (C-7), 33.6 (C-21), 33.4 (C-1), 32.3 (C-11), 29.6 (C-8), 29.0 (C-16), 27.7 (C-20), 25.0 (C-2), 24.5 (C-15), 21.5 (C-3), 19.3 (C-19), 13.2 (C-4), 7.4 (C-18); HRMS (m/z): $[M+Na]^+$ calculated for $C_{24}H_{38}O_4Na$: 413.26623, found: 413.26650; $[2M+Na]^+$ calculated for $C_{48}H_{76}O_8Na$: 803.54324, found: 803.53868.

3.11 (–)-Methyl 3β,12β-dihydroxypregn-5-ene-21-carboxylate (**20**):

To a solution of methylester **9** (1.33 g, 3.40 mmol, 1.0 equiv.) in 1,4-dioxane (60 mL) and water (6 mL) was added *para*-toluenesulfonic acid monohydrate (108 mg, 0.57 mmol, 0.17 equiv.) and the mixture was heated to 64 °C for 5 h. The reaction mixture was allowed to cool to room temperature and concentrated under reduced pressure. The so-obtained residue was partitioned between EtOAc (80 mL) and water (15 mL) and the phases were separated. The organic phase was washed with saturated brine (30 mL), dried ($MgSO_4$), and all volatiles were removed under reduced pressure. The so-obtained crude product was purified by column chromatography (SiO_2, *n*-hexane/EtOAc; 1:1, *v*/*v*) to give pure title compound (960 mg, 2.55 mmol, 75%) as a colorless solid.

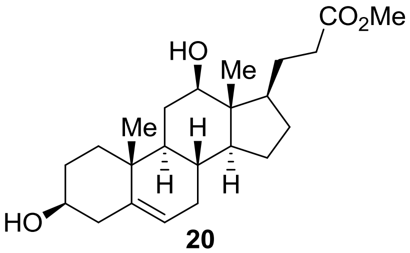

20

20: mp.: 122-125 °C; R_f: 0.23 (*n*-hexane/EtOAc, 1:1); $[\alpha]_D^{22}$: –31.7 (deg cm^3 g^{-1} dm^{-1}, c = 0.73, $CHCl_3$); IR (KBr): ν_{max} 3423, 2942, 2867, 2360, 1737, 1437, 1049, 1002, 953 cm^{-1}; ^{1}H-NMR (300 MHz, $CDCl_3$) δ 5.34 (m, 1 H), 3.67 (s, 3 H), 3.51 (m, 1 H), 3.46 (dd, *J* = 11.2, 4.6 Hz, 1 H), 1.02 (s, 3 H), 0.68 (s, 3 H); ^{13}C-NMR (75 MHz, $CDCl_3$) δ 175.2, 140.9, 121.6, 80.0, 71.8, 54.6, 51.8, 50.3, 49.9, 47.0, 43.3, 37.4, 36.8, 33.5, 31.7, 31.1, 30.6, 29.0, 27.7, 24.6, 19.5, 7.2; HRMS (m/z): $[M+Na]^+$ calculated for $C_{23}H_{36}O_4Na$: 399.25058, found: 399.25066; $[2M+Na]^+$ calculated for $C_{46}H_{72}O_8Na$: 775.51194, found: 775.51203.

3.12 (–)-3β,12β-Dihydroxypregn-5-ene-21-carboxylic acid (**21**):

To a solution of methyl ester **20** (960 mg, 2.55 mmol, 1.0 equiv.) in THF (50 mL) was added aqueous LiOH-solution (1.0 M, 50.0 mL, 50.0 mmol, 20 equiv.). The mixture was stirred at 30 °C for 4 h, allowed to cool to room temperature and neutralized with aqueous HCl (1.0 M, 50.0 mL, 50.0 mmol, 20 equiv.). The organic solvent was removed under reduced pressure and the remaining aqueous phase was extracted with EtOAc (5 x 150 mL). The combined organic layers were dried ($MgSO_4$), all volatiles were removed under reduced pressure and the so-obtained crude was purified by column chromatography (SiO_2, *n*-hexane/EtOAc; 1:1, *v*/*v*) to give pure title compound (831 mg, 2.30 mmol, 90%) as a colorless solid.

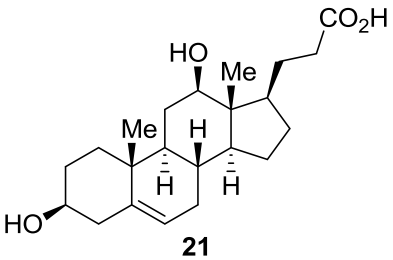

21

21: mp.: 205-208 °C; R_f: 0.30 (EtOAc); $[\alpha]_D^{22}$: –17.8 (deg cm^3 g^{-1} dm^{-1}, c = 0.90, MeOH); IR (KBr): ν_{max} 3428, 2942, 2820, 2360, 1683, 1271, 1049, 1017, 950 cm^{-1}; ^{1}H-NMR (300 MHz, MeOH-d_4) δ 5.34 (m, 1 H), 3.39 (m, 1 H), 3.30 (m, 1 H), 1.04 (s, 3 H), 0.69 (s, 3 H); ^{13}C-NMR (100 MHz, MeOH-d_4) δ 178.3, 142.2, 122.4, 80.7, 72.4, 55.9, 51.8, 50.3, 48.0, 42.9, 38.6,

37.8, 34.5, 32.7, 32.3, 32.2, 31.6, 29.0, 28.8, 25.5, 19.8, 7.7; HRMS (m/z): $[M+Na]^+$ calculated for $C_{22}H_{34}O_4Na$: 385.23493, found: 385.23477; $[2M+H]^+$ calculated for $C_{44}H_{69}O_8$: 725.49870, found: 725.49870; $[2M+Na]^+$ calculated for $C_{44}H_{68}O_8Na$: 747.48064, found: 747.48106.

3.13 (–)-3β,12β-Diformyloxypregn-5-ene-21-carboxylic acid (**10**):

A suspension of dihydroxycarboxylic acid **21** (831 mg, 2.30 mmol, 1.0 equiv.) in formic acid (98%, 16 mL) was heated to 50 °C for 30 min. The reaction mixture was allowed to cool to room temperature and under reduced pressure to give the crude product which was purified by column chromatography (SiO_2, *n*-hexane/EtOAc; 1:1, *v*/*v*) to give pure title compound (818 mg, 1.96 mmol, 85%) as a colorless solid.

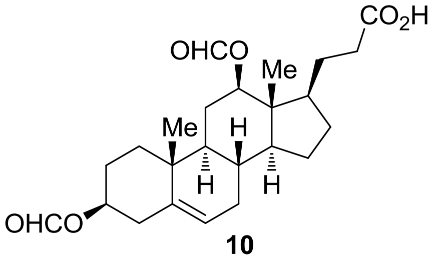

10

10: mp.: 64-65 °C; R_f: 0.50 (*n*-hexane/EtOAc, 1:1); $[\alpha]_D^{22}$: –52.4 (deg cm^3 g^{-1} dm^{-1}, c = 0.84, $CHCl_3$); IR (KBr): ν_{max} 3431, 2932, 1722, 1453, 1384, 1184, 932 cm^{-1}; ^{1}H-NMR (400 MHz, $CDCl_3$) δ 8.10 (s, 1 H), 8.03 (s, 1 H), 5.40 (m, 1 H), 4.82 (dd, *J* = 11.3, 4.6 Hz, 1 H), 4.73 (m, 1 H), 2.37 (m, 1 H), 2.33 (m, 2 H), 2.23 (m, 1 H), 1.04 (s, 3 H), 0.79 (s, 3 H); ^{13}C-NMR (100 MHz, $CDCl_3$) δ 179.4, 161.2, 160.7, 139.3, 122.8, 81.6, 73.8, 54.6, 49.7, 49.4, 45.4, 38.0, 37.0, 36.9, 33.2, 31.5, 31.0, 28.4, 27.7, 27.2, 27.1, 24.3, 19.3, 8.3; HRMS (m/z): $[M-H^+]^-$ calculated for $C_{24}H_{33}O_6$: 417.22816, found: 417.22800; $[2M-H^+]^-$ calculated for $C_{48}H_{67}O_{12}$: 835.46380, found: 835.46334.

3.14 (–)-3β-Acetoxypregn-5-ene-21,12β-carbolactone (**25**)

To a solution of the dihydroxycarboxylic acid **21** (250 mg, 0.69 mmol) in pyridine (8 mL) acetic anhydride (0.40 mL, 4.2 mmol, 6.0 equiv.) and 4-dimethylaminopyridine (21 mg, 0.17 mmol, 0.25 equiv.) were added at room temperature. The reaction mixture was stirred for 1.5 h and then concentrated to half the volume. The resulting solution was diluted with EtOAc (20 mL), washed with water (2 x 10 mL) and brine (10 mL), dried ($MgSO_4$) and evaporated to dryness under reduced pressure. Purification by column chromatography (SiO_2, *n*-hexane/EtOAc; 1:1, *v*/*v*) gave pure title compound (195 mg, 0.53 mmol, 77%) as colorless needles.

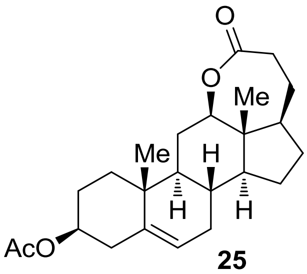

25

25: mp.: 65-68 °C; R_f: 0.40 (*n*-hexane/EtOAc, 2:1) $[\alpha]_D^{22}$: –16.2 (deg cm^3 g^{-1} dm^{-1}, c = 0.52, $CHCl_3$); IR (KBr): ν_{max} 2945, 1773, 1723, 1606, 1467, 1440, 1366, 1248, 1194, 1168, 1034, 788, 763 cm^{-1}; ^{1}H-NMR (400 MHz, $CDCl_3$) δ 5.38 (m, 1 H), 4.58 (m, 1 H), 4.11 (m, 1 H), 2.77 (dd, *J* =12.9, 5.6 Hz, 1 H), 2.33 (m, 1 H), 2.02 (s, 3 H), 1.03 (s, 3 H), 0.63 (s, 3 H). ^{13}C-NMR (100 MHz, $CDCl_3$) δ 175.4, 170.7, 139.7, 122.2, 84.5, 73.7, 54.5, 54.0, 49.4, 45.3, 38.1, 37.1,

36.8, 31.1, 30.4, 27.8, 27.5, 26.7, 23.8, 23.1, 21.6, 21.5, 19.4, 7.0; HRMS (m/z): $[M+Na]^+$ calculated for $C_{24}H_{34}O_4Na$: 409.23493, found: 409.23476.

3.15 (–)-21-*nor*-24-Diazo-3β,12β-diformyloxy-chol-5-ene-23-one (**3**):

To a solution of carboxylic acid **10** (200 mg, 0.48 mmol, 1.0 equiv.) in CH_2Cl_2 (10 mL) at room temperature was added oxalyl chloride (87 µL, 0.96 mmol, 2.0 equiv.) and DMF (1 drop) sequentially and the reaction mixture was stirred for 2 h. The so-obtained solution of the acid chloride was concentrated under reduced pressure and redissolved in THF (6 mL). To this solution at 0 °C was added a freshly prepared solution of diazomethane in Et_2O (ca. 0.2 M, 8.0 mL, ca. 1.6 mmol, ca. 3.3 equiv.) and the reaction mixture was kept at this temperature for 1 h. The resulting yellowish solution was allowed to warm to room temperature and stirred for another 1 h. The reaction mixture was quenched carefully with acetic acid (80 µL, 1.40 mmol, 2.9 equiv.) and all volatiles were removed under reduced pressure. The so-obtained crude product was purified by column chromatography (SiO_2, *n*-hexane/EtOAc; 3:1, *v*/*v*) to give pure title compound (180 mg, 0.406 mmol, 85%) as a yellow solid.

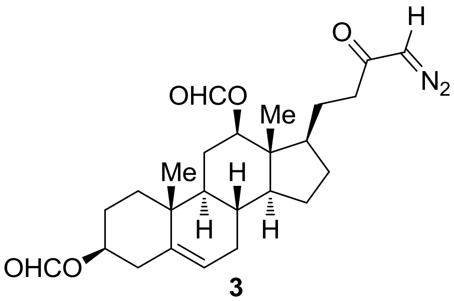

3

3: mp.: 68-70 °C; R_f: 0.50 (*n*-hexane/EtOAc, 1:1); $[\alpha]_D^{22}$: –26.4 (deg cm^3 g^{-1} dm^{-1}, c = 1.21, $CHCl_3$); IR (KBr): ν_{max} 2932, 2103, 1720, 1642, 1363, 1184, 930, 788, 763 cm^{-1}; ^{1}H-NMR (300 MHz, $CDCl_3$) δ 8.10 (s, 1 H), 8.03 (s, 1 H), 5.39 (m, 1 H), 5.19 (s (br), 1 H), 4.82 (dd, *J* = 11.4, 4.5 Hz, 1 H), 4.71 (m, 1 H), 2.36 (m, 1 H), 2.25 (m, 2 H), 1.04 (s, 3 H), 0.77 (s, 3 H); ^{13}C-NMR (100 MHz, $CDCl_3$) δ 195.2, 161.1, 160.7, 139.3, 122.8, 81.5, 73.7, 54.6, 49.9, 49.4, 45.5, 38.0, 37.0, 36.9, 31.5, 30.9, 28.5, 27.7, 27.6, 27.3, 24.3 (2 x C), 19.3, 8.3;[1] HRMS (m/z): $[M+Na]^+$ calculated for $C_{25}H_{34}N_2O_5Na$: 465.23599, found: 465.23613.

3.16 (–)-21,26,27-tri-*nor*-24-Diazo-3β,12β-diformyloxycholest-5-ene-23-one (**24**):

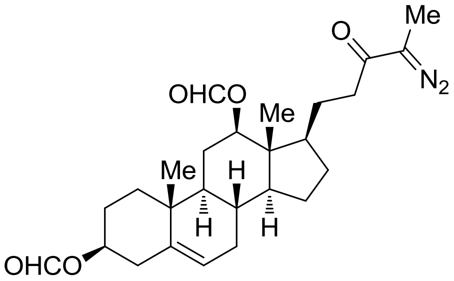

24

To a solution of carboxylic acid **10** (28.0 mg, 70.0 µmol, 1.0 equiv.) in CH_2Cl_2 (2 mL) at room temperature was added oxalyl chloride (13 µL, 140 µmol, 2.0 equiv.) and DMF (1 drop) sequentially and the reaction mixture was stirred for 2 h. The so-obtained solution of the acid chloride was concentrated under reduced pressure and

[1] One signal is hidden by doubling. NMR shift values of hidden signals were assigned by comparison with an assigned 2D-NMR of an analogous compound. The C24-signal is missing due to low concentration of the NMR sample and the quadrupolar coupling with N.

redissolved in THF (1 mL). To this solution at 0 °C was added a freshly prepared solution of diazoethane in Et_2O (ca. 0.2 M, 2.0 mL, ca. 0.4 mmol, ca. 5.7 equiv.) and the reaction mixture was kept at this temperature for 1 h. The resulting yellowish solution was allowed to warm to room temperature and kept at this temperature for another 3 h. All volatiles were removed under reduced pressure and the so-obtained crude product was purified by column chromatography (SiO_2, *n*-hexane/EtOAc; 3:1, *v*/*v*) to give pure title compound (14.7 mg, 32.0 µmol, 46%) as a yellow solid.

24: mp.: 50-51 °C; R_f: 0.40 (*n*-hexane/EtOAc, 2:1); $[\alpha]_D^{22}$: –43.3° (deg cm^3 g^{-1} dm^{-1}, c = 0.49, $CHCl_3$); IR (KBr): ν_{max} 2942, 2069, 1721, 1635, 1384, 1184, 932, 788 cm^{-1}; ^{1}H-NMR (400 MHz, $CDCl_3$) δ 8.09 (s, 1 H), 8.02 (s, 1 H), 5.40 (m, 1 H), 4.82 (dd, *J* = 11.1, 4.5 Hz, 1 H), 4.72 (m, 1 H), 1.09 (s, 3 H), 1.04 (s, 3 H), 0.78 (s, 3 H); ^{13}C-NMR (100 MHz, $CDCl_3$) δ 196.5, 161.1, 160.7, 139.3, 122.8, 81.5, 73.7, 54.6, 49.9, 49.4, 45.6, 38.0, 37.3, 37.0, 36.9, 31.6, 31.5, 30.9, 28.6, 27.7, 27.3, 24.3 (2 x C), 19.3, 8.3;[1] HRMS (m/z): $[M+H]^+$ calculated for $C_{26}H_{37}N_2O_5$: 457.26970, found: 457.26987; $[M+Na]^+$ calculated for $C_{26}H_{36}N_2O_5Na$: 479.25164, found: 479.25188; $[2M+Na]^+$ calculated for $C_{52}H_{72}N_4O_{10}Na$: 935.51407, found: 935.51462.

3.17 (–)-(17*R*)-spiro[3β,12β-Diformyloxyandrost-5-ene-17,3'-cyclopenta-1'-one] (**11**):

A suspension of rhodium(II) acetate dimer (6.2 mg, 14 µmol, 0.07 equiv.) in CH_2Cl_2 (3 mL) was degassed three times using *freeze-and-thaw* cycles. The resulting green suspension was heated to 41 °C and a solution of diazoketone **3** (88 mg, 0.20 mmol, 1.0 equiv.) in degassed CH_2Cl_2 (2.5 mL) was added dropwise. The mixture was kept at this temperature for 2 h, allowed to cool to room temperature, and all volatiles were removed under reduced pressure to obtain the crude product which was purified by column chromatography (SiO_2, *n*-hexane/EtOAc; 5:1, *v*/*v*) to give pure title compound (43 mg, 0.104 mmol, 52%) as colorless crystals.

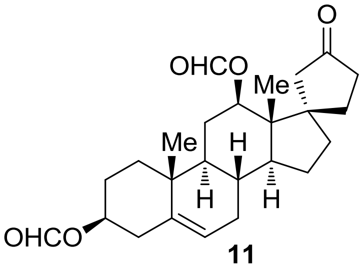

11

11: mp.: 122-126 °C; R_f: 0.20 (*n*-hexane/EtOAc, 5:1); $[\alpha]_D^{22}$: –33.4 (deg cm^3 g^{-1} dm^{-1}, c = 1.09, $CHCl_3$); IR (KBr): ν_{max} 2965, 1741, 1715, 1466, 1387, 1185, 931 cm^{-1}; ^{1}H-NMR (400 MHz, $CDCl_3$) δ 8.05 (s, 1 H), 8.01 (s, 1 H), 5.39 (m, 1 H), 5.04 (dd, *J* = 11.0, 4.8 Hz, 1 H), 4.76 (m, 1 H), 1.05 (s, 3 H), 0.98 (s, 3 H); ^{13}C-NMR (100 MHz, $CDCl_3$) δ 218.5, 160.6, 160.6, 139.2, 122.7, 76.7, 73.6, 53.6, 52.4, 48.9, 47.5, 47.1, 38.0, 37.3, 37.0, 36.8, 36.3, 32.8, 31.6, 31.5, 27.6, 27.1, 24.3, 19.3, 10.3; HRMS (m/z): $[M+Na]^+$ calculated for $C_{25}H_{34}O_5Na$: 437.22985, found: 437.22957; $[2M+Na]^+$ calculated for $C_{50}H_{68}O_{10}Na$: 851.47047, found: 851.46981.

3.18 (–)-(17*R*)-spiro[3β,12β-Dihydroxyandrost-5-ene-17,3'-cyclopenta-1'-one] (**22**):

To a solution of cyclopentanone **11** (82 mg, 0.20 mmol, 1.0 equiv.) in THF (4 mL) was added aqueous LiOH-solution (1.0 M, 4.0 mL, 4.0 mmol, 20 equiv.) and the resulting mixture was heated to 30 °C for 2 h. The reaction mixture was allowed to cool to room temperature, acidified with aqueous HCl (1.0 M, 4.2 mL, 4.2 mmol, 21 equiv.) and extracted with EtOAc (5 x 10 mL). The combined organic phases were dried ($MgSO_4$), all volatiles were removed under reduced pressure and the so-obtained crude product was purified by column chromatography (SiO_2, *n*-hexane/EtOAc; 1:1, v/v) to give pure title compound (67 mg, 0.186 mmol, 94%) as a colorless solid.

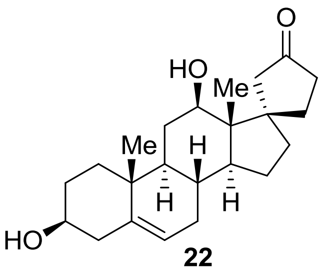

22

22: mp.: 123-125 °C; R_f: 0.23 (*n*-hexane/EtOAc, 1:1); $[\alpha]_D^{22}$: –26.1 (deg cm^3 g^{-1} dm^{-1}, c = 1.16, $CHCl_3$); IR (KBr): ν_{max} 3435, 2933, 1731, 1633, 1403, 1278, 1171, 1049, 1013, 956 cm^{-1}; ^{1}H-NMR (300 MHz, $CDCl_3$) δ 5.34 (m, 1 H), 3.68 (dd, *J* = 10.8, 4.5 Hz, 1 H), 3.50 (m, 1 H), 1.02 (s, 3 H), 0.86 (s, 3 H); ^{13}C-NMR (75 MHz, $CDCl_3$) δ 220.6, 140.8, 121.6, 74.7, 71.7, 53.6, 52.5, 49.4, 48.2, 47.9, 42.2, 37.5, 37.4, 36.7, 36.4, 33.1, 31.7, 31.6, 31.6, 31.4, 27.3, 19.5, 9.2; HRMS (m/z): $[M+Na]^+$ calculated for $C_{23}H_{34}O_3Na$: 381.24002, found: 381.24005; $[2M+Na]^+$ calculated for $C_{46}H_{68}O_6Na$: 739.49081, found: 739.49165.

3.19 (–)-(17*R*)-spiro[3β-*tert*-Butyldimethylsilyloxy-12β-hydroxyandrost-5-ene-17,3'-cyclopenta-1'-one] (**12**):

To a solution of diol **22** (66.0 mg, 0.180 mmol, 1.0 equiv.) in DMF (2 mL) at room temperature was added sequentially imidazole (50 mg, 0.72 mmol, 4.0 equiv.) and *tert*-butyldimethylsilyl chloride (68 mg, 0.45 mmol, 2.5 equiv.). After 1.5 h at this temperature, water (3 mL) was added and the mixture was extracted with CH_2Cl_2 (3 x 10 mL). The combined organic layers were dried ($MgSO_4$), all volatiles were removed under reduced pressure and the so-obtained crude product was purified by column chromatography (SiO_2, *n*-hexane/EtOAc; 4:1, *v*/*v*) to give pure title compound (83.0 mg, 0.176 mmol, 95%) as a colorless solid.

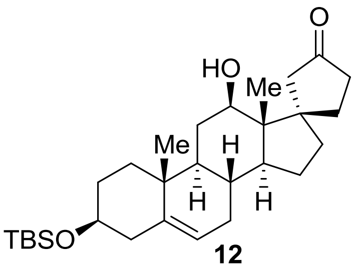

12

12: mp.: 193-195 °C; R_f: 0.23 (*n*-hexane/EtOAc, 1:1); $[\alpha]_D^{22}$: –10.6 (deg cm^3 g^{-1} dm^{-1}, c = 1.28, $CHCl_3$); IR (KBr): ν_{max} 3435, 2932, 2901, 2857, 1731, 1634, 1472, 1254, 1095, 888, 872, 837, 775 cm^{-1}; ^{1}H-NMR (300 MHz, $CDCl_3$) δ 5.30 (m, 1 H), 3.69 (dd, *J* = 10.9, 4.8 Hz, 1 H), 3.46 (m, 1 H), 2.41 (d, *J* = 16.8 Hz, 1 H), 2.34 (m, 1 H), 2.31 (d, *J* = 13.8 Hz, 1 H), 2.02 (d, *J* = 16.5 Hz, 1 H), 1.02 (s, 3 H), 0.88 (s, 9 H), 0.86 (s, 3 H), 0.05 (s, 6 H); ^{13}C-NMR (75 MHz, $CDCl_3$) δ 220.3, 141.4, 121.0, 74.7, 72.6, 53.7, 52.5, 49.5, 48.2, 48.0, 42.8, 37.5, 37.5,

36.8, 36.4, 33.2, 32.1, 31.7, 31.6, 31.5, 26.0, 24.8, 19.5, 18.4, 9.2, –4.5; HRMS (m/z): $[M+Na]^+$ calculated for $C_{29}H_{48}O_3SiNa$: 495.32649, found: 495.32598; $[2M+Na]^+$ calculated for $C_{58}H_{96}O_6Si_2Na$: 967.66376, found: 967.66378.

3.20 (+)-(17*R*)-spiro[14(13→12)abeo-3β-*tert*-Butyldimethylsilyloxyandrosta-5,12-dien-17,3'-cyclopenta-1'-one] (**13**):

To a solution of hydroxysteroid **12** (83 mg, 0.18 mmol, 1.0 equiv.) in toluene (12 mL) was added 4-dimethylaminopyridine (131 mg, 1.08 mmol, 6.0 equiv.) and *N*-(5-chloro-2-pyridyl)bis(trifluoromethanesulfonimide) (211 mg, 0.54 mmol, 3.0 equiv.) in one portion. The flask was immersed into an oil bath at a temperature of 130 °C and the reaction mixture kept at this temperature for 1 h. The suspension was allowed to cool to room temperature and was filtered through Celite washing several times with toluene. All volatiles were removed under reduced pressure and the so-obtained crude product was purified by column chromatography (SiO_2, *n*-hexane/EtOAc; 20:1, *v*/*v*) to give the title compound (71 mg, 0.16 mmol, 89%) as an inseparable mixture of *endo*- and *exo* alkene (*endo*:*exo*, 4:1, ratio determined by the ^{1}H-NMR integrals of the signals at 5.34 ppm (*endo*) and 4.89 ppm (*exo*)). To remove the minor isomer, the mixture was dissolved in EtOAc (4 mL) and rhodium on carbon (10%, 12 mg, 12 μmol, 0.07 equiv.) was added. The reaction mixture was flushed five times with H_2 and stirred for 1 h at room temperature under an H_2-atmosphere. The suspension was then filtered through Celite, washing several times with CH_2Cl_2, and concentrated under reduced pressure. The so-obtained residue was purified by column chromatography (SiO_2, *n*-hexane/EtOAc; 30:1, *v*/*v*) to yield pure title compound (56 mg, 0.12 mmol, 70%) as a colorless solid.

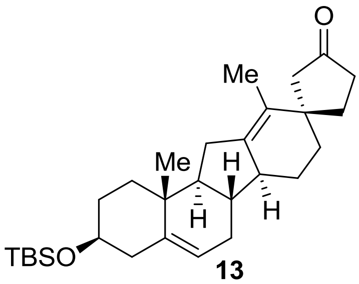

13

^{1}H-NMR of the mixture (300 MHz, $CDCl_3$) δ 5.34 (m, 1H, *exo*+*endo*), 4.89 (d, *J* = 1.8 Hz, 1H, *exo*), 4.87 (d, *J* = 1.6 Hz, 1H, *exo*), 3.50 (m, 1H, *exo*+*endo*), 2.57 (d, *J* = 18.0 Hz, 1H, *endo*), 1.59 (s, 3H, *endo*), 1.00 (s, 3H, *exo*), 0.98 (s, 3H, *endo*), 0.89 (s, 9H, *exo*+*endo*), 0.06 (s, 6H, *exo*+*endo*);

13: mp.: 180-184 °C; R_f: 0.25 (*n*-hexane/EtOAc, 20:1); $[\alpha]_D^{22}$: +19.6 (deg cm^3 g^{-1} dm^{-1}, c = 0.92, $CHCl_3$); IR (KBr): ν_{max} 2929, 2856, 1746, 1636, 1462, 1379, 1252, 1095, 877, 836, 775 cm^{-1}; ^{1}H-NMR (300 MHz, $CDCl_3$) δ 5.34 (m, 1 H), 3.48 (m, 1 H), 2.41 (d, *J* = 18.0 Hz, 1 H), 1.59 (s, 3 H), 0.98 (s, 3 H), 0.89 (s, 9 H), 0.06 (s, 6 H); ^{13}C-NMR (75 MHz, $CDCl_3$) δ 220.5, 142.6, 140.9, 126.7, 121.5, 72.8, 52.5, 51.1, 49.4, 44.4, 42.5, 42.4, 38.5, 36.8, 36.2, 35.7, 32.0, 31.7, 31.1, 29.0, 26.1, 24.4, 19.8, 18.4, 13.9, –4.4; HRMS (m/z): $[M+Na]^+$ calculated for $C_{29}H_{46}O_2SiNa$: 477.31593, found: 477.31591; $[2M+Na]^+$ calculated for $C_{58}H_{92}O_4Si_2Na$: 931.64264, found: 931.64341.

3.21 (–)-(17*R*)-spiro[14(13→12)abeo-3β-*tert*-Butyldimethylsilyloxyandrosta-5,12-dien-17,6'-pyrindan] (**23**):

A solution of C-*nor*-D-*homo* steroid **13** (36.0 mg, 80.0 μmol, 1.0 equiv.), propargylamine (10 μL, 0.16 mmol, 2.0 equiv.) and sodium[tetrachloroaurat(III)] dihydrate (2.9 mg, 8.0 μmol, 0.1 equiv.) in EtOH (3 mL) was prepared in a microwave vial. The vial was sealed and heated to 100 °C for 10 h. The reaction mixture was allowed to cool to room temperature and the so-obtained suspension was filtered through Celite, washing several times with CH_2Cl_2. All volatiles were removed under reduced pressure and the so-obtained crude product was purified by column chromatography (SiO_2, *n*-hexane/EtOAc; 10:1, *v*/*v*) to give pure title compound (17.4 mg, 36.0 μmol, 45%) as a colorless solid.

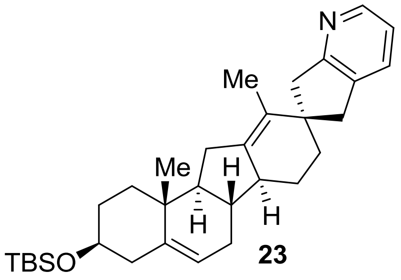

23

23: mp.: 135-138 °C; R_f: 0.25 (*n*-hexane/EtOAc, 10:1); $[\alpha]_D^{22}$: –57.1 (deg cm^3 g^{-1} dm^{-1}, c = 0.07, $CHCl_3$); IR (KBr): ν_{max} 2926, 2854, 1734, 1716, 1383, 1255, 1092, 836, 775 cm^{-1}; ^{1}H-NMR (300 MHz, $CDCl_3$) δ 8.34 (d, *J* = 5.0 Hz, 1 H), 7.43 (d, *J* = 7.4 Hz, 1 H), 7.03 (dd, *J* = 7.4, 5.0 Hz, 1 H), 5.35 (m, 1 H), 3.35 (m, 1 H), 3.42 (d, *J* = 17.2 Hz, 1 H), 3.01 (d, *J* = 16.8 Hz, 1 H), 2.63 (d, *J* = 17.2 Hz, 1 H), 2.60 (d, *J* = 16.8 Hz, 1 H), 1.55 (s, 3 H), 0.99 (s, 3 H), 0.90 (s, 9 H), 0.07 (s, 6 H); ^{13}C-NMR (75 MHz, $CDCl_3$) δ 164.8, 147.5, 142.6, 139.7, 136.3, 132.6, 128.0, 121.5, 121.3, 72.8, 52.6, 49.5, 46.8, 43.2, 42.6, 42.5, 38.5, 38.5, 36.8, 32.0, 31.2, 28.8, 26.1, 24.9, 18.9, 18.4, 14.7, –4.4; HRMS (m/z): $[M+H]^+$ calculated for $C_{32}H_{48}NOSi$: 490.34997, found: 490.34987; $[M+Na]^+$ calculated for $C_{32}H_{47}NOSiNa$: 512.33191, found: 512.33188.

3.22 Carbacyclopamine analog **2**:

To a solution of silylether **23** (6.0 mg, 12.0 μmol, 1.0 equiv.) in THF (0.5 mL) at room temperature was added tetrabutylammonium fluoride (1.0 M in THF, 36 μL, 36 μmol, 3.0 equiv.) and the reaction mixture was stirred for 10 h. After this time water (3 mL) was added and the mixture was extracted with CH_2Cl_2 (3 x 5 mL). The combined organic layers were dried ($MgSO_4$), all volatiles were removed under reduced pressure and the so-obtained crude product was purified by column chromatography (SiO_2, $CHCl_3$ + 3% MeOH) to give the title compound in ca. 90% purity judged by ^{1}H-NMR (3.6 mg, 9.9 μmol, 79%) as a colorless solid.

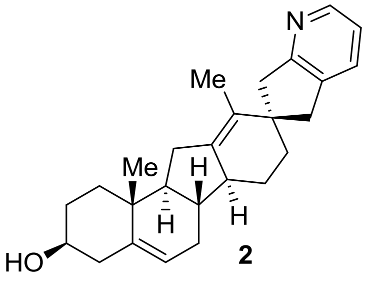

2

2: mp.: 170-175 °C; R_f: 0.53 ($CHCl_3$/MeOH, 95:5); $[\alpha]_D^{22}$: –33.0 (deg cm^3 g^{-1} dm^{-1}, c = 0.12, $CHCl_3$); IR (CCl_4): ν_{max} 3420, 2929, 2875, 2854, 1733, 1716, 1457, 1436, 1384, 1260, 1092,

1072, 786, 763 cm^{-1}; ^{1}H-NMR (700 MHz, $CDCl_3$) δ 8.34 (d, J = 4.9 Hz, 1 H, H-2'), 7.43 (d, J = 7.4 Hz, 1 H, H-4'), 7.03 (dd, J = 7.4, 4.9 Hz, 1 H, H-3'), 5.39 (m, 1 H, H-6), 3.56 (m, 1 H, H-3), 3.42 (d, J = 17.1 Hz, 1 H, H-7'), 3.01 (d, J = 16.5 Hz, 1 H, H-5'), 2.69 (d, J = 17.1 Hz, 1 H, H-7'), 2.60 (d, J = 16.5 Hz, 1 H, H-5'), 2.39 (ddd, J = 2.1, 4.6, 12.6 Hz, 1 H, H-4), 2.27 (m, 1 H, H-4), 2.24 (m, 2 H, H-7 + H-11), 2.08 (m, 1 H, H-11), 1.87 (m, 1 H, H-14), 1.86 (m, 1 H, H-16), 1.83 (m, 2 H, H-2 + H-15), 1.78 (m, 1 H, H-7), 1.74 (m, 1 H, H-1), 1.56 (s, 3 H, H-18), 1.53 (m, 1 H, H-2), 1.47 (dt, J = 9.1, 11.6 Hz, 1 H, H-9), 1.22 (m, 1 H, H-8), 1.20 (m, 1 H, H-1), 1.14 (m, 1 H, H-15), 1.00 (s, 3 H, H-3); ^{13}C-NMR (176 MHz, $CDCl_3$) δ 164.9 (C-7a'), 147.6 (C-2'), 141.8 (C-5), 139.6 (C-12), 136.4 (C-4a'), 132.6 (C-4'), 128.0 (C-13), 122.1 (C-6), 121.3 (C-3'), 72.1 (C-3), 52.4 (C-9), 49.5 (C-14), 46.9 (C-7'), 45.8 (C-17), 43.2 (C-5'), 42.4 (C-8), 38.5 (C-16), 38.3 (C-1), 36.7 (C-10), 31.5 (C-2), 31.1 (C-7), 28.7 (C-11), 24.9 (C-16), 18.8 (C-18), 14.7 (C-19); HRMS (m/z): $[M+H]^+$ calculated for $C_{26}H_{34}NOSi$: 376.26349, found: 376.26386; $[M+Na]^+$ calculated for $C_{26}H_{33}NOSiNa$: 398.24544, found: 398.24593.

4. Acid Stability

To a solution of carbacyclopamine analog **2** (2.0 mg, 5.5 µmol) in THF (0.5 mL) was dropwise added aqueous HCl (2.0 M) until a pH value of approx. 0.3 was reached. The mixture was stirred for 1 h at room temperature after which the solution was neutralized with saturated $NaHCO_3$-solution, extracted with CH_2Cl_2 (3 x 5 mL), dried ($MgSO_4$) and filtered. All volatiles were removed under reduced pressure and the so-obtained white solid (2.0 mg, 5.5 µmol) was used directly for H-NMR measurements. The ^{1}H-NMR spectra recorded before and after treatment with acid are shown in Figure 1.

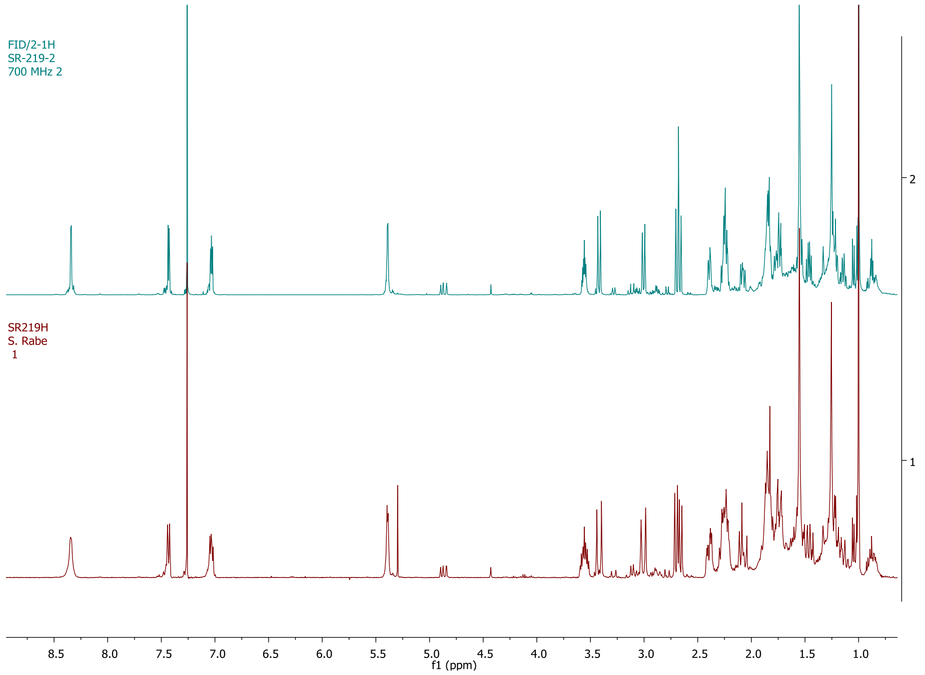

Figure S1: ^{1}H-NMR spectra of carbacyclopamine analog **2** in $CDCl_3$ before (700 MHz, blue) and after treatment with hydrochloric acid at pH 0.3 (400 MHz, maroon).

5. Biochemistry

The interference of the carbacyclopamine analog **2** with the hedgehog signaling pathway was tested in an established reporter gene assay [1] based on the inhibition of the target gene Gli1. Shh-LIGHTII cells (ATCC CRL-2795, LGC, Wesel, Germany) represent a clonal mouse fibroblast cell line (NIH 3T3), which stably incorporates a Gli-dependent firefly luciferase reporter and a constitutive Renilla luciferase reporter. They were grown in 75 cm^3 cell culture flasks at 37°C in a humid atmosphere with 5% CO_2. Cell growth medium DIMETHOXYETHANEM (Dulbecco's Modified Eagle's Medium, high glucose, sodium pyruvate, w/o glutamine), ZeocinTM Selection Reagent and Geneticin® Selection Antibiotic (G418 sulfate) were obtained from Invitrogen. Additive L-glutamin and trypsin were obtained from PAA. VerseneTM chelating agent was obtained from Gibco, bovine fetal serum (FBS) from Sigma. The cell freeze medium contained 5% DMSO in complete growth medium. The cell number was counted using a Neubauer-Zählkammer (Hemocytometer). DIMETHOXYETHANEM (high glucose, sodium pyruvate, w/o glutamine) supplemented with 0.5% FBS, 4 mM L-glutamin and 50 mM HEPES buffer (4-(2-hydroxyethyl)-1-piperazine ethanesulfonic acid) (pH 7.4) was used as incubation medium. The measurement of reporter gene and constitutive Renilla luminescence was performed with the Dual Luciferase® reporter system according to the manufacturer's instructions (Promega, Mannheim, Germany) using a GENios reader (TECAN, Crailsheim, Germany).

5.1 Luciferase reporter assay

Incubation of cells. For performing the assay, Shh-LIGHTII cells were grown to reach 80% confluence, washed twice with Versene, detached with 2 mL trypsin for not longer than 2 min and resuspended in 8 mL of growth medium. After centrifugation for 3 min, the supernatant was removed and the cell pellet dissolved in approximately 10 mL of growth medium. Finally, 20,000 to 100,000 cells per well were cultured in a 24-well plate for 48 h. For exposure to the analog the growth medium was removed and the cells were exposed to the compound in 500 µL/well incubation medium for 48 h. Stock solutions were prepared in EtOH and introduced in different concentrations into the incubation medium to reach a final solvent concentration of 0.05%. Due to the assay principle, Shh-LIGHTII cells had to be co-exposed to 100 nM of SAG (Smoothened agonist) for determining the inhibitory activity of the compounds. A SAG stock solution was prepared in EtOH and introduced into the medium. The final EtOH-concentration – introduced by SAG and the test compound – was 0.1%. As a positive control a 100 nM SAG solution in incubation medium (0.1% EtOH) was used, negative controls were treated with EtOH only.

Performing the luciferase measurement. After 48 h of incubation cells were washed with 500 µL PBS (phosphate buffered saline, w Mg/Ca, Invitrogen) and afterwards incubated for 15 min with 100 µL 1x passive lysis buffer on a shaker. The lysed cells together with lysis solution were transferred into 1.5 mL reaction tubes, centrifuged for 1 min at 4°C and kept on ice until further use. At first, the blank of luminescence of a 96-well plate (flat bottom, white, Greiner bio-one) was recorded. Then, 100 µL luciferase assay reagent and 10 µL cell supernatant were mixed per well and the luminescence of firefly luciferase was recorded. Addition of 100 µL Stop&Glo® reagent permitted the recording of Renilla luminescence. The measurement was performed per row of a well, i.e. assay reagent was added to 8 wells and luminesescence recorded, before the next row was processed. Analysis of constitutive Renilla luminescence was used to normalize for any potential unspecific Gli1-reporter gene luminescence.

6. NMR Spectra

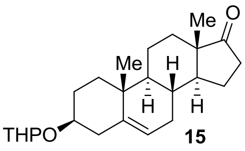

15

^{1}H-NMR (400 MHz, $CDCl_3$)

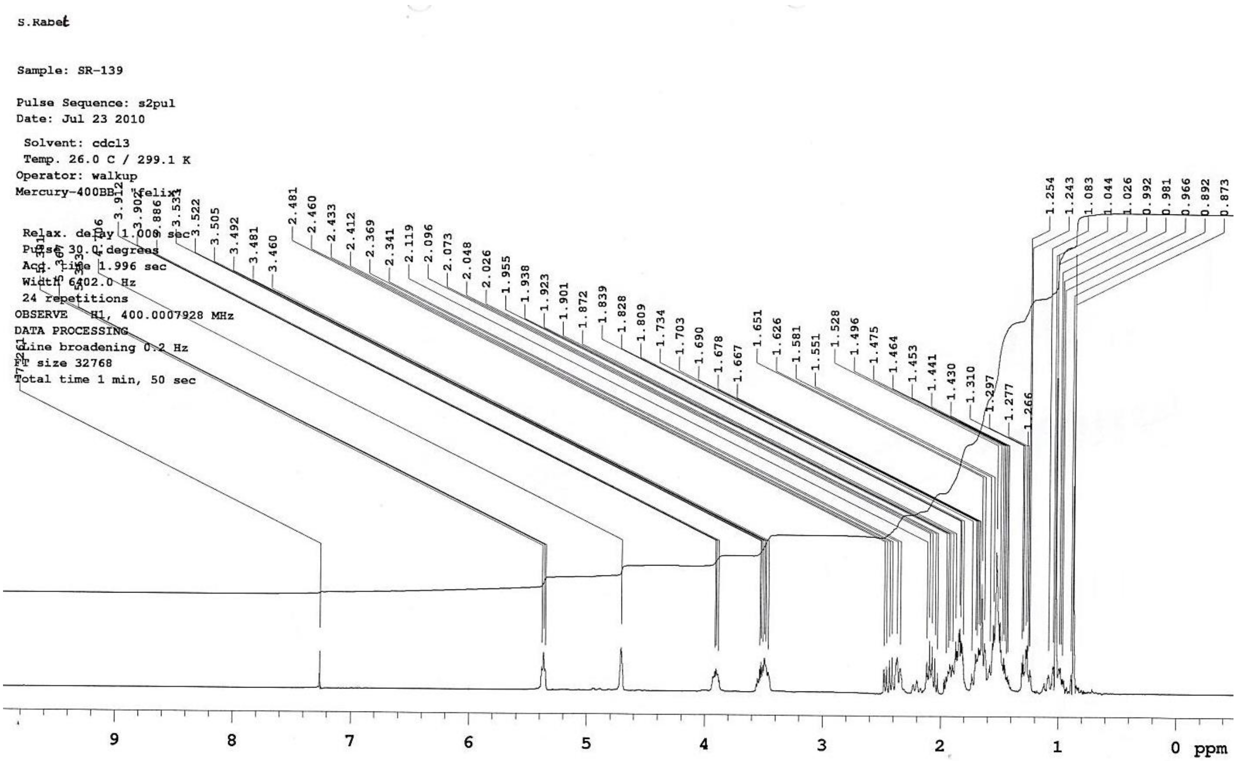

^{13}C-NMR (100 MHz, $CDCl_3$)

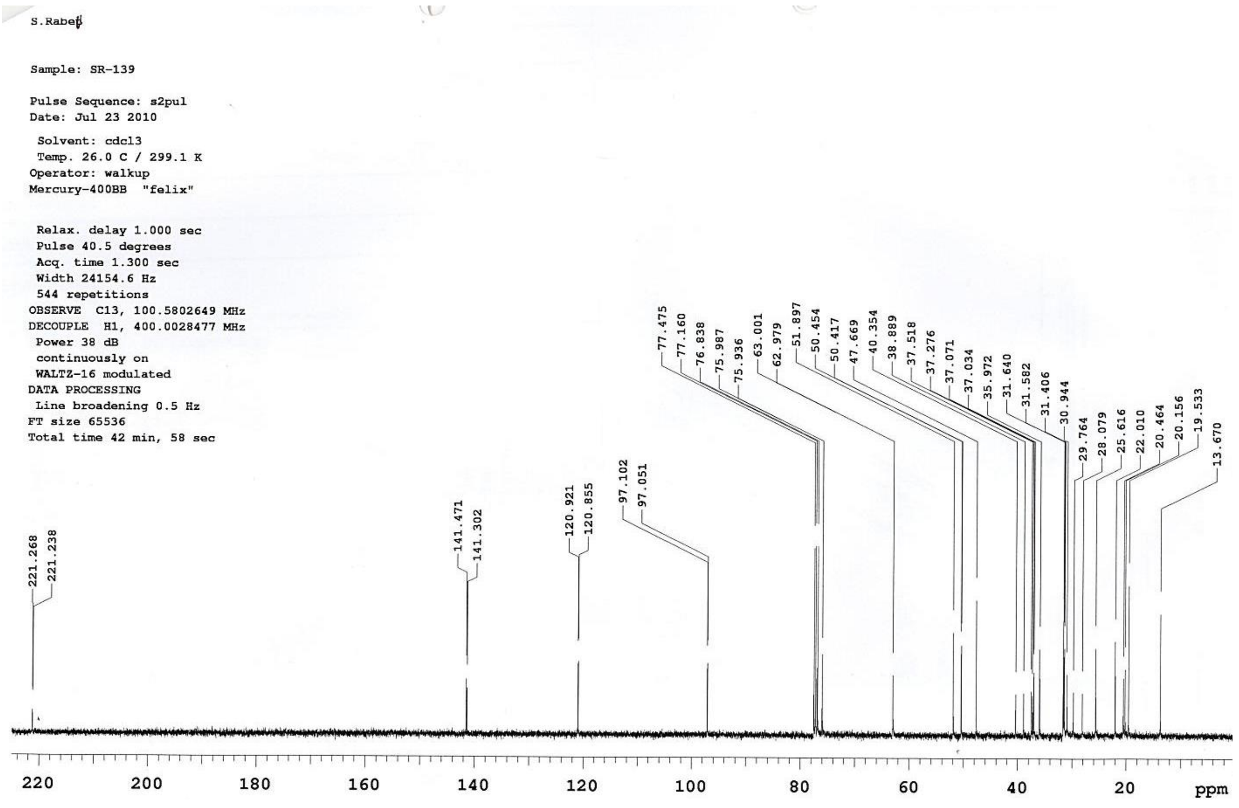

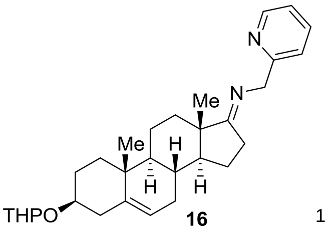

16

^{1}H-NMR (300 MHz, $CDCl_3$)

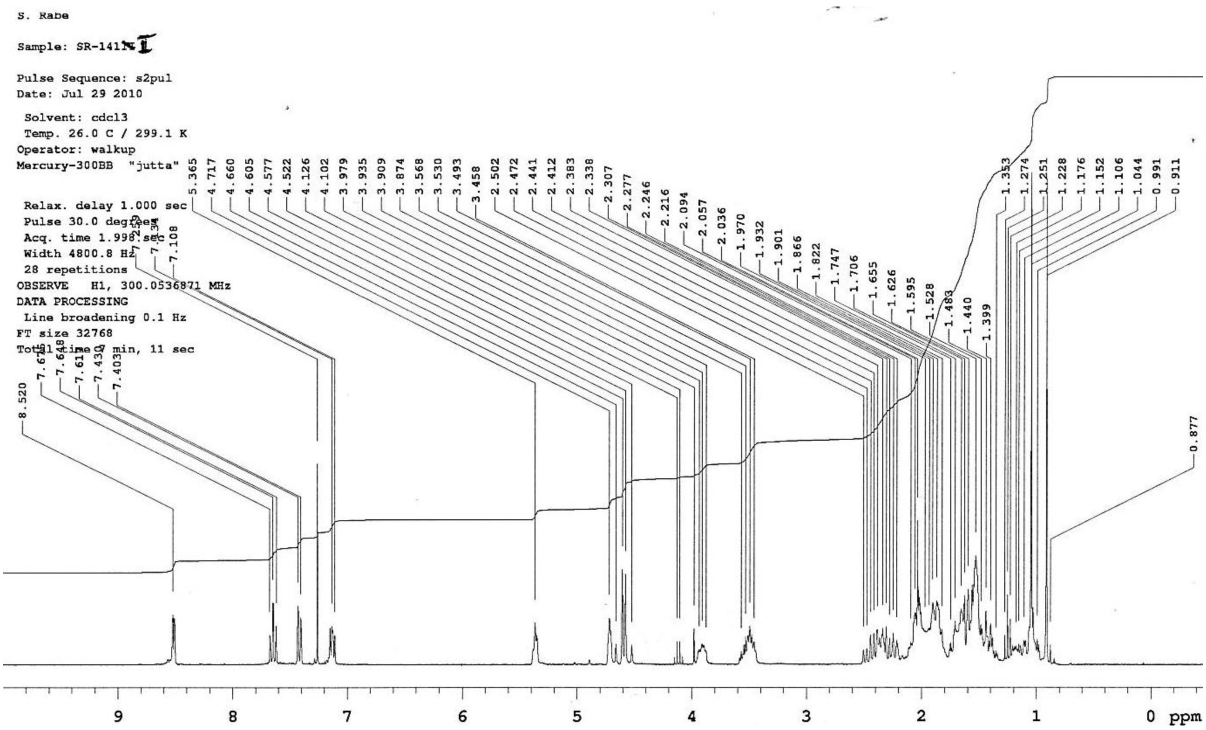

^{13}C-NMR (100 MHz, $CDCl_3$)

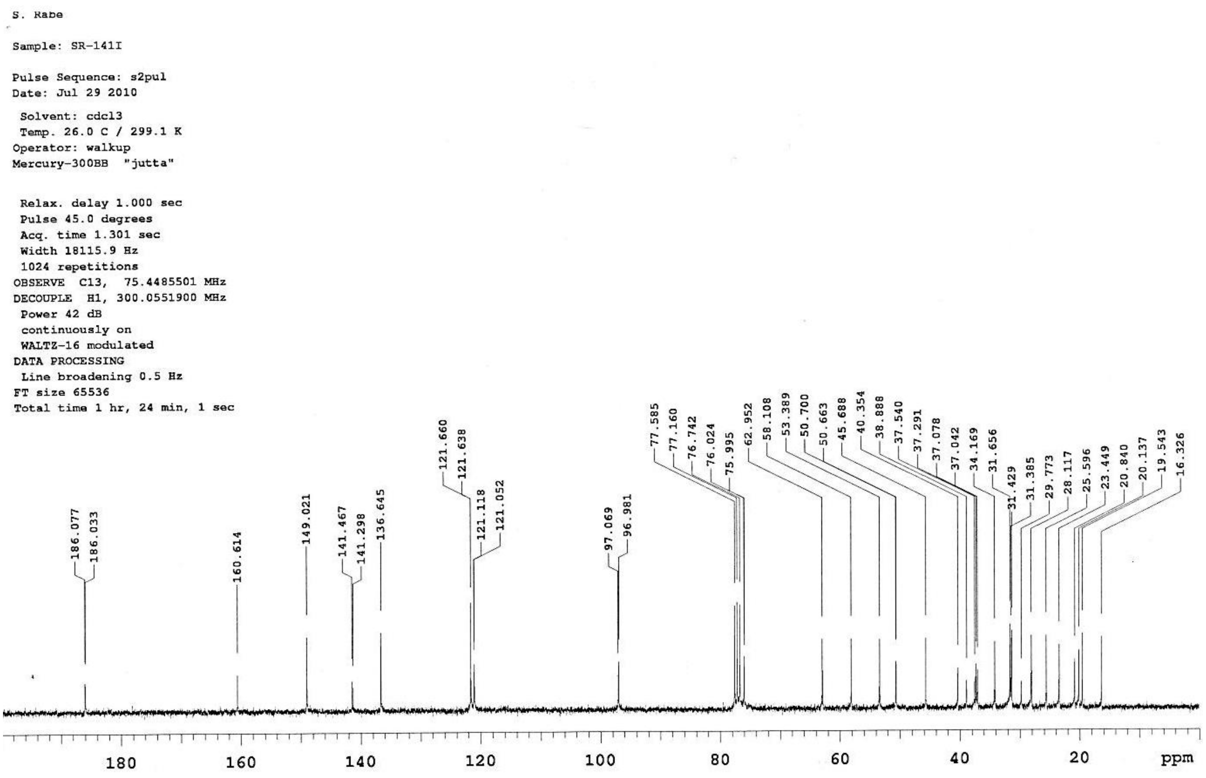

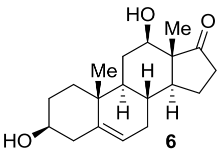

6 ^{1}H-NMR (400 MHz, CD_3OD)

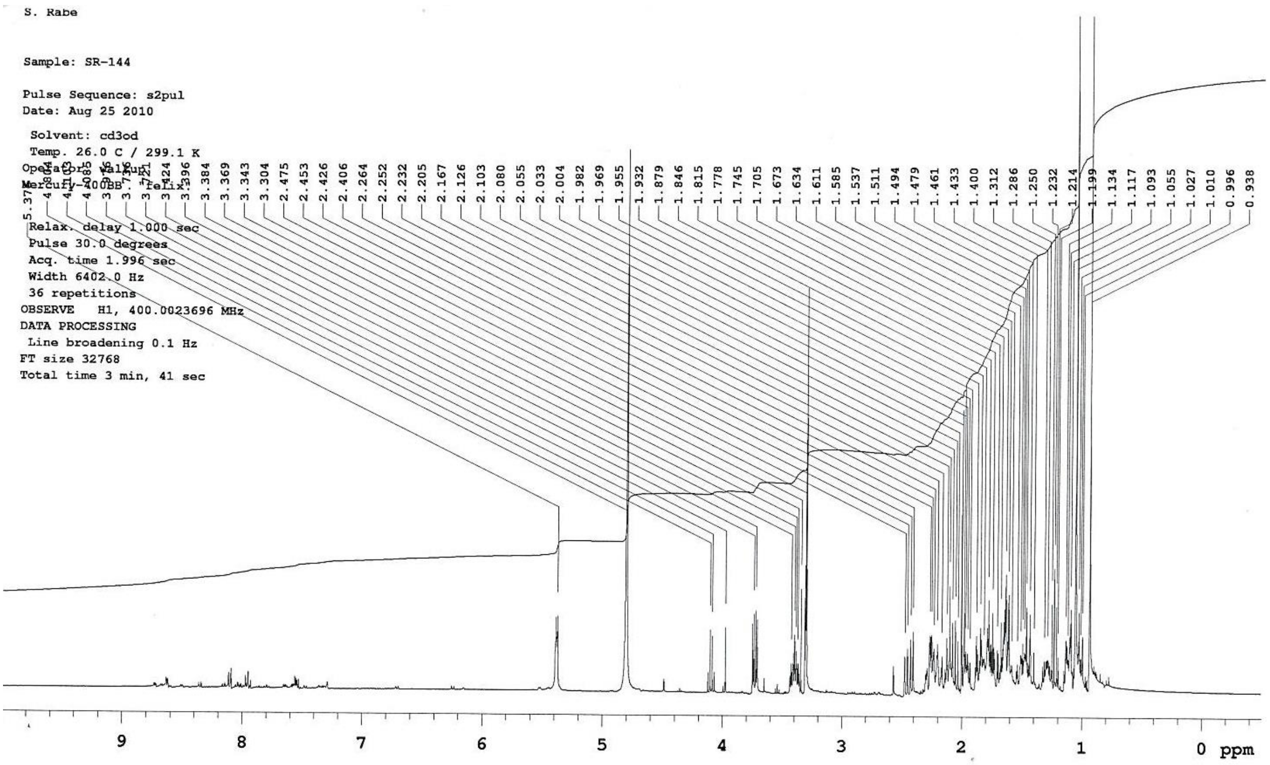

^{13}C-NMR (100 MHz, CD_3OD)

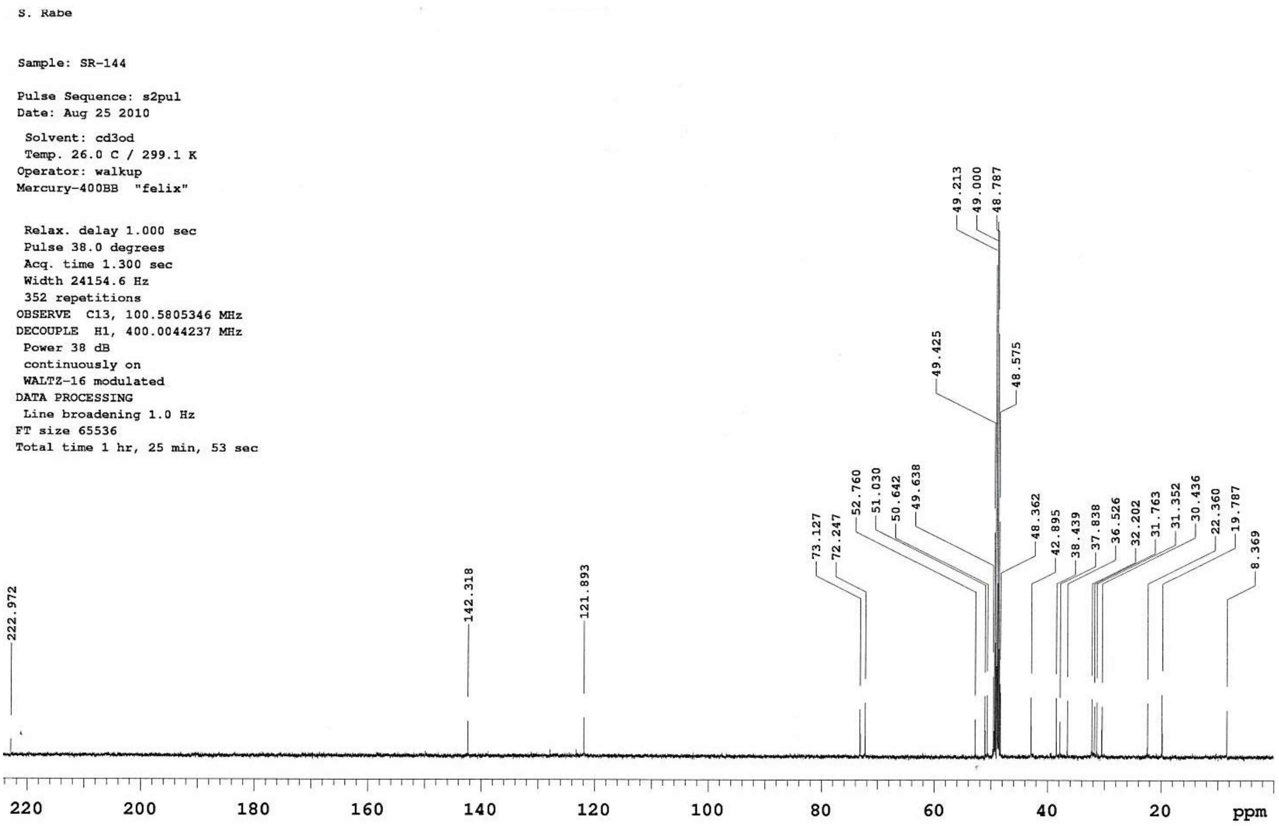

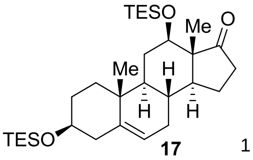

17

^{1}H-NMR (300 MHz, $CDCl_3$)

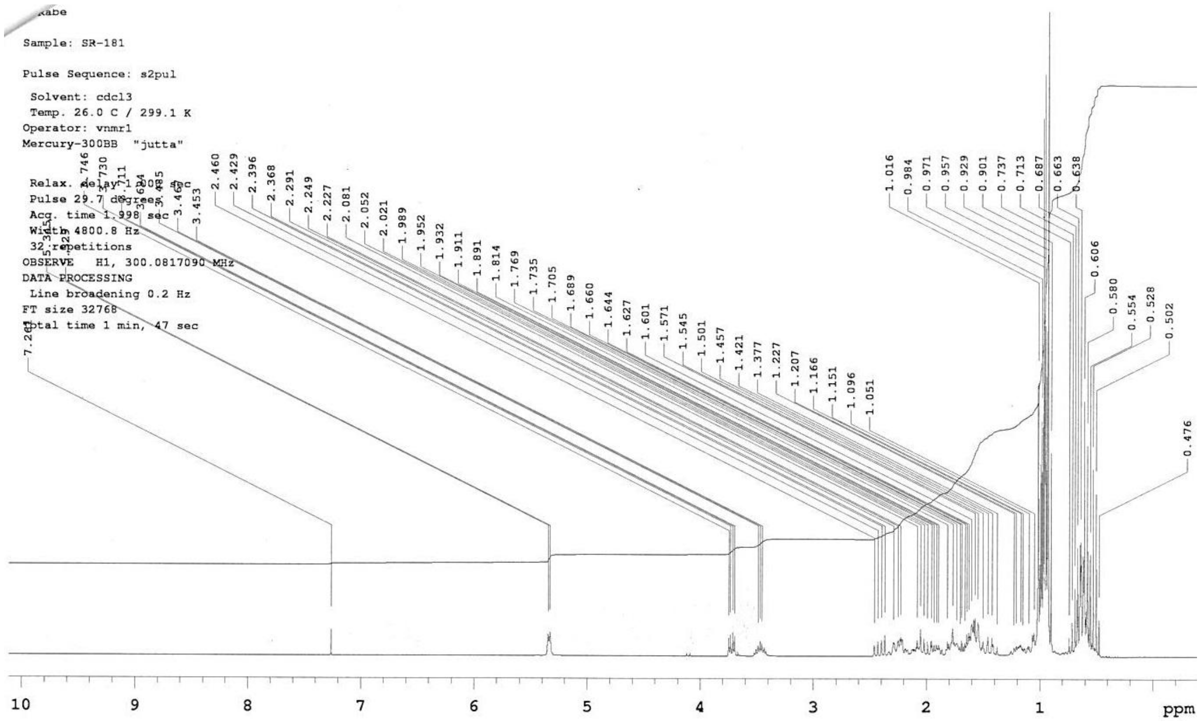

^{13}C-NMR (75 MHz, $CDCl_3$)

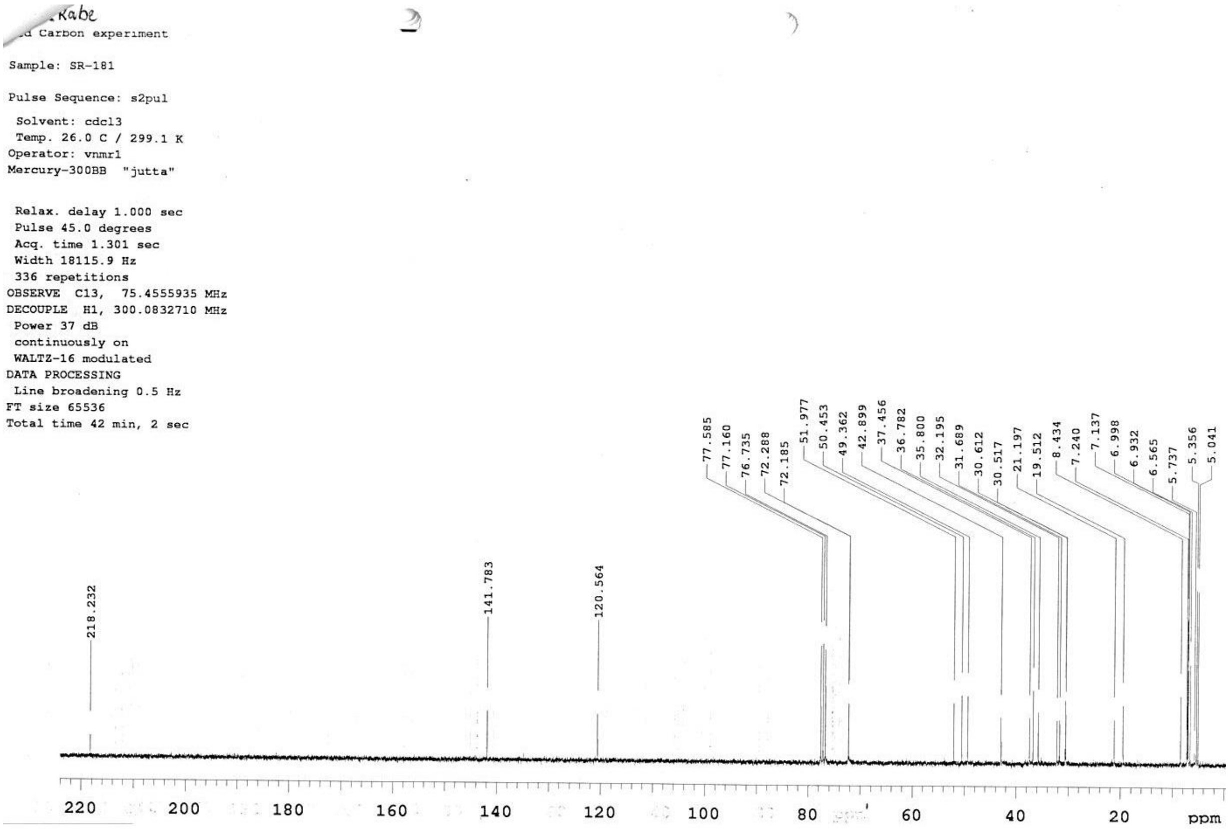

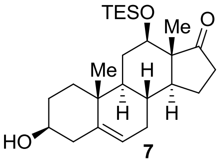

7

^{1}H-NMR (400 MHz, $CDCl_3$)

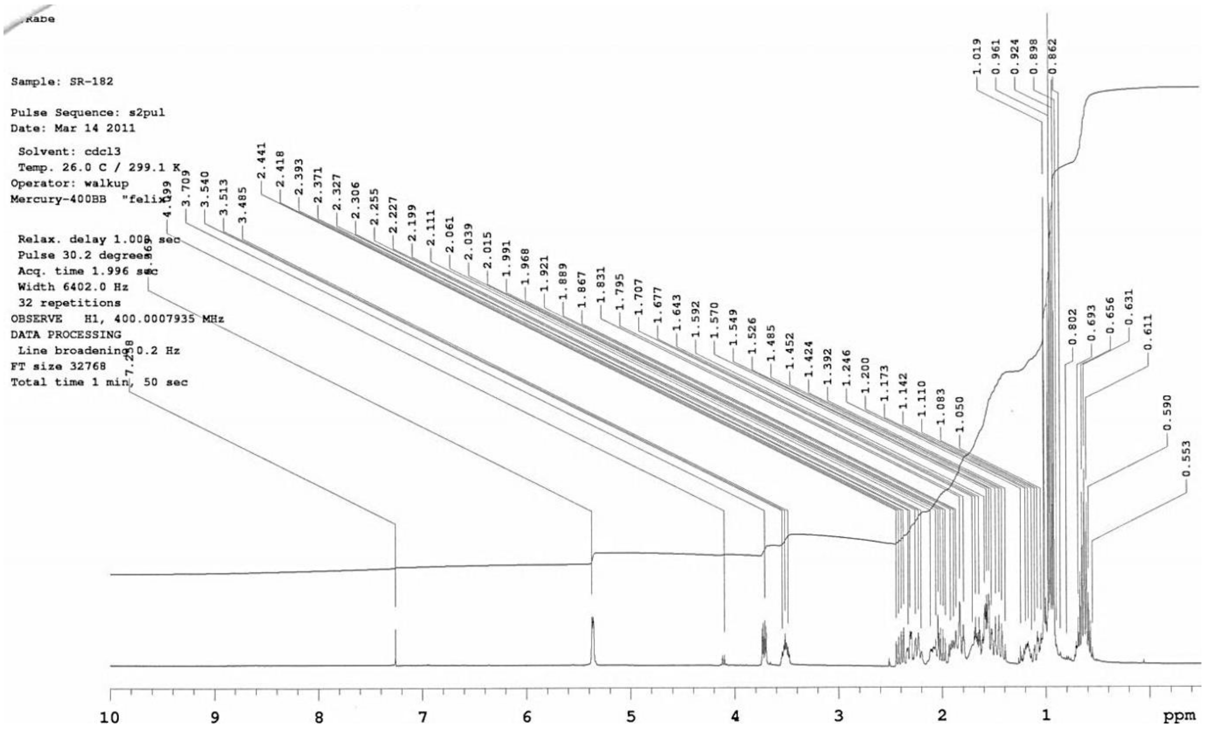

^{13}C-NMR (100 MHz, $CDCl_3$)

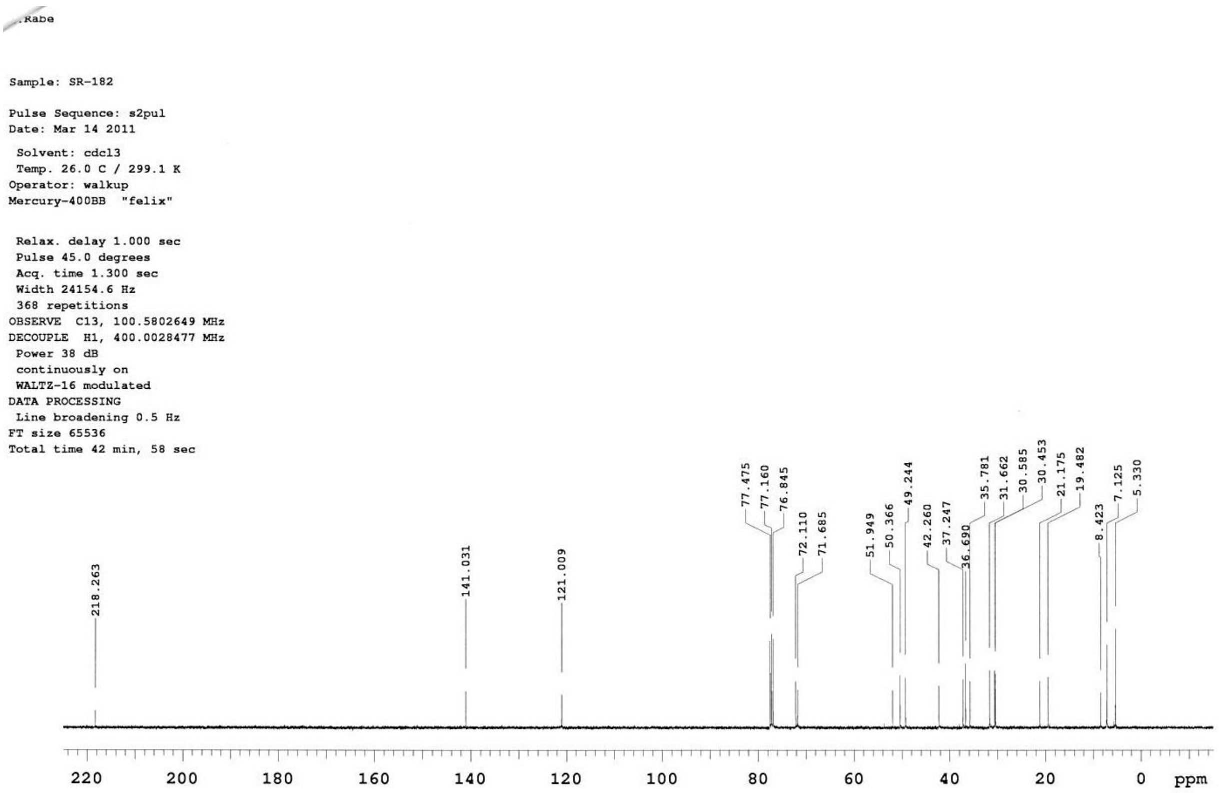

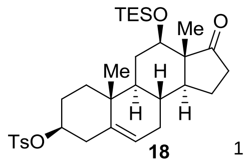

18

^{1}H-NMR (400 MHz, $CDCl_3$)

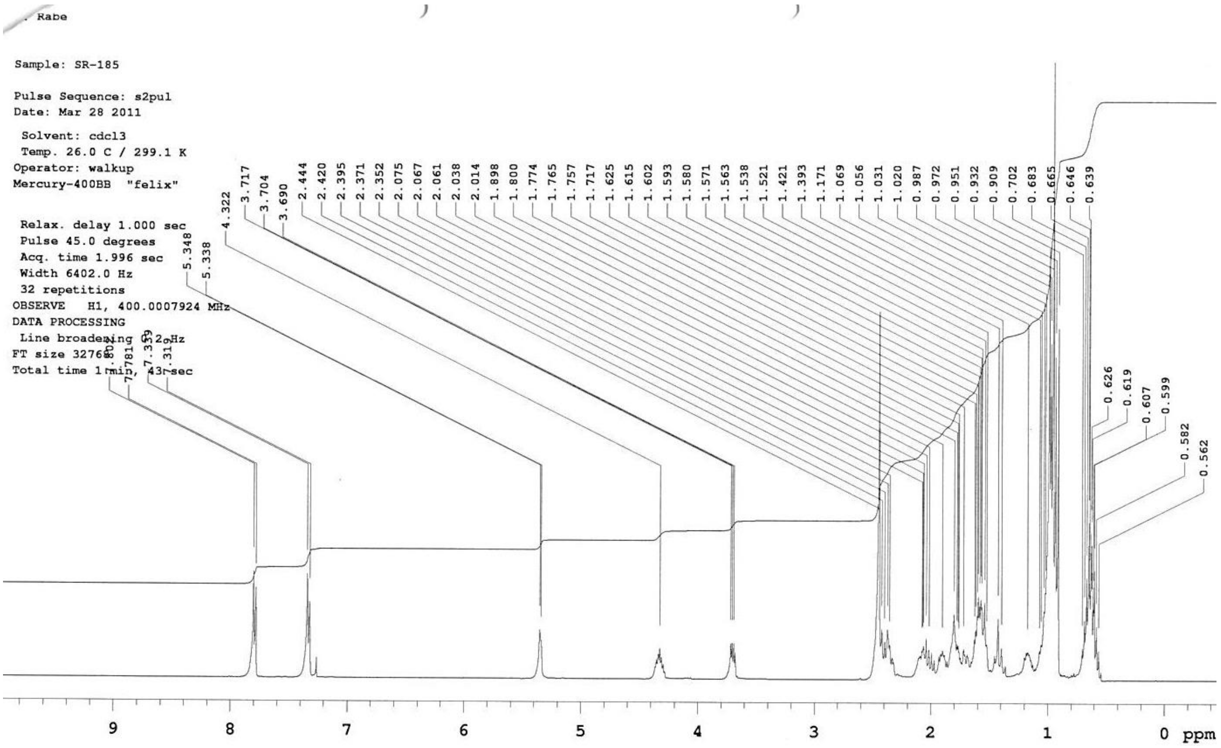

^{13}C-NMR (100 MHz, $CDCl_3$)

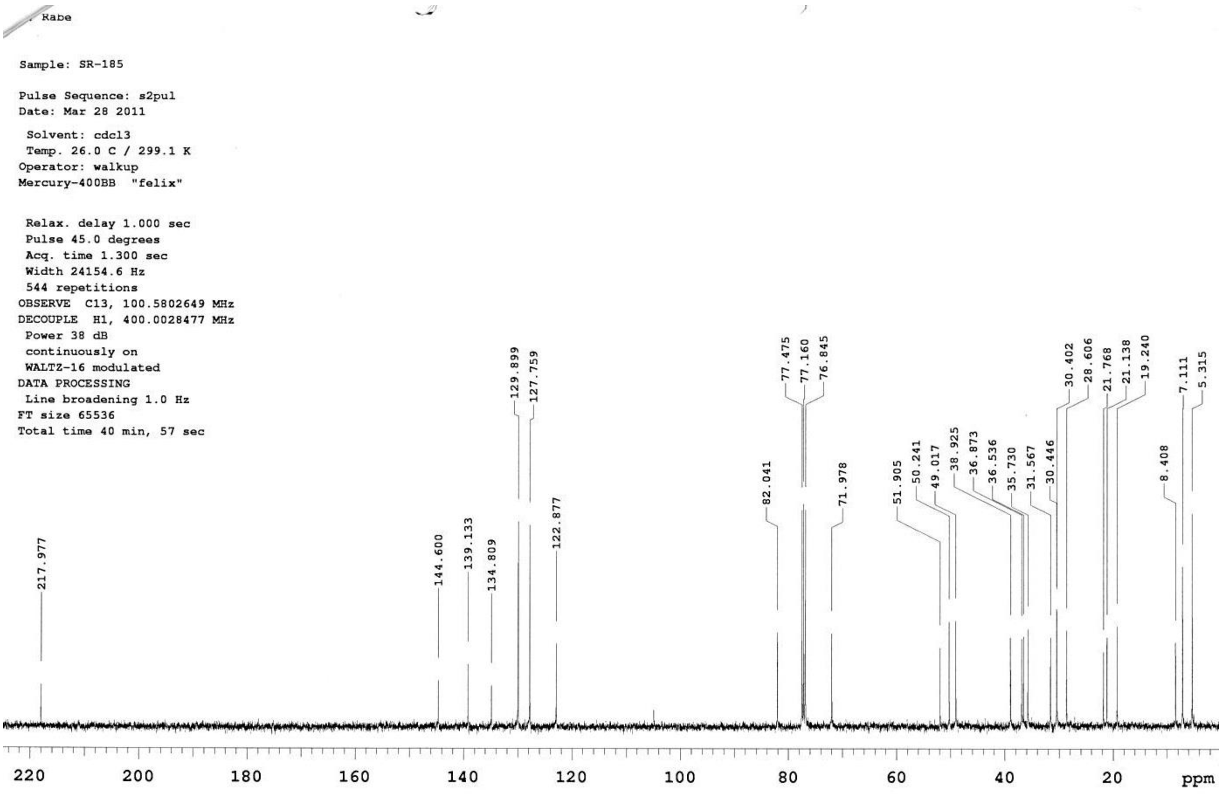

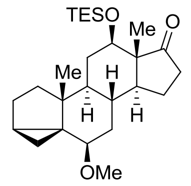

8

^{1}H-NMR (400 MHz, $CDCl_3$)

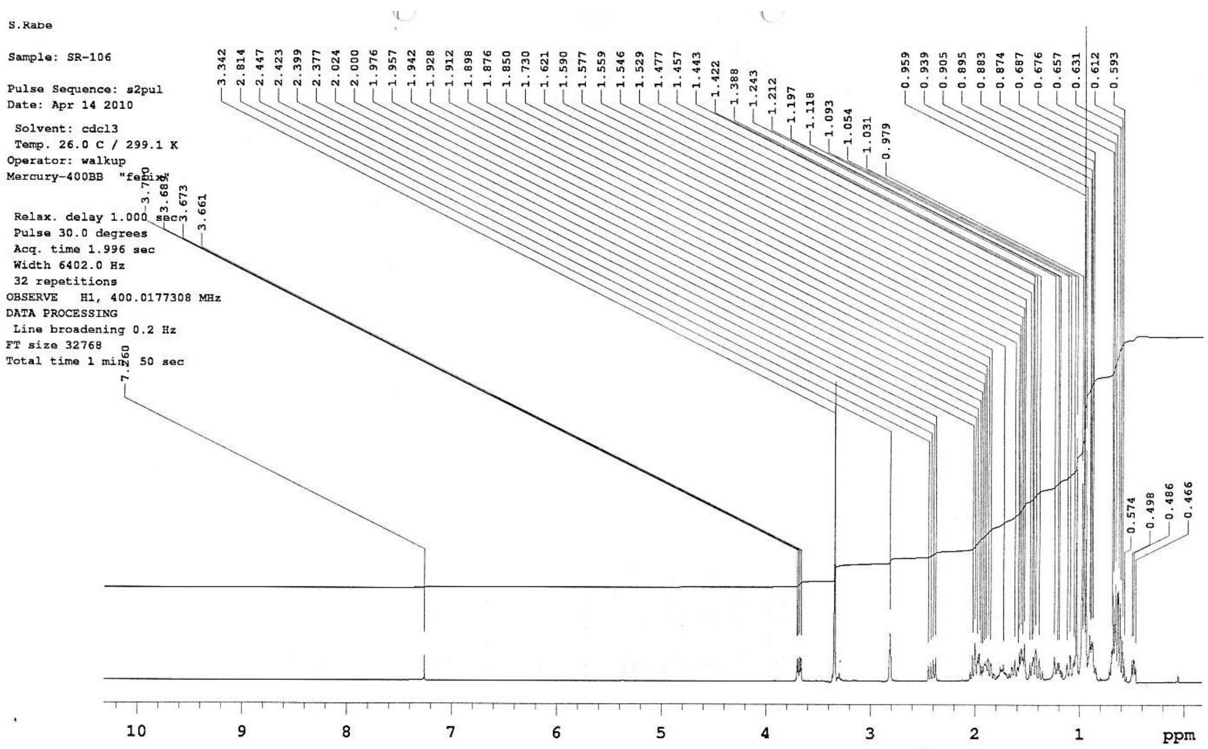

^{13}C-NMR (100 MHz, $CDCl_3$)

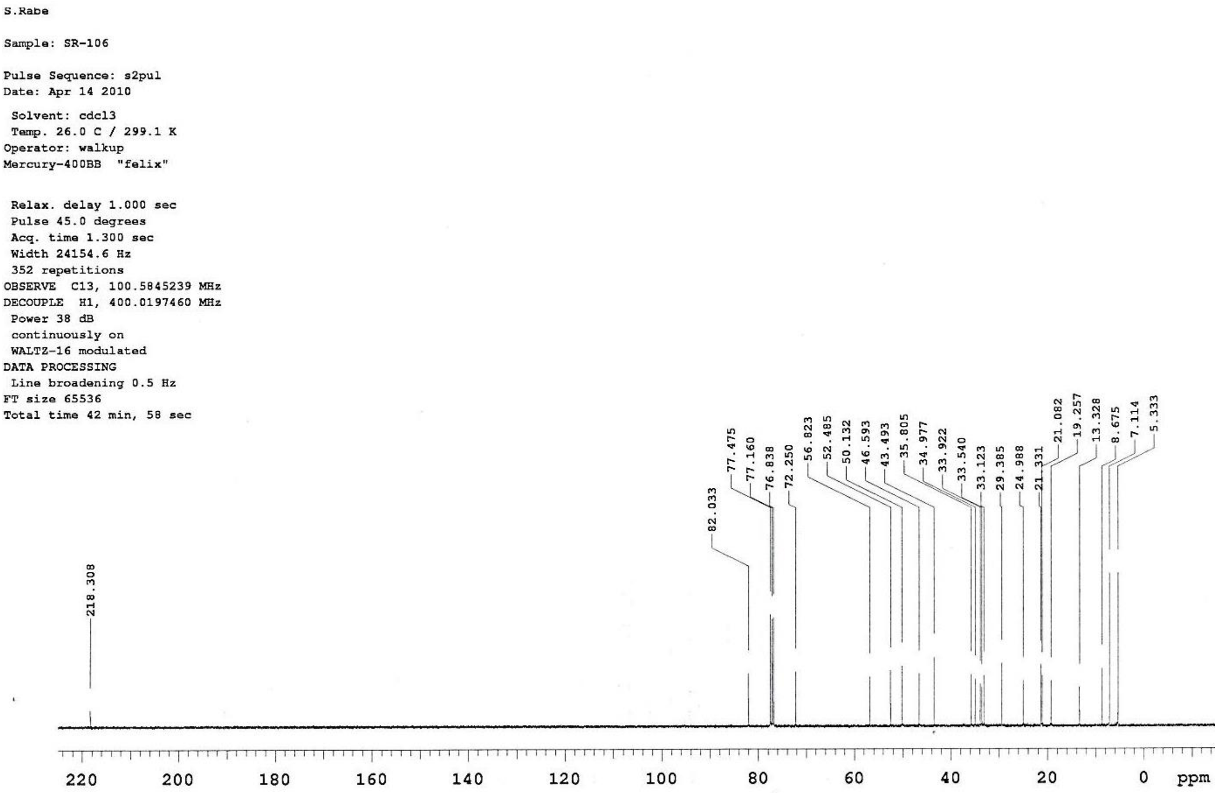

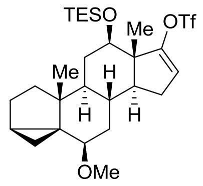

19

^{1}H-NMR (400 MHz, C_6D_6)

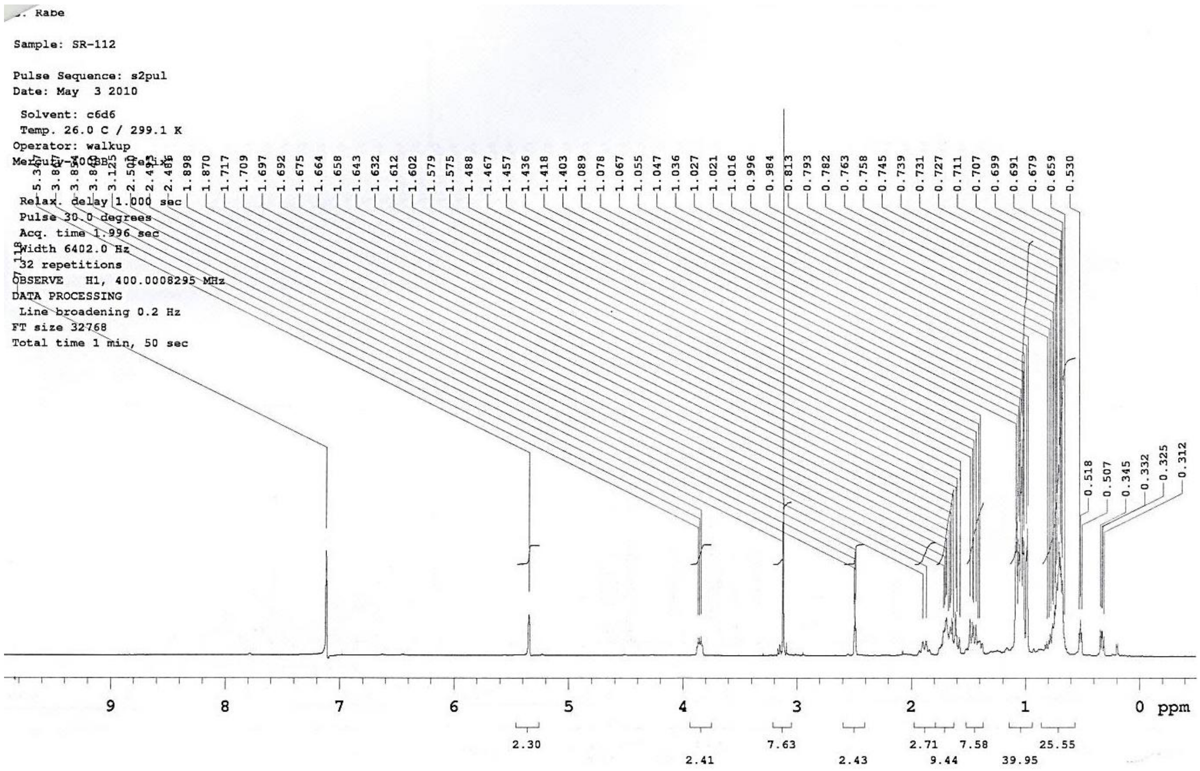

^{13}C-NMR (100 MHz, $CDCl_3$)

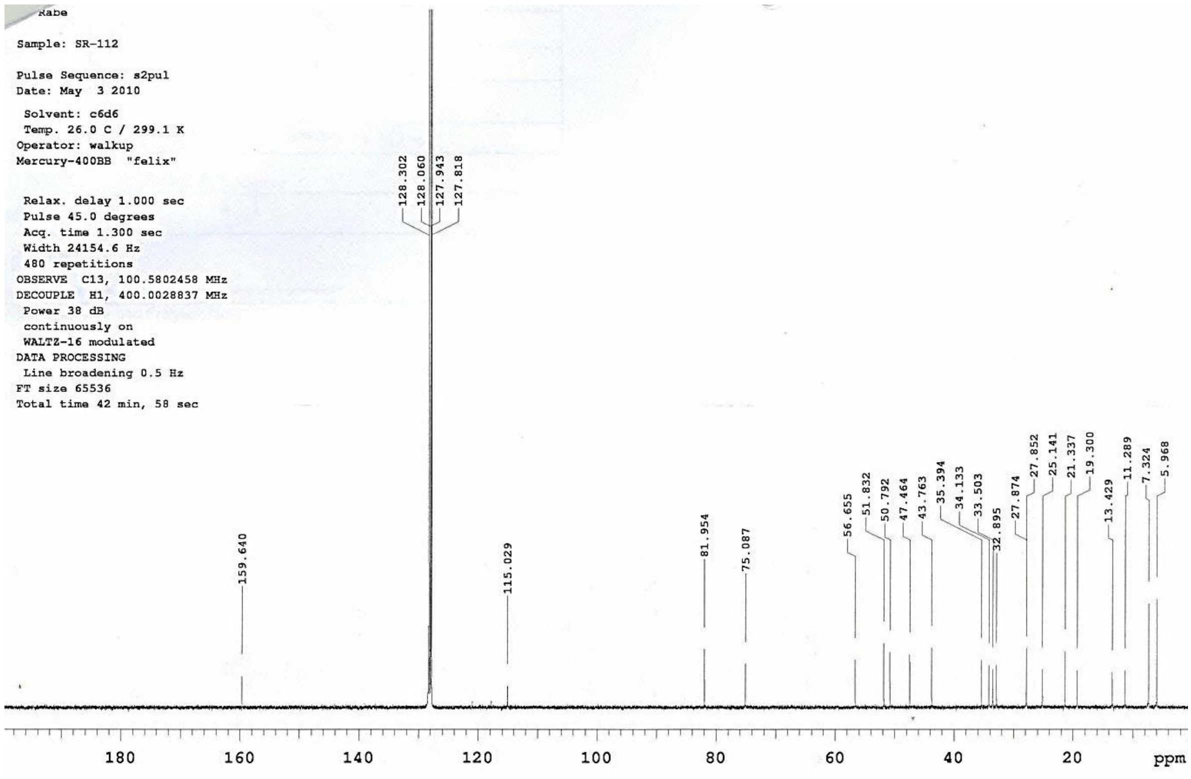

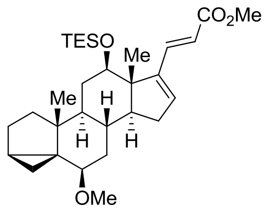

4

^{1}H-NMR (300 MHz, $CDCl_3$)

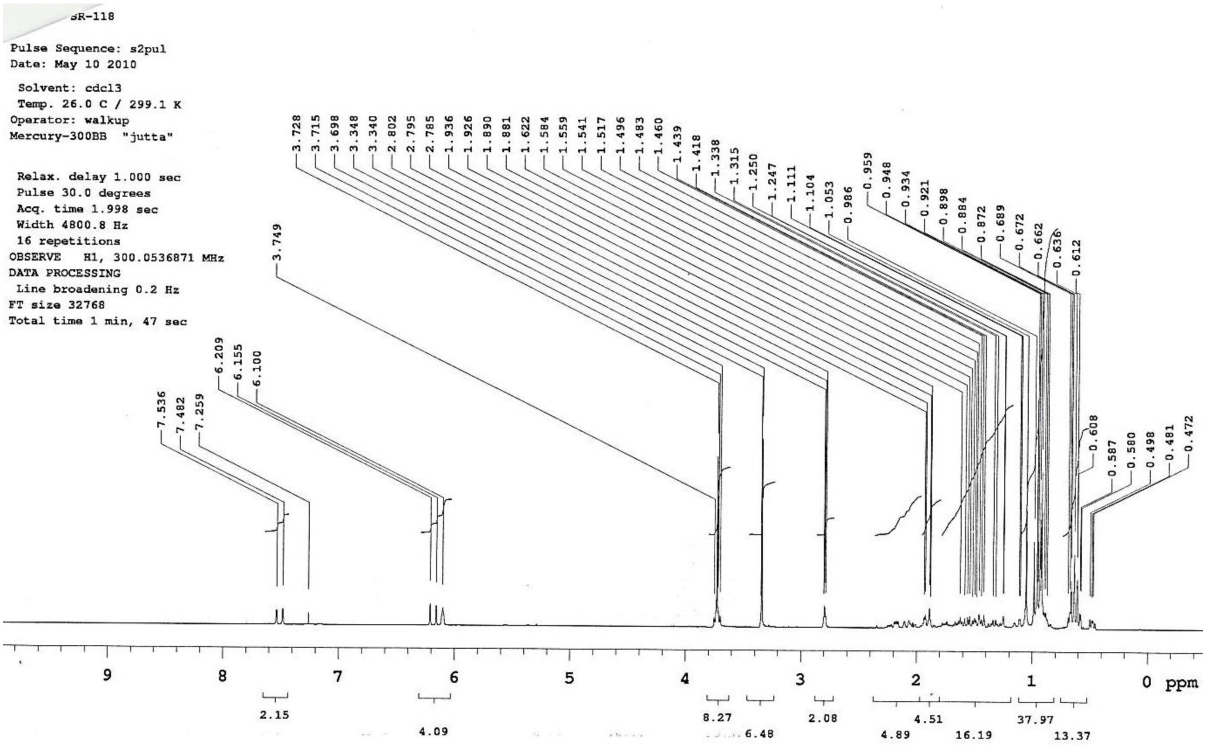

^{13}C-NMR (75 MHz, $CDCl_3$)

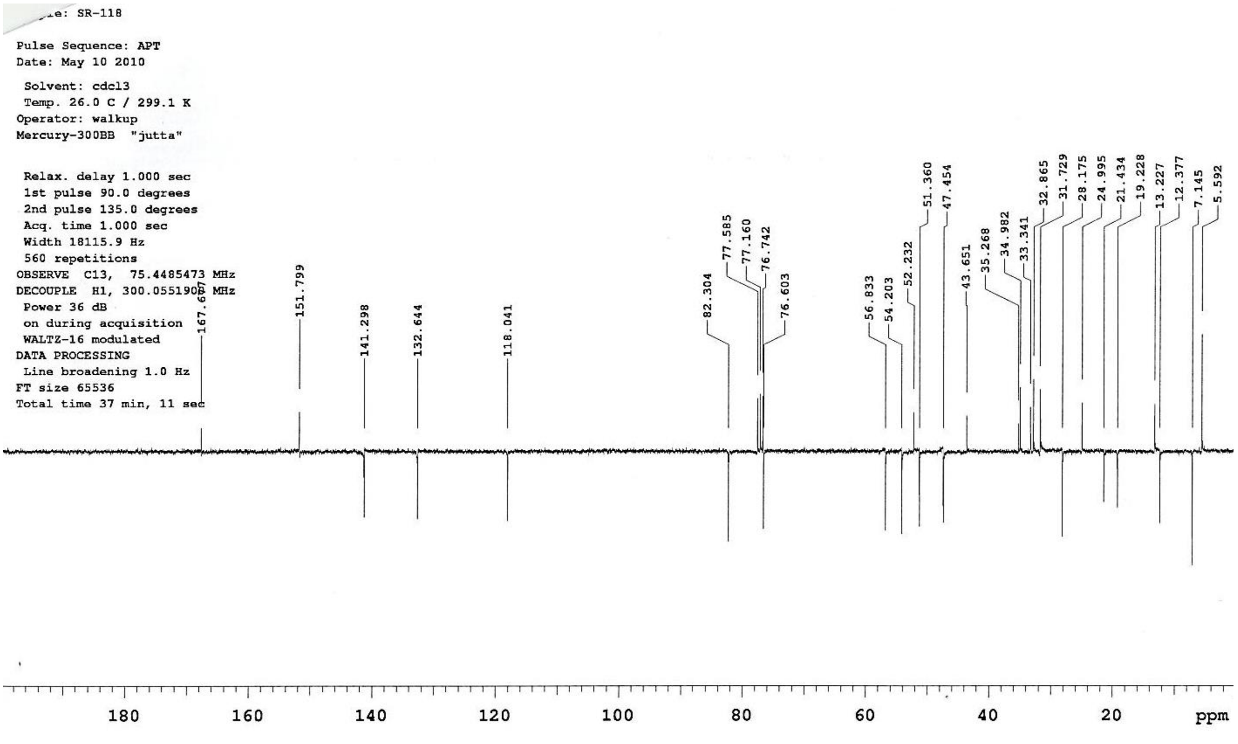

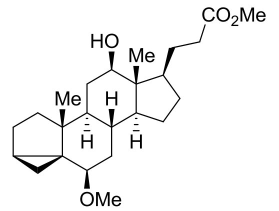

9

^{1}H-NMR (300 MHz, $CDCl_3$)

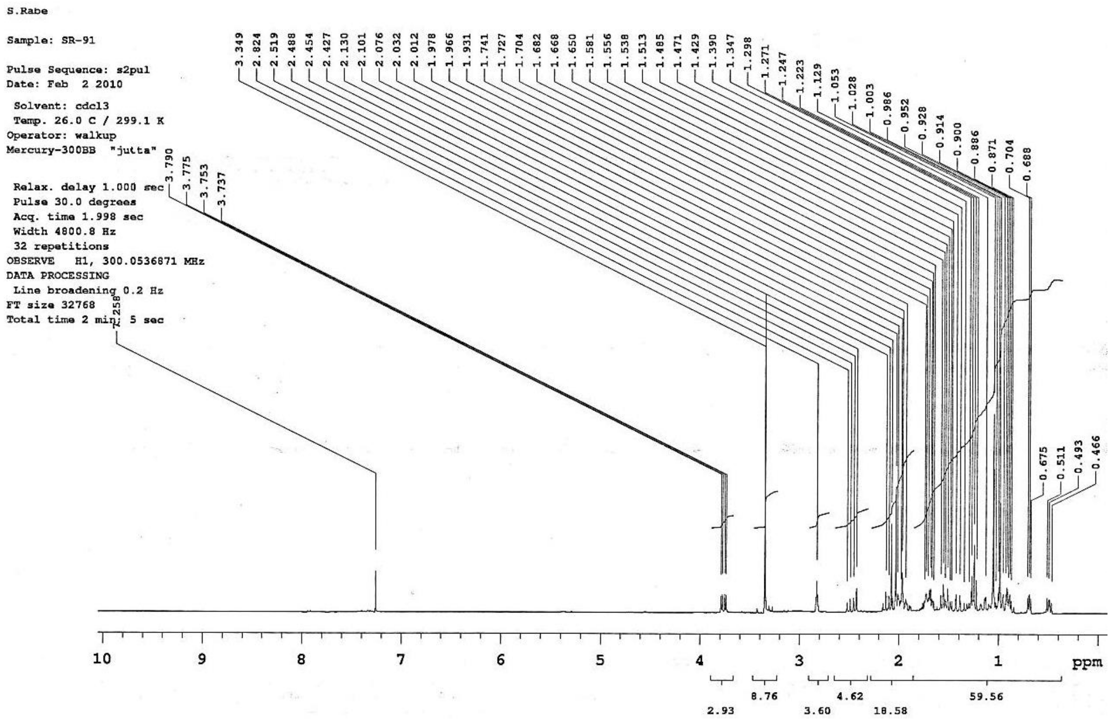

^{13}C-NMR (75 MHz, $CDCl_3$)

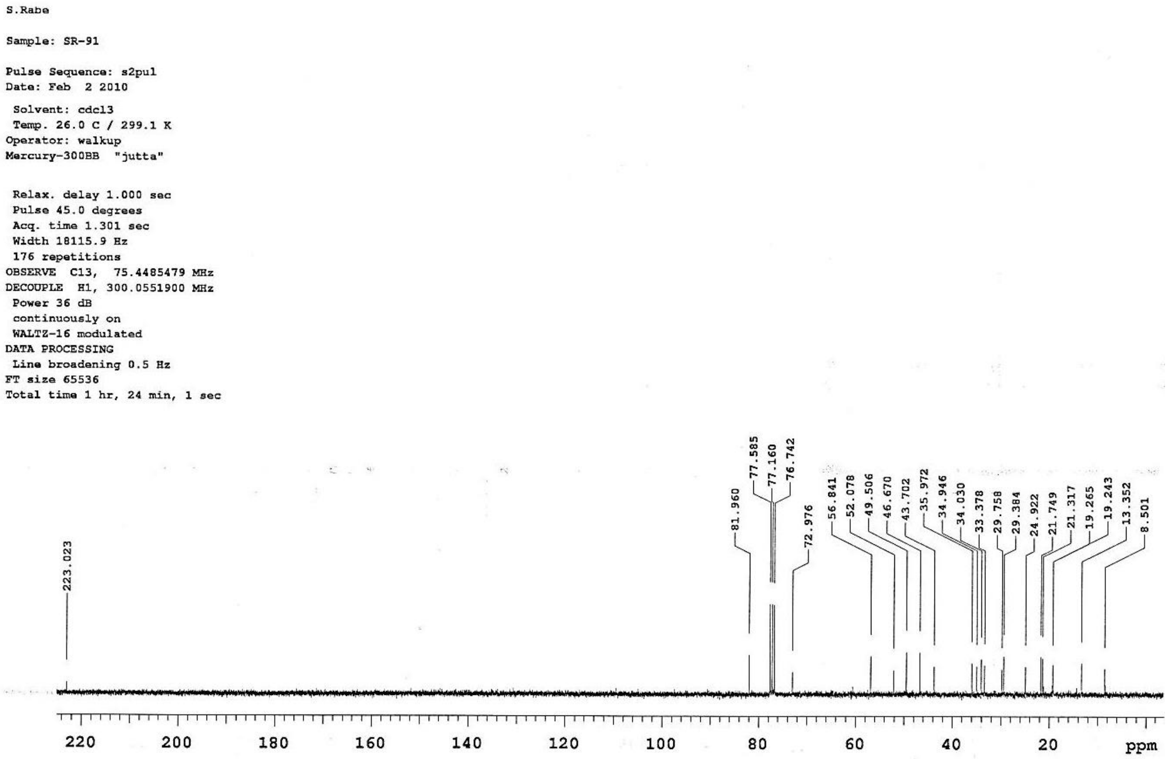

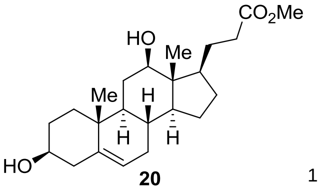

20

^{1}H-NMR (300 MHz, $CDCl_3$)

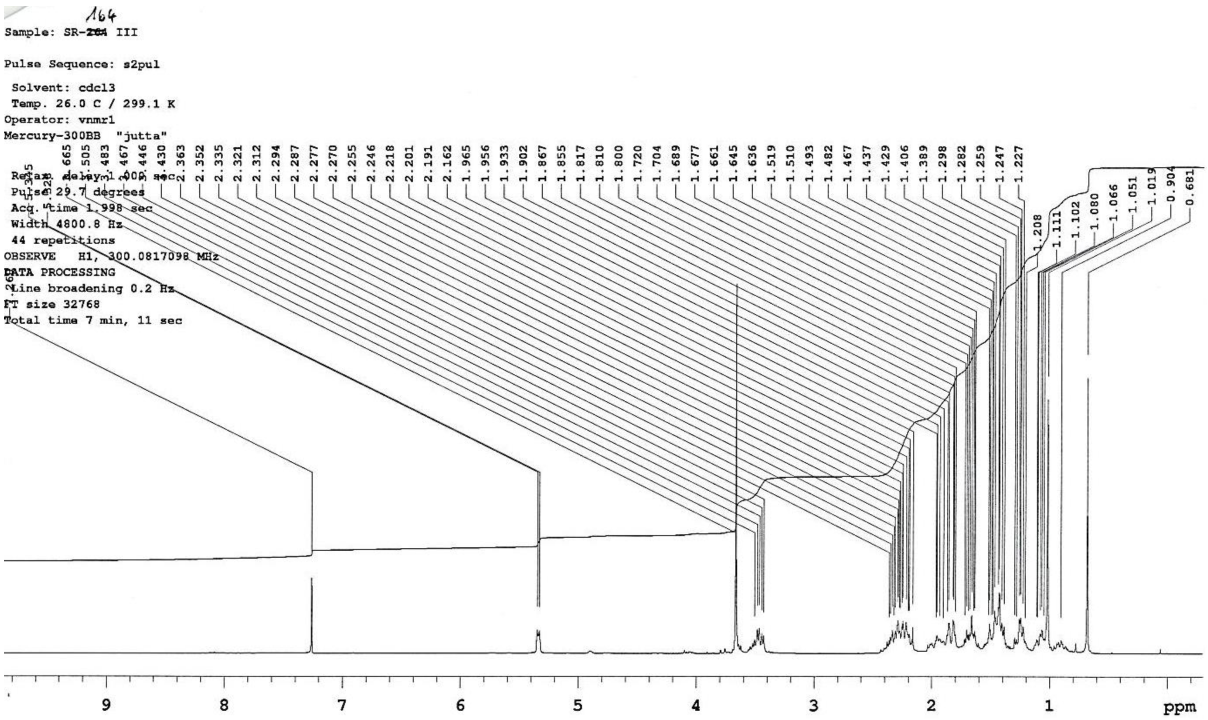

^{13}C-NMR (75 MHz, $CDCl_3$)

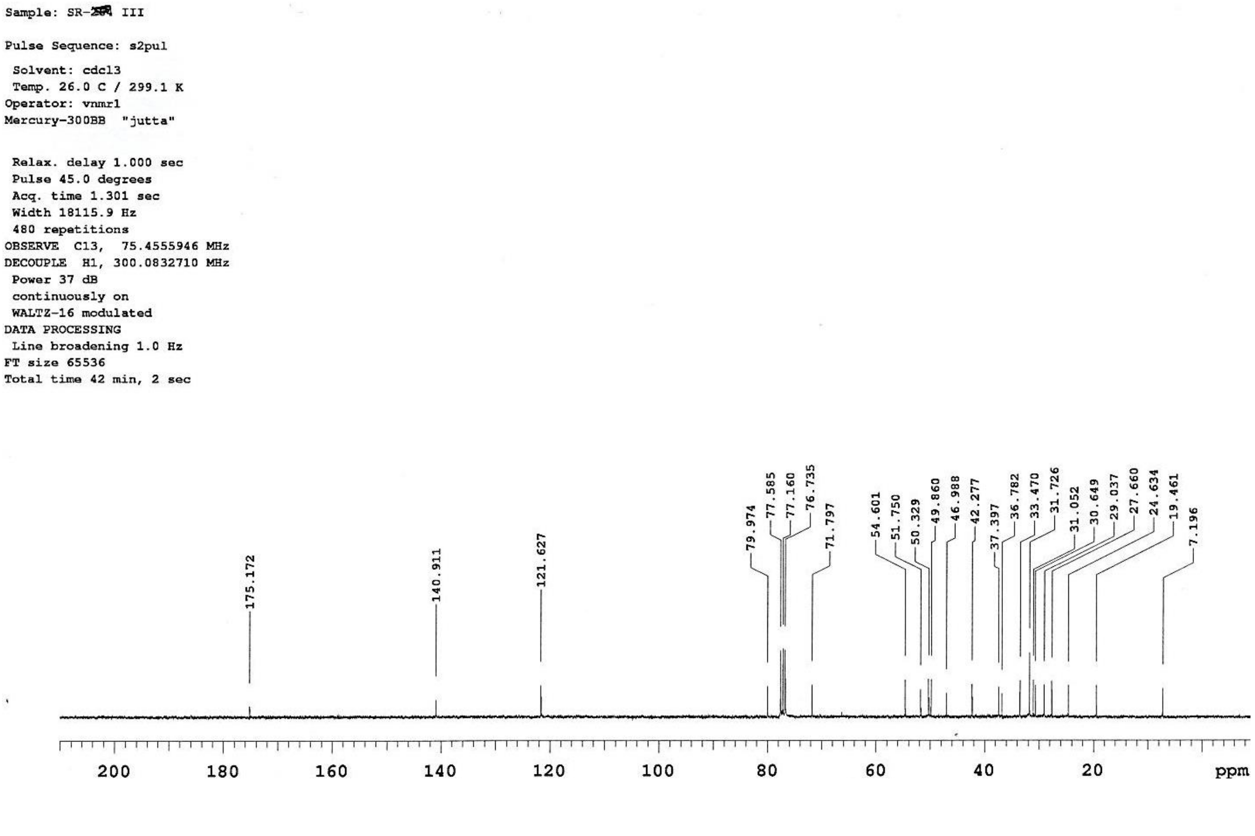

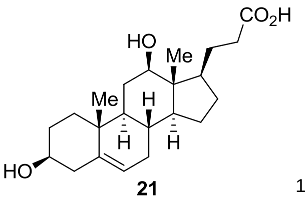

21

^{1}H-NMR (300 MHz, CD_3OD)

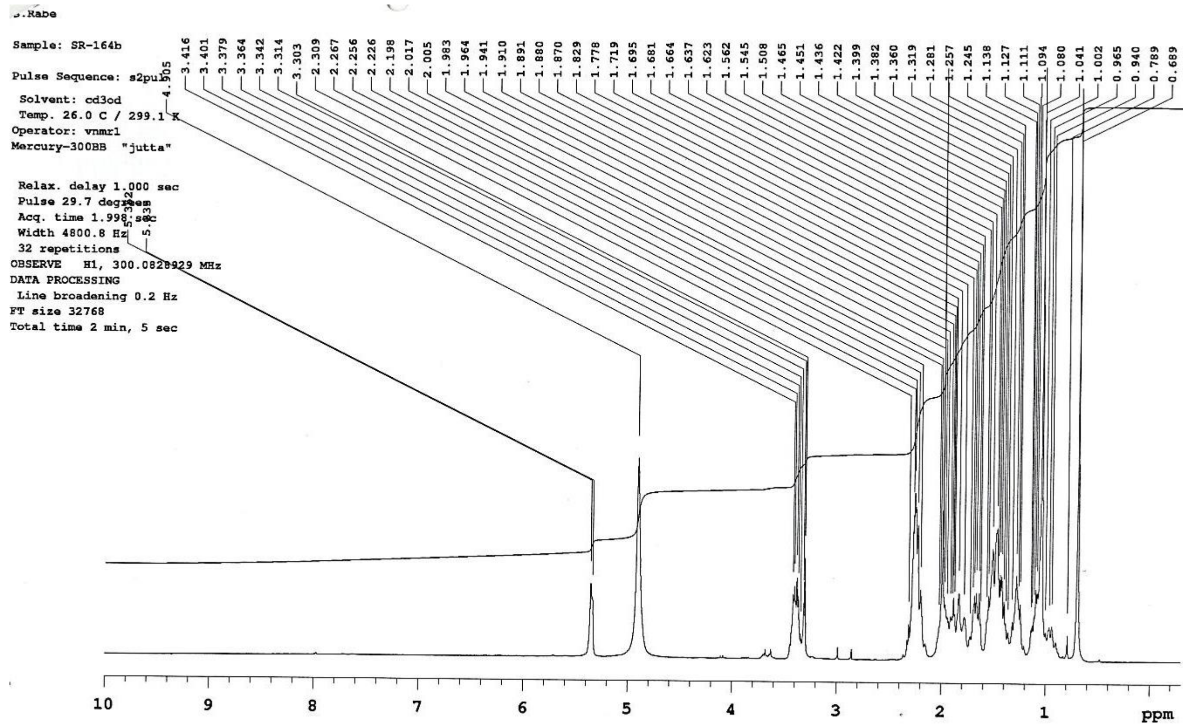

^{13}C-NMR (75 MHz, CD_3OD)

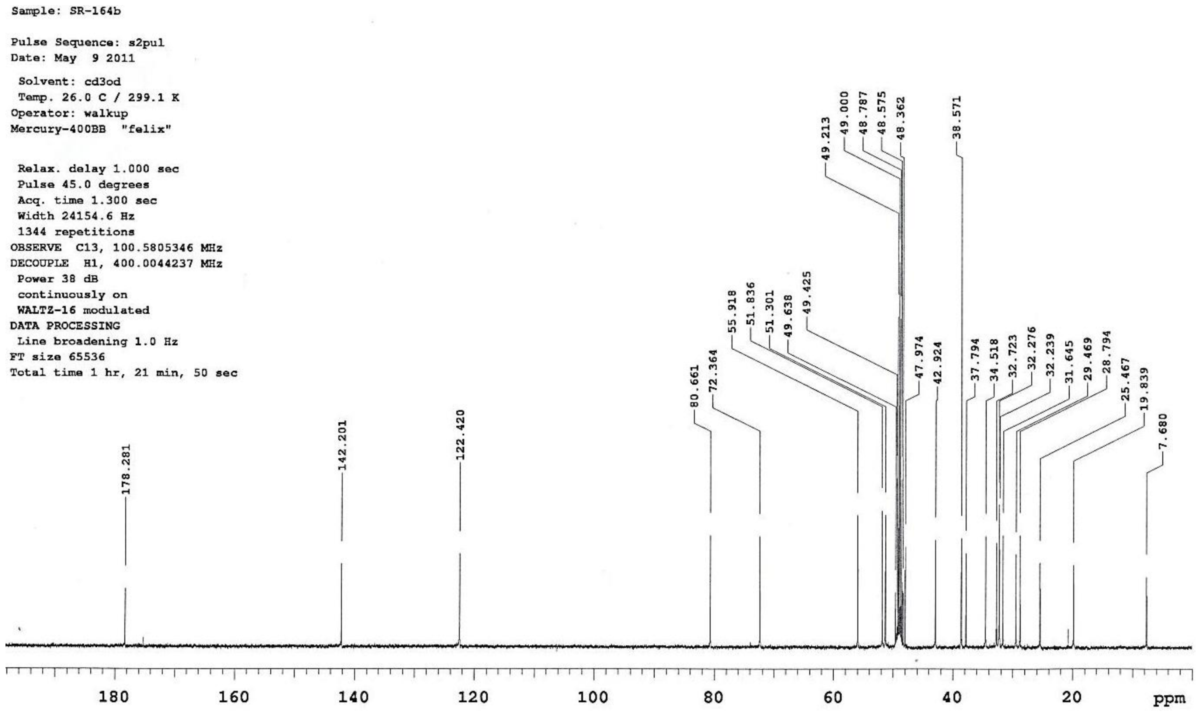

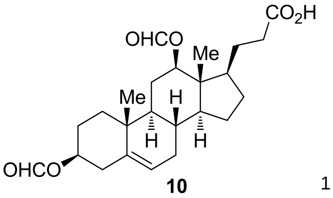

10

^{1}H-NMR (400 MHz, $CDCl_3$)

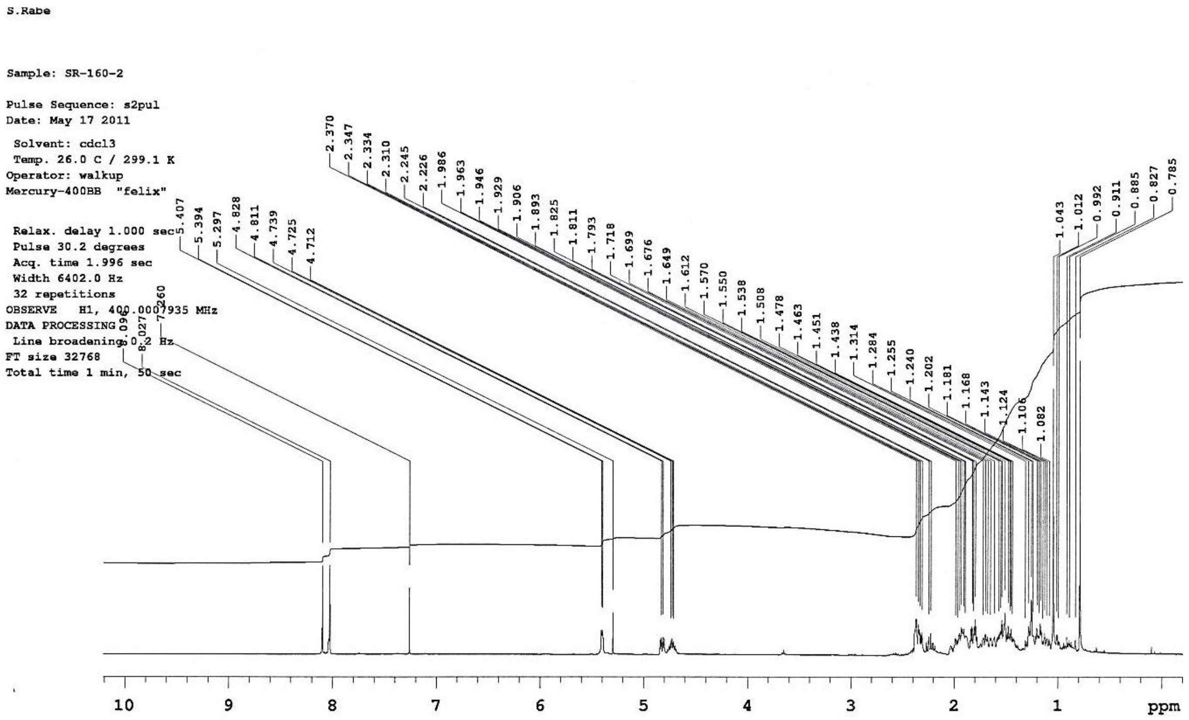

^{13}C-NMR (100 MHz, $CDCl_3$)

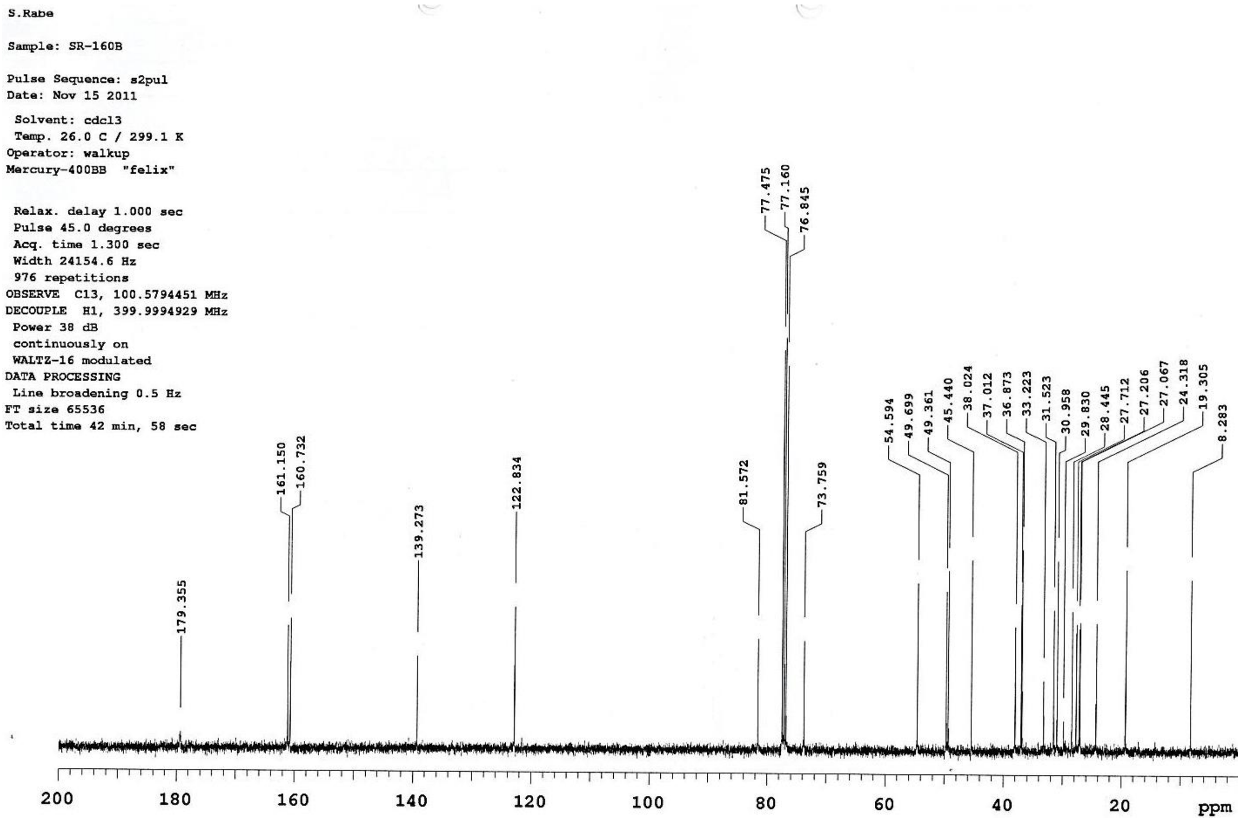

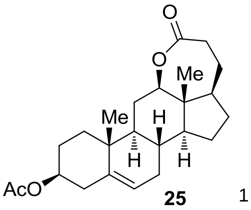

25

^{1}H-NMR (400 MHz, $CDCl_3$)

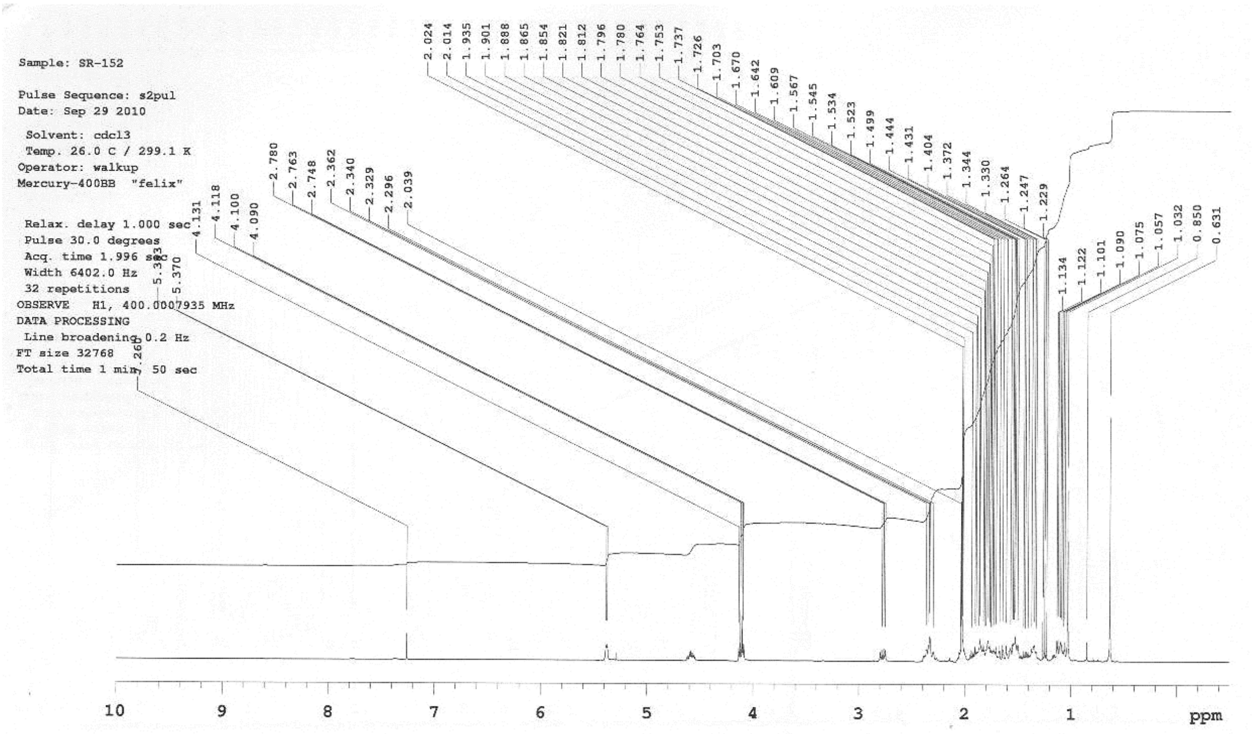

^{13}C-NMR (100 MHz, $CDCl_3$)

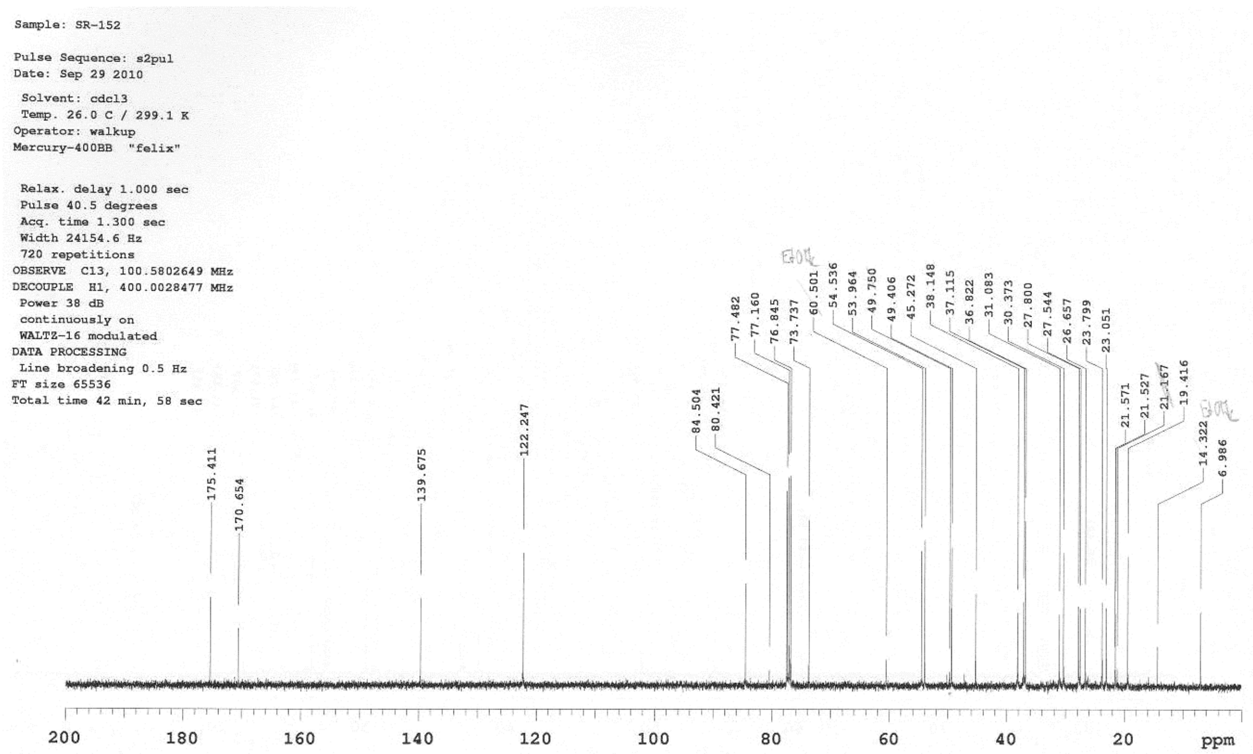

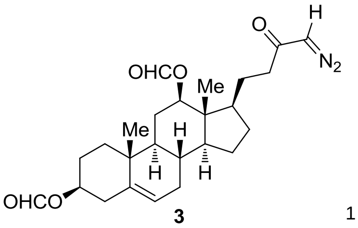

3

^{1}H-NMR (300 MHz, $CDCl_3$)

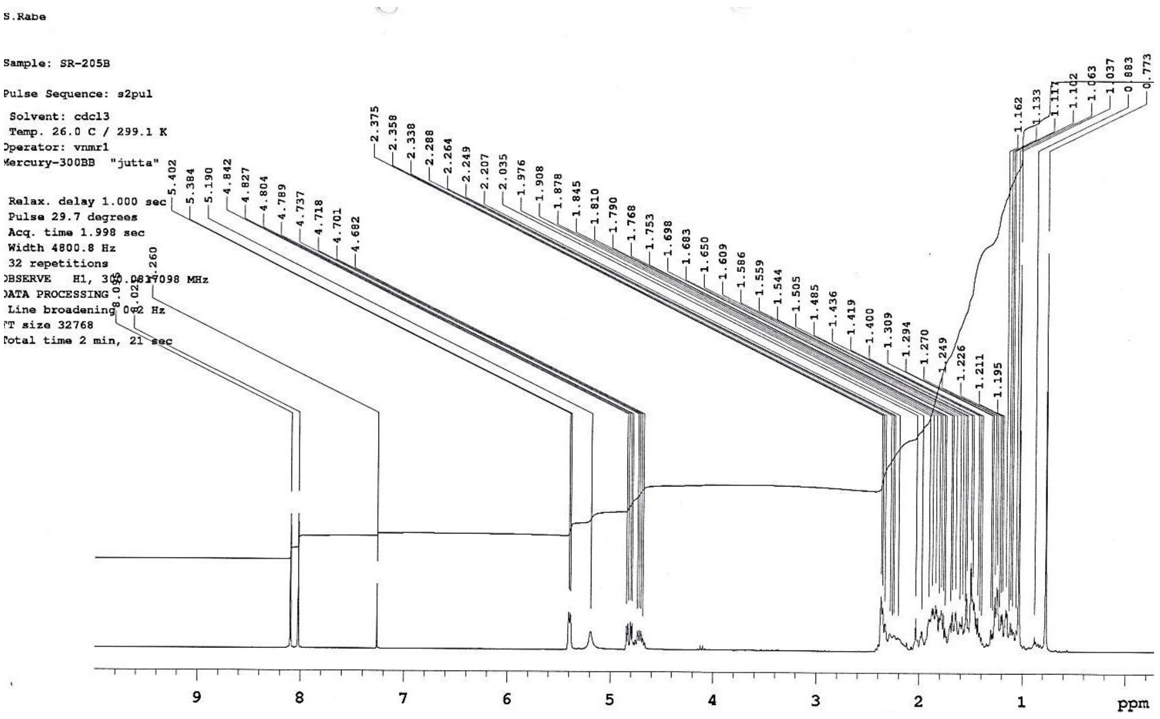

^{13}C-NMR (75 MHz, $CDCl_3$)

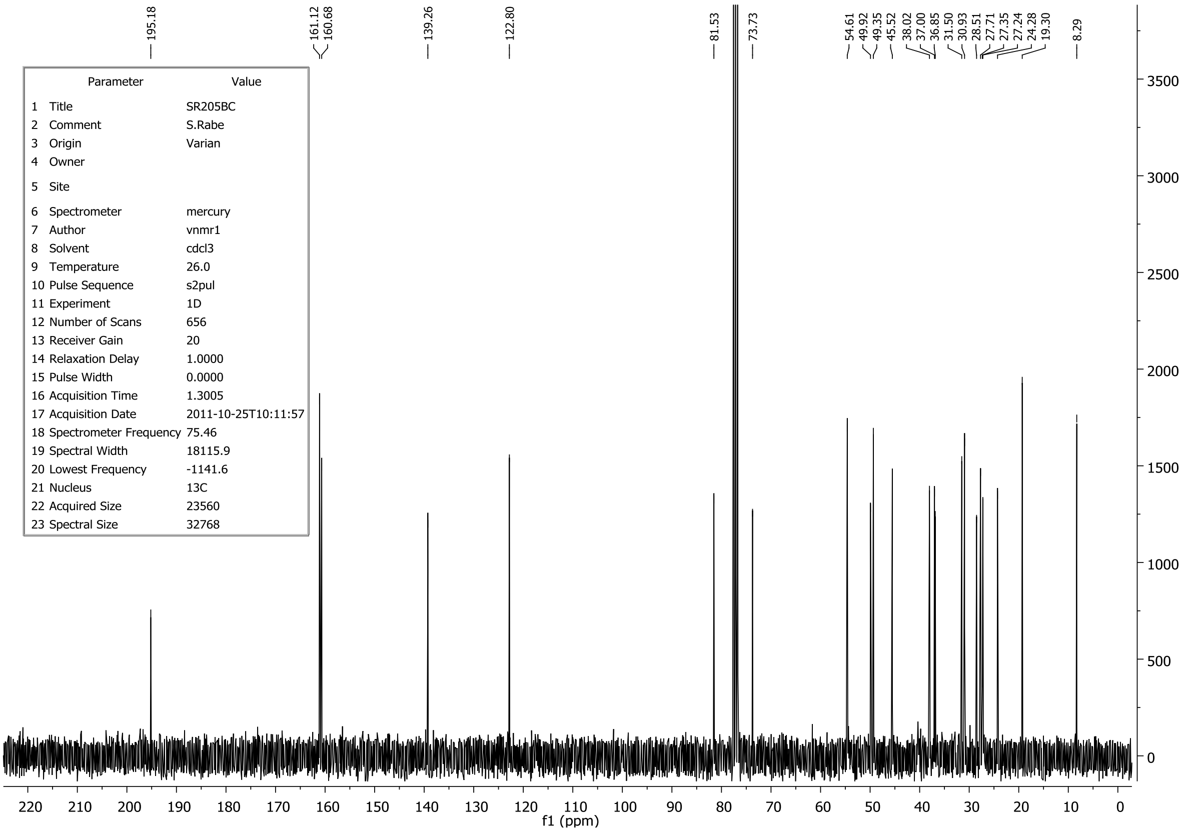

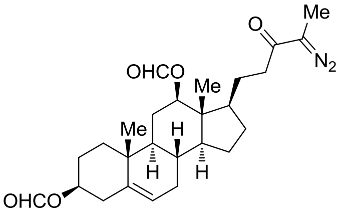

24

^{1}H-NMR (400 MHz, $CDCl_3$)

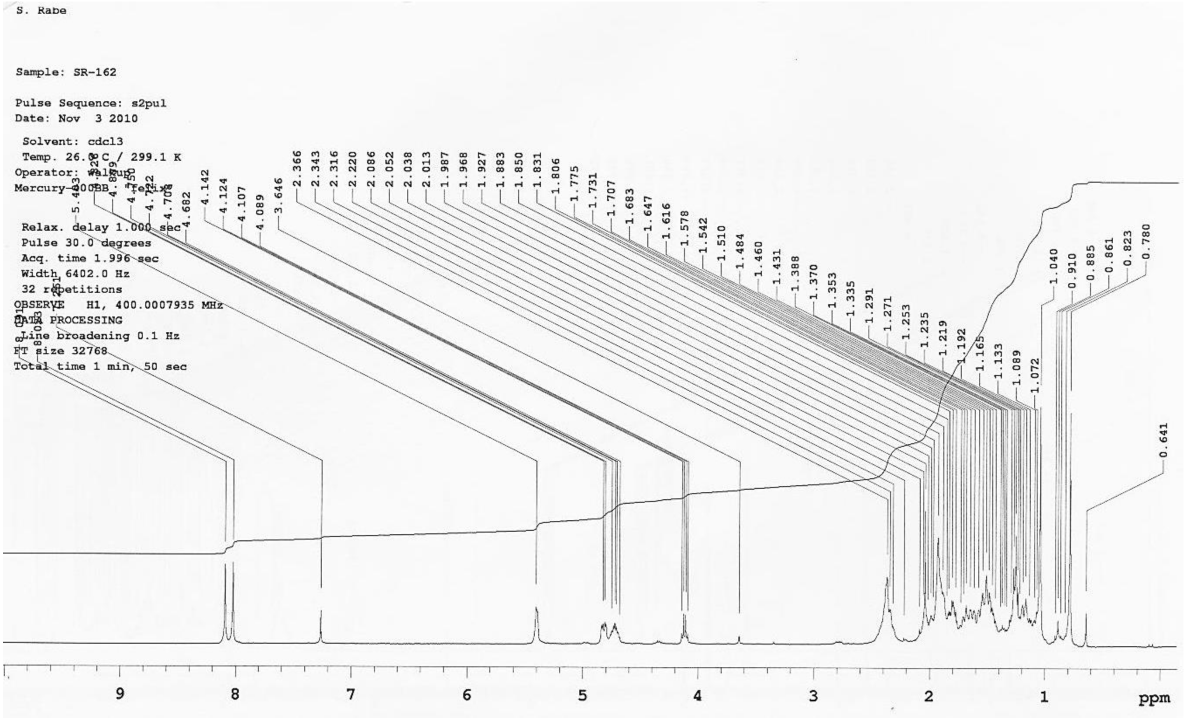

^{13}C-NMR (100 MHz, $CDCl_3$)

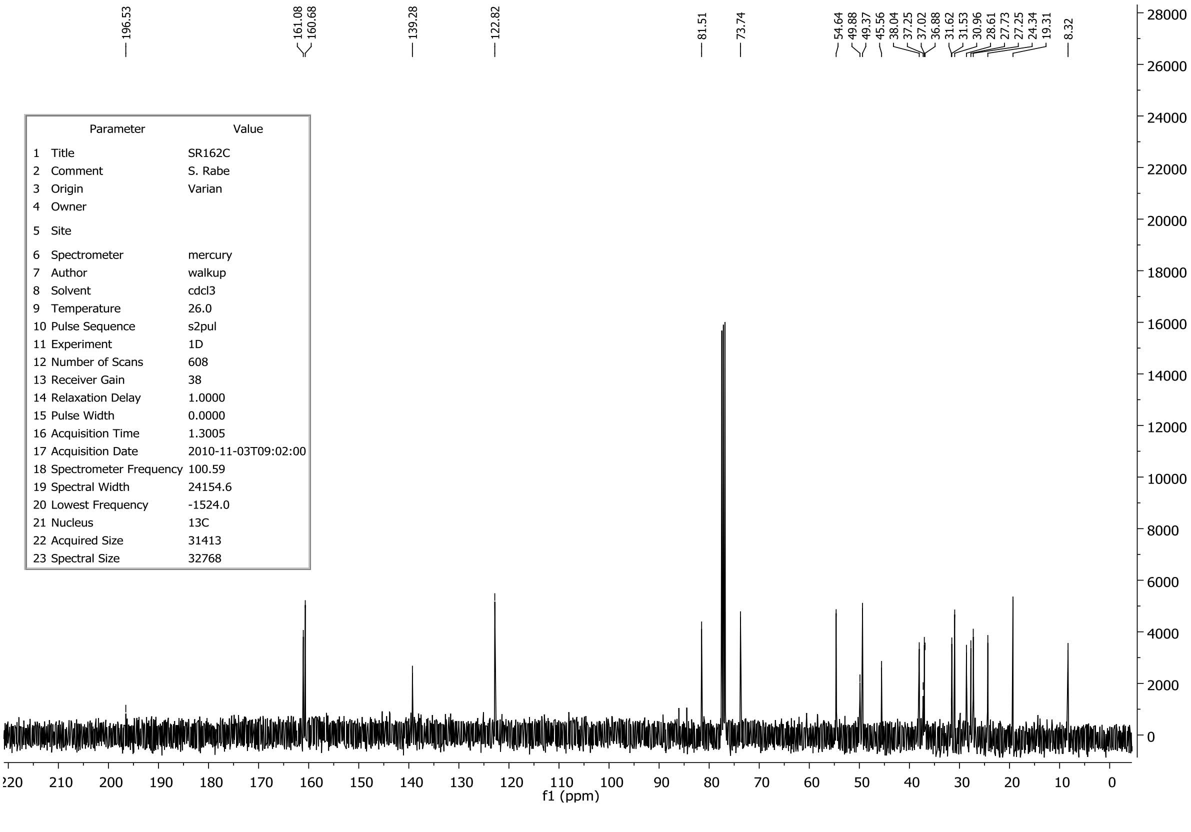

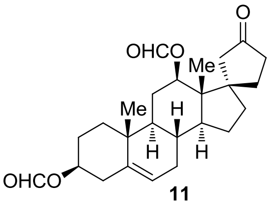

11

^{1}H-NMR (400 MHz, $CDCl_3$)

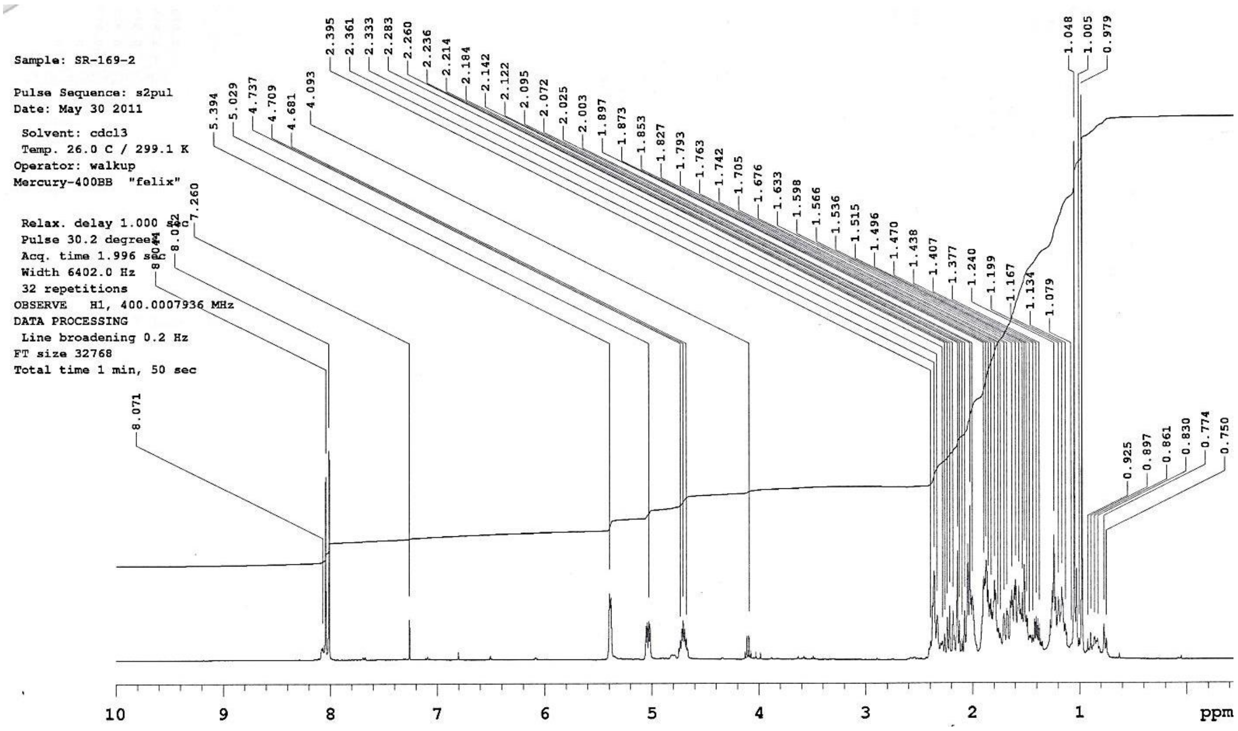

^{13}C-NMR (100 MHz, $CDCl_3$)

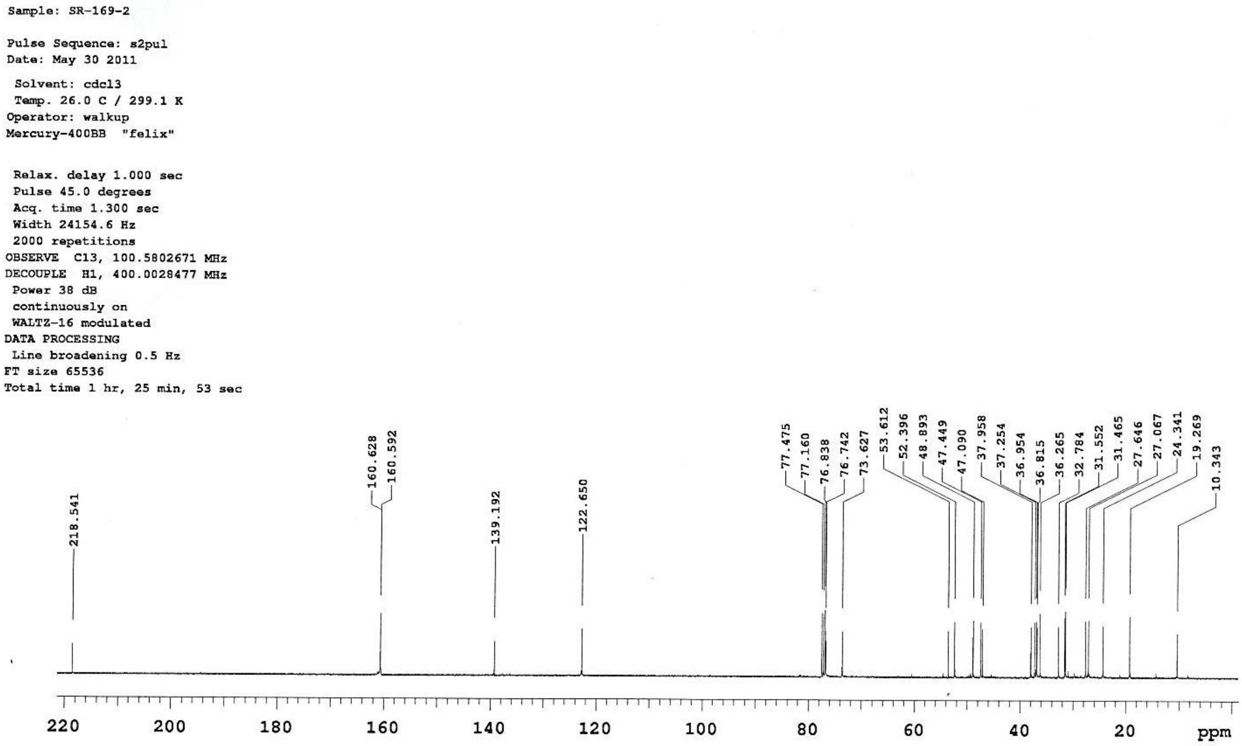

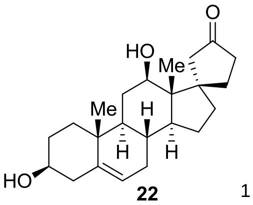

22

^{1}H-NMR (400 MHz, $CDCl_3$)

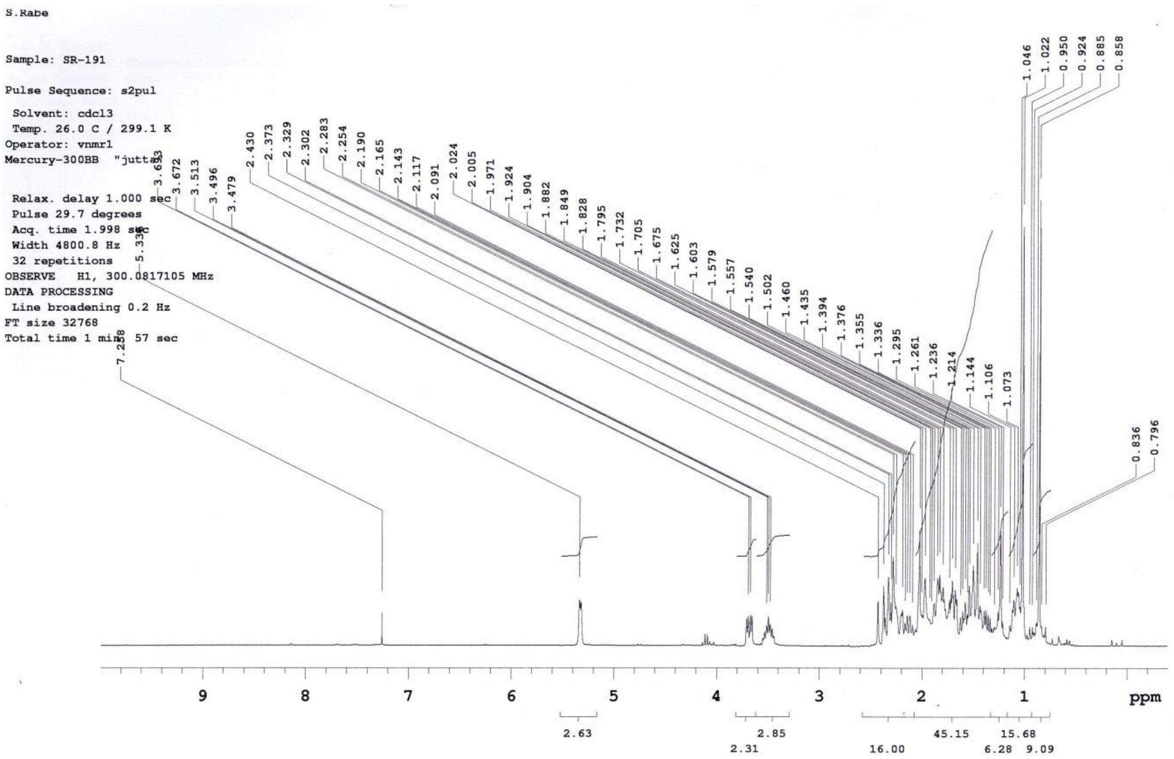

^{13}C-NMR (100 MHz, $CDCl_3$)

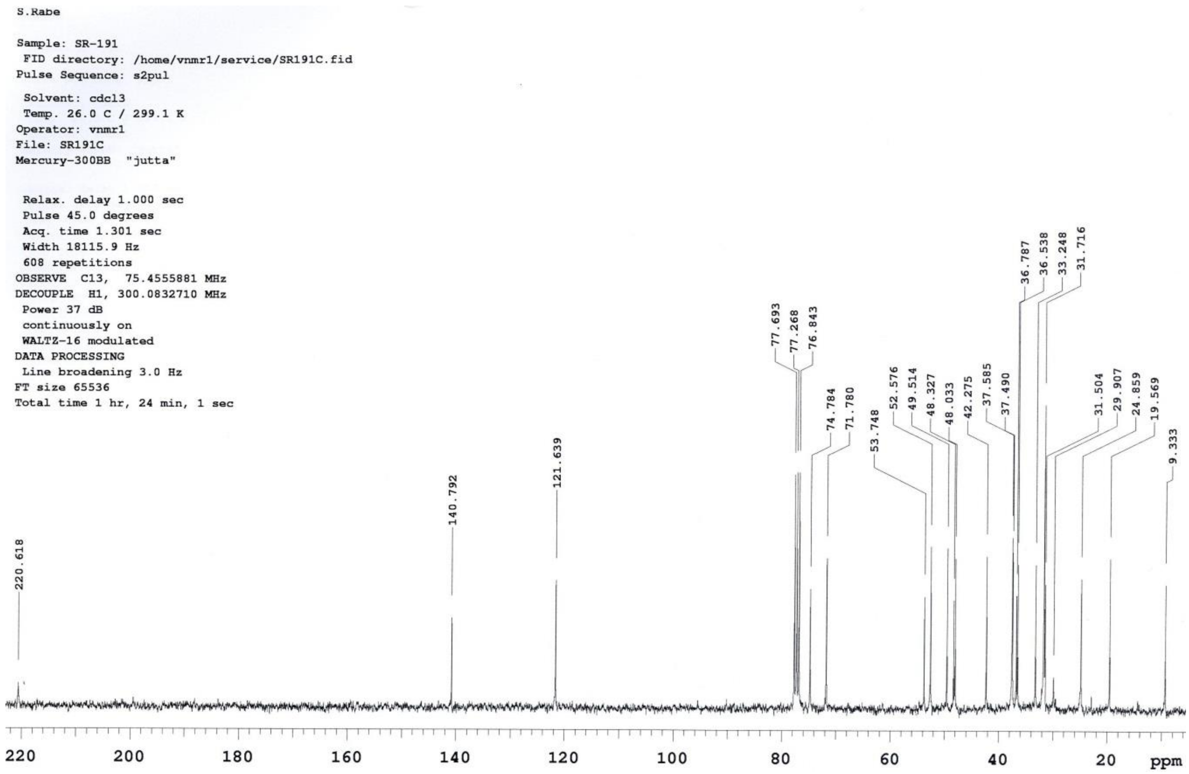

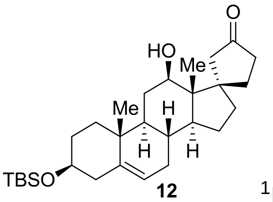

12

^{1}H-NMR (400 MHz, $CDCl_3$)

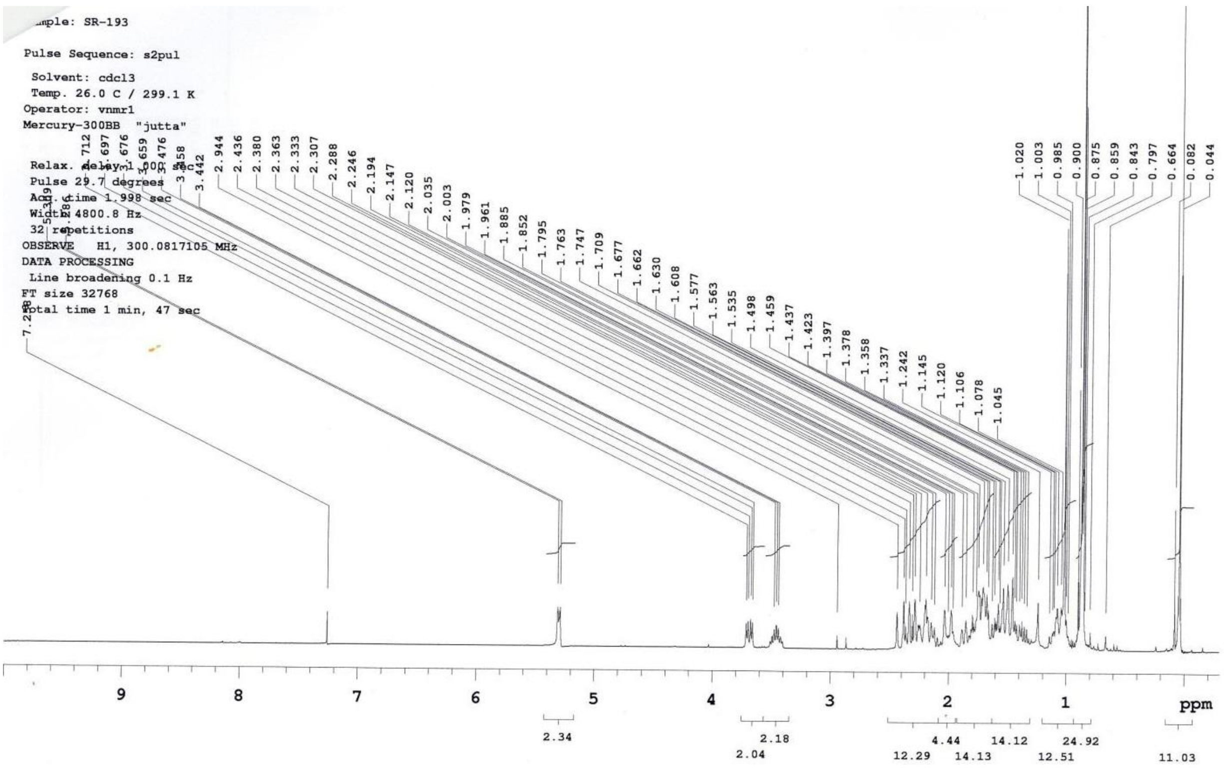

^{13}C-NMR (100 MHz, $CDCl_3$)

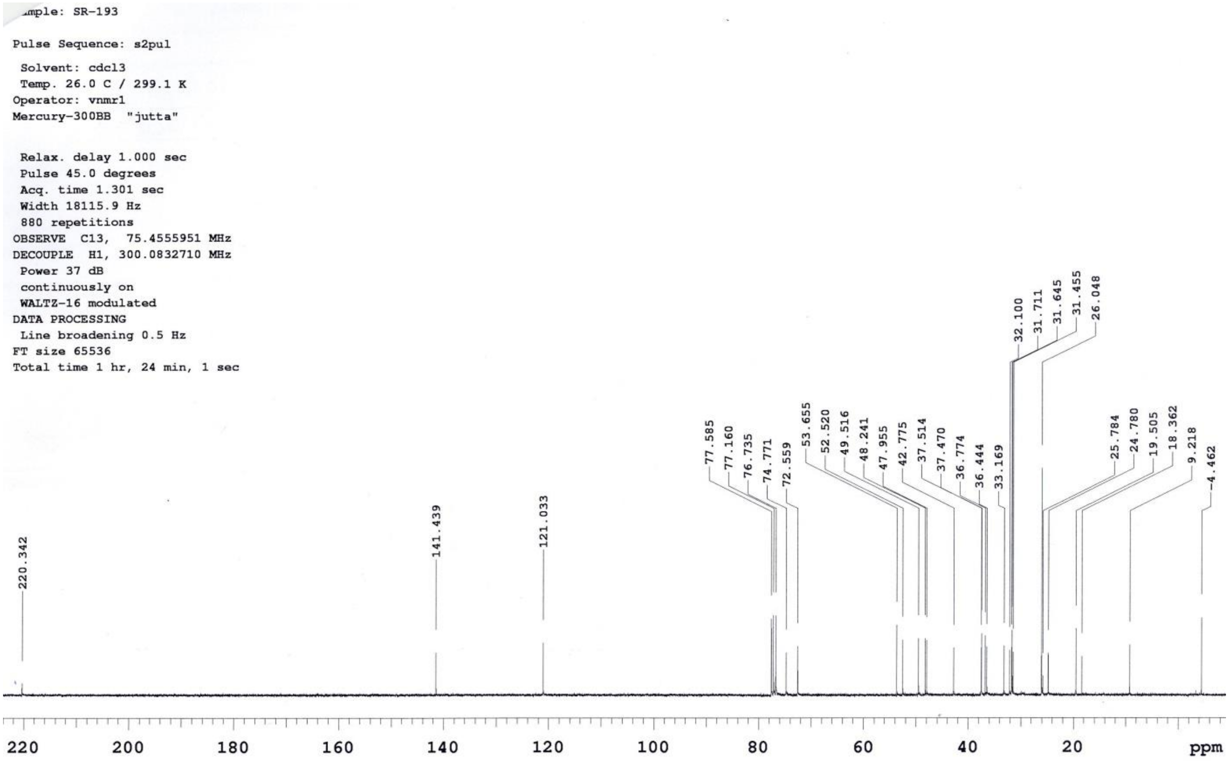

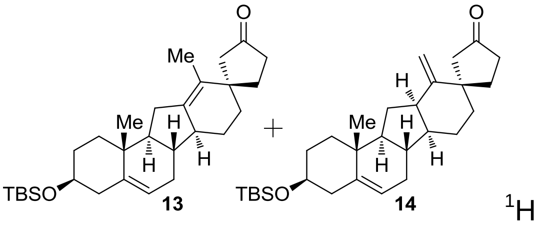

^{1}H-NMR (400 MHz, $CDCl_3$)

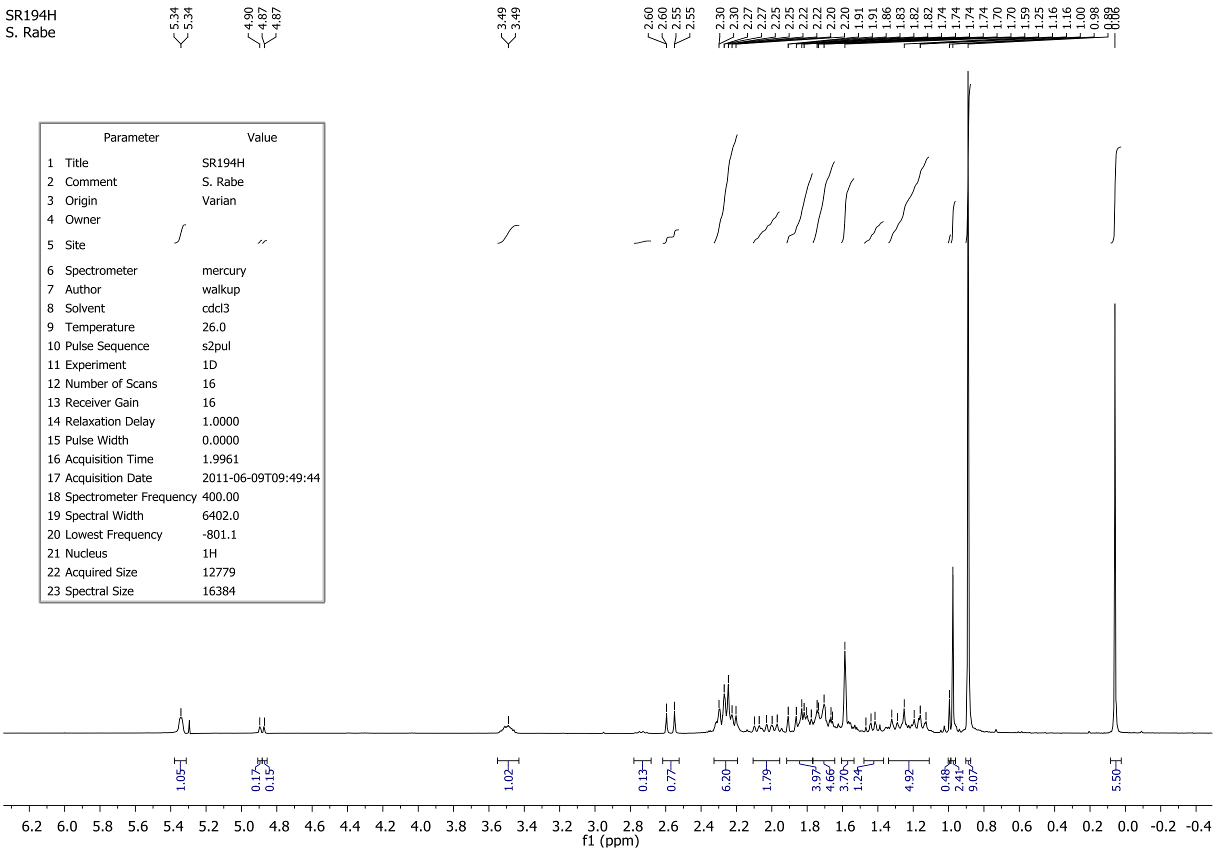

	Parameter	Value
1	Title	SR194H
2	Comment	S. Rabe
3	Origin	Varian
4	Owner	
5	Site	
6	Spectrometer	mercury
7	Author	walkup
8	Solvent	cdcl3
9	Temperature	26.0
10	Pulse Sequence	s2pul
11	Experiment	1D
12	Number of Scans	16
13	Receiver Gain	16
14	Relaxation Delay	1.0000
15	Pulse Width	0.0000
16	Acquisition Time	1.9961
17	Acquisition Date	2011-06-09T09:49:44
18	Spectrometer Frequency	400.00
19	Spectral Width	6402.0
20	Lowest Frequency	-801.1
21	Nucleus	1H
22	Acquired Size	12779
23	Spectral Size	16384

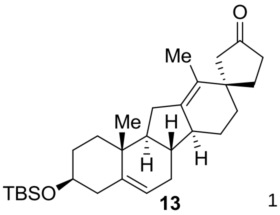

13

^{1}H-NMR (400 MHz, $CDCl_3$)

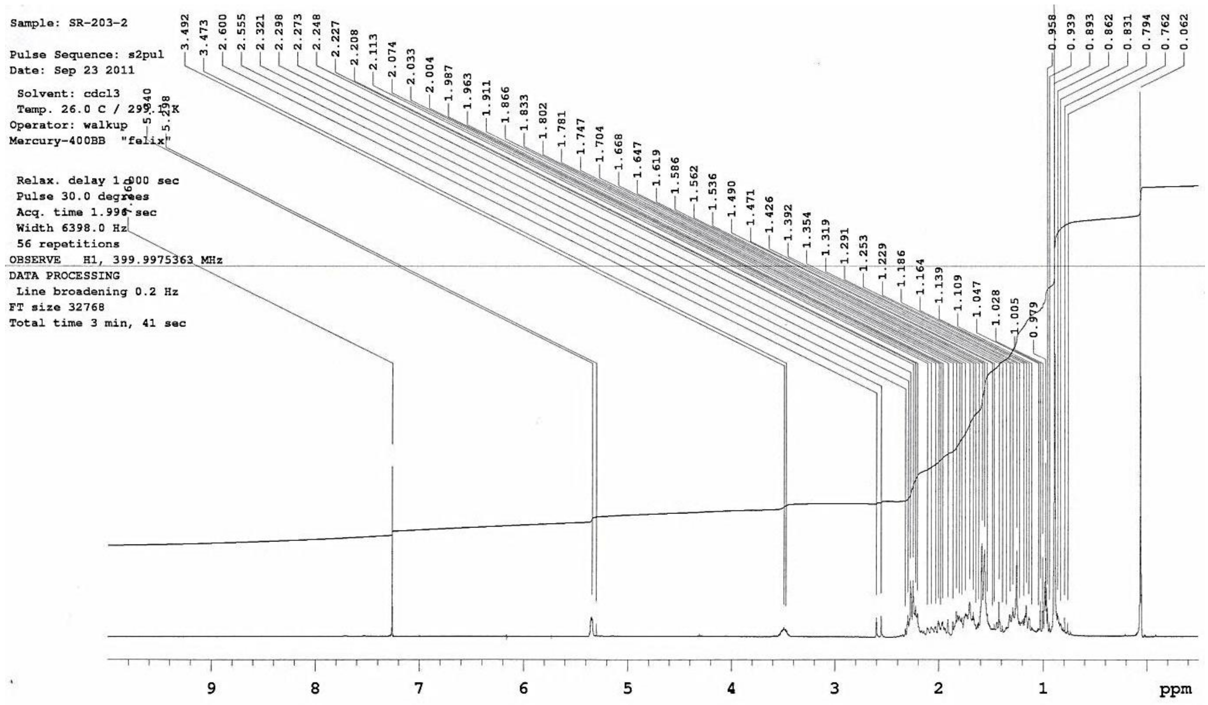

^{13}C-NMR (100 MHz, $CDCl_3$)

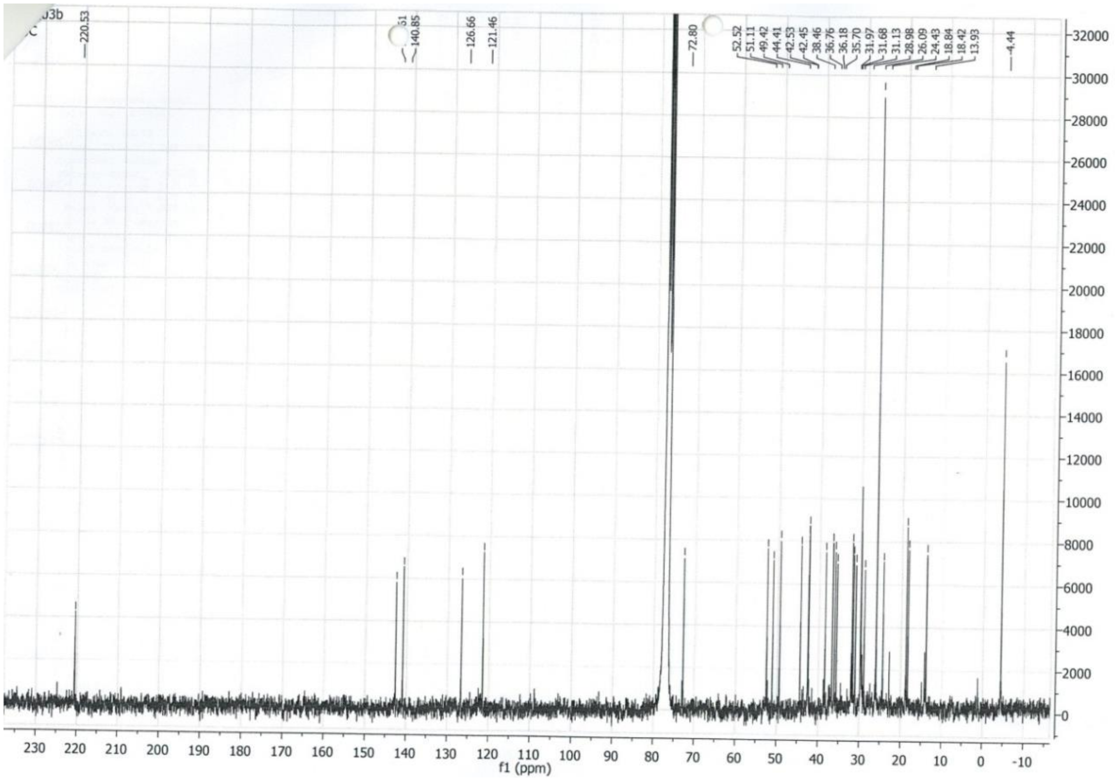

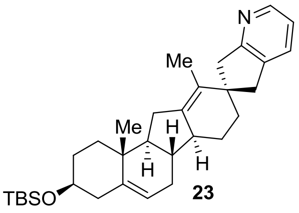

23

^{1}H-NMR (300 MHz, $CDCl_3$)

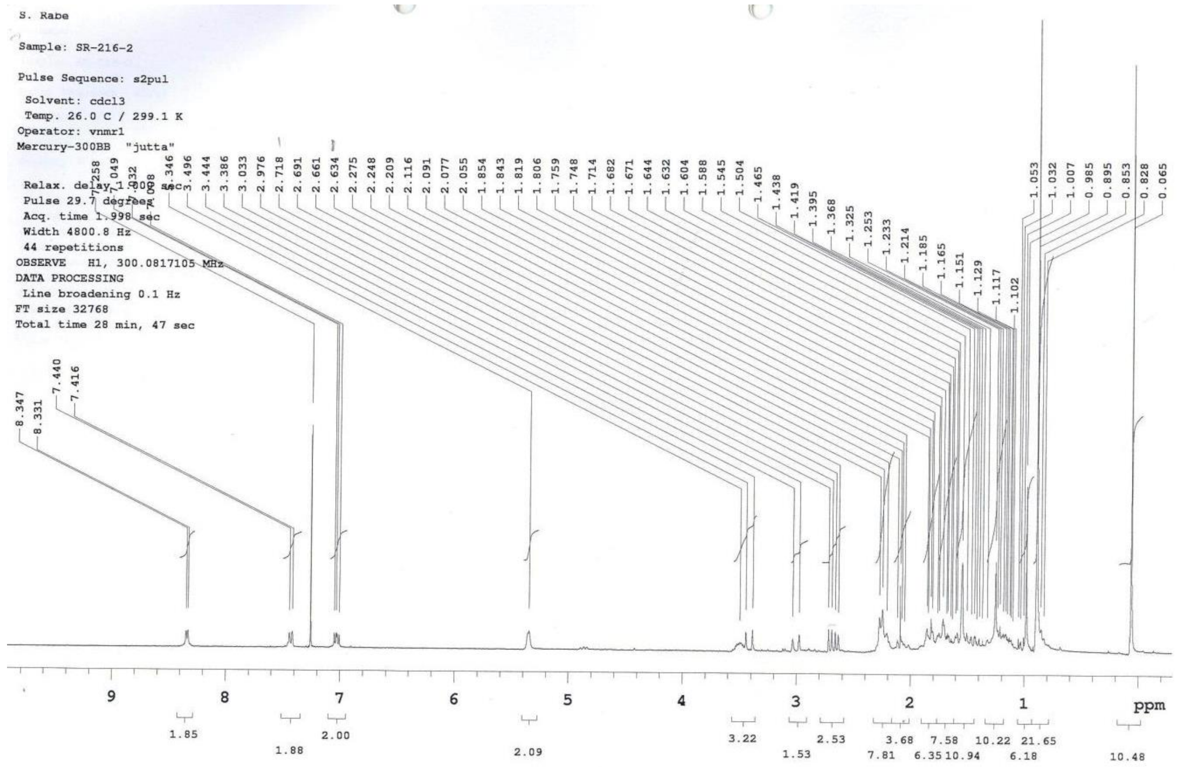

^{13}C-NMR (75 MHz, $CDCl_3$)

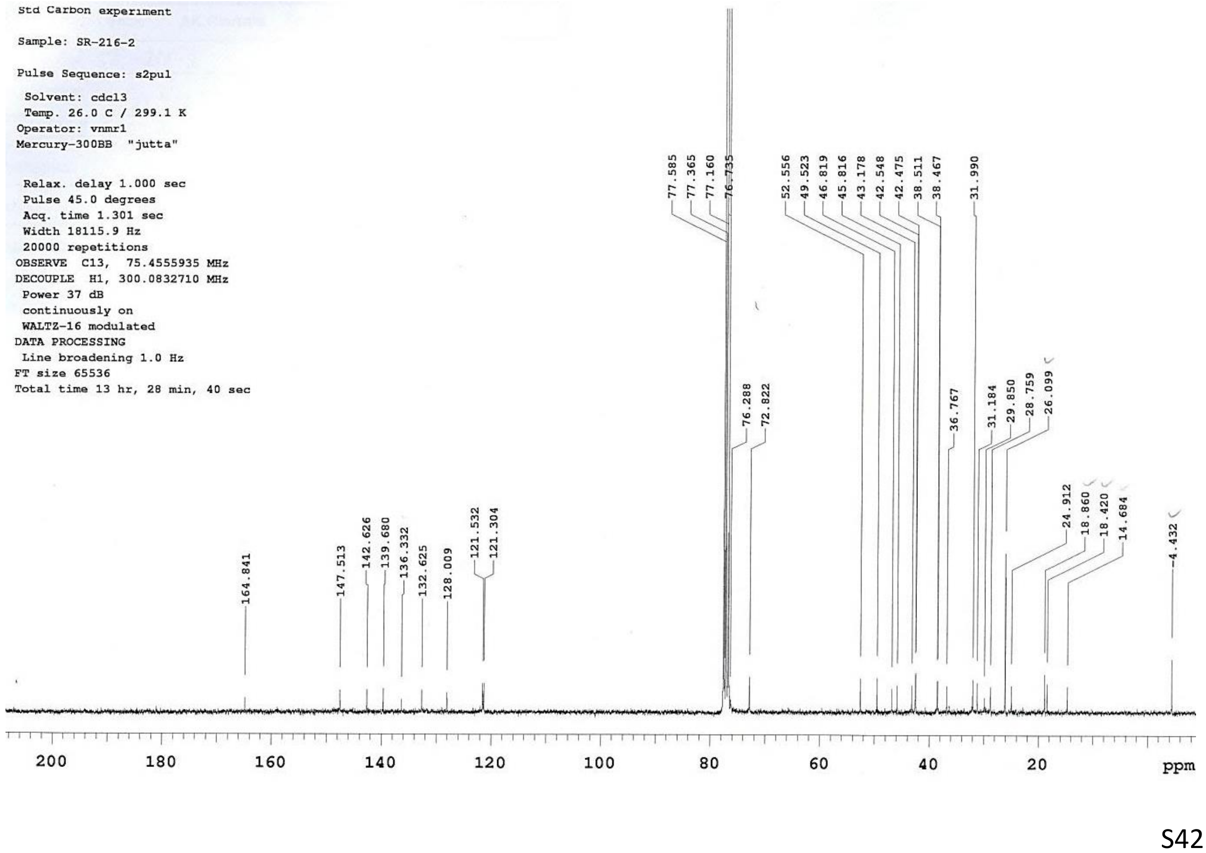

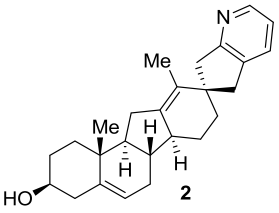

2

^{1}H-NMR (700 MHz, $CDCl_3$)

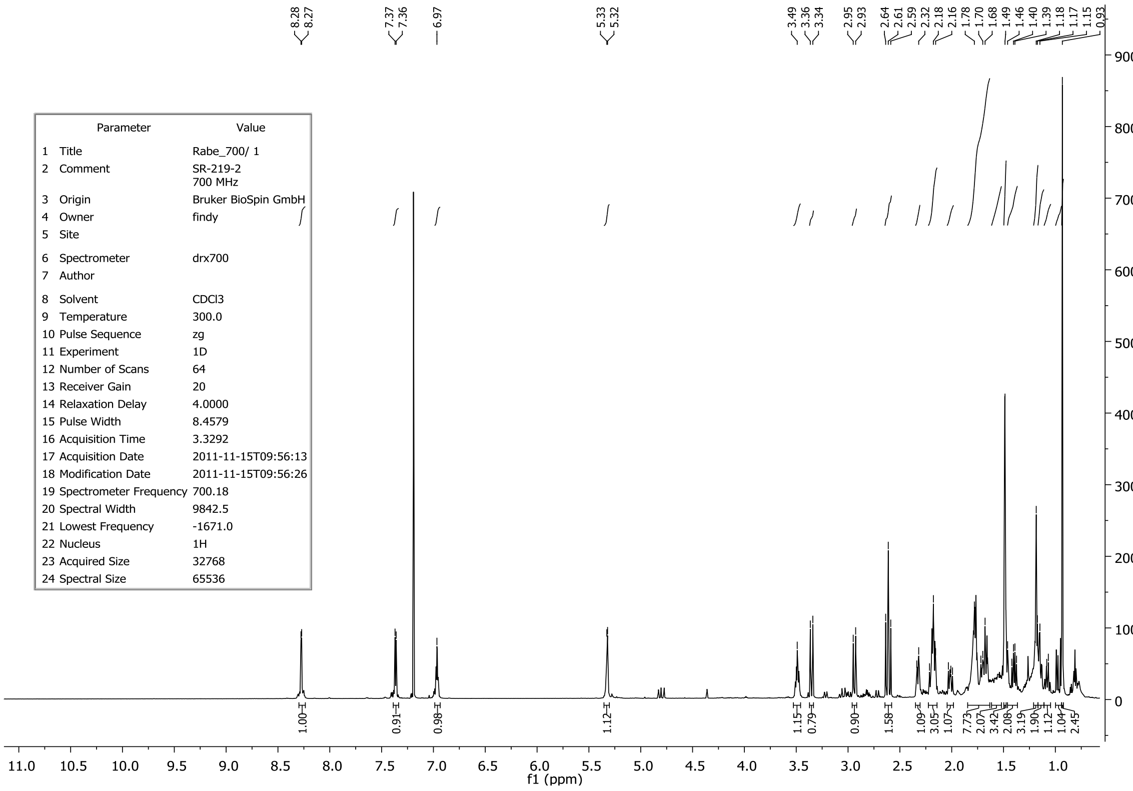

^{13}C-NMR (176 MHz, $CDCl_3$)

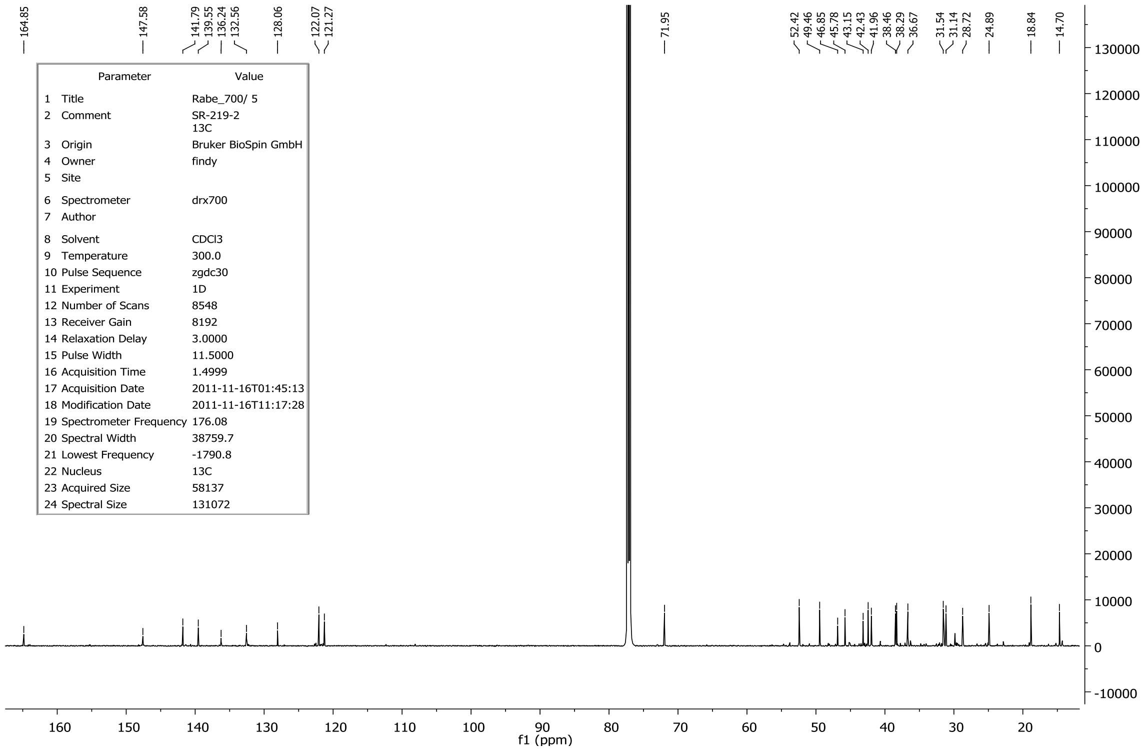

7. Reference

1. Taipale, J.; Chen, J. K.; Cooper, M. K.; Wang, B.; Mann, R. K.; Milenkovic, L.; Scott M. P.; Beachy, P. A. *Nature*, **2000**, *406*, 1005–1009. doi:10.1038/35023008
